# Including planocerid flatworms in the diet effectively toxifies the pufferfish, *Takifugu niphobles*

**DOI:** 10.1038/s41598-018-30696-z

**Published:** 2018-08-17

**Authors:** Shiro Itoi, Hiroyuki Ueda, Riko Yamada, Mitsuki Takei, Tatsunori Sato, Shotaro Oshikiri, Yoshiki Wajima, Ryuya Ogata, Hikaru Oyama, Takahiro Shitto, Kazuya Okuhara, Tadasuke Tsunashima, Eitaro Sawayama, Haruo Sugita

**Affiliations:** 1 Department of Marine Science and Resources, Nihon University, Fujisawa, Kanagawa 252-0880 Japan; 2 R&D Division, Marua Suisan Co., Ltd. Kamijima, Ehime, 794-2410 Japan

## Abstract

Beginning with the larval stages, marine pufferfish such as *Takifugu niphobles* contain tetrodotoxin (TTX), an extremely potent neurotoxin. Although highly concentrated TTX has been detected in adults and juveniles of these fish, the source of the toxin has remained unclear. Here we show that TTX in the flatworm *Planocera multitentaculata* contributes to the toxification of the pufferfish throughout the life cycle of the flatworm. A species-specific PCR method was developed for the flatworm, and the specific DNA fragment was detected in the digesta of wild pufferfish adults. Predation experiments showed that flatworm larvae were eaten by the pufferfish juveniles, and that the two-day postprandial TTX content in these pufferfish was 20–50 μg/g. Predation experiments additionally showed flatworm adults were also eaten by pufferfish young, and after two days of feeding, TTX accumulated in the skin, liver and intestine of the pufferfish.

## Introduction

Tetrodotoxin (TTX), named after the Piscean order Tetradontiformes, is also known as pufferfish toxin after one of the main group of fishes in the order. TTX is a non-peptidic potent neurotoxin that specifically binds to voltage-gated sodium channels on excitable membranes of muscle and nerve tissues^1,2^. Apart from pufferfish, it has been found in various taxonomic groups of animals, including amphibians (e.g., California newt *Taricha torosa*^3^; Costa Rica frog *Atelopus* spp.^4^), fishes (e.g., goby *Yongeichthys criniger*^5^), cephalopods (e.g., blue-ringed octopus *Hapalochlaena maculosa*^6^), gastropods (e.g., the Japanese ivory shell *Babylonia japonica*^7^; the opisthobranch *Pleurobranchaea maculata*^8,9^), crustaceans (e.g., the xanthid crab *Atergatis floridus*^10^), starfishes (e.g., *Astropecten* spp.^11,12^), flatworms (e.g., *Planocera* spp.^13–16^; *Stylochoplana* sp.^17^) and ribbonworms (e.g., *Cephalothrix* sp.^14,18^). Furthermore, TTX production has been observed in several species of bacteria that are symbiotic with pufferfish and are obtained from their prey^19–22^.

It has generally been understood that the *Takifugu* pufferfish species accumulate TTX via the food web, consisting of several steps, starting with TTX producing marine bacteria^22,23^. This speculation has been supported by several studies that have shown that non-toxic pufferfish are obtained when they are artificially cultured after hatching and fed non-toxic diets, and that these cultured non-toxic pufferfish become toxic when administered TTX orally^24–28^. However, tracing the toxification via the food chain exclusively to marine bacteria is unlikely to account for the amount of TTX in the pufferfish body, as bacteria produce minute amounts of toxin^21,29–32^. However, TTX levels produced by bacterial cultures under potentially non-optimum conditions might be significantly less than those synthesized by bacteria colonizing pufferfish organs. Indeed, the issue remains unsettled, and the toxification process in pufferfish still remains unclear. Recently, our lab showed that the pufferfish *Takifugu niphobles* fed on the naturally TTX-laden eggs of another pufferfish *Takifugu pardalis*, suggesting that *T. niphobles* effectively increased its own toxicity by feeding on the toxic eggs from another toxic species^33^. That report also proposed that TTX is progressively concentrated in a “TTX loop” that includes TTX-bearing organisms at higher trophic levels in the food web^33^.

Other studies from our lab have shown that although the source of TTX is largely unidentified in wild-caught juveniles of the pufferfish *T. niphobles*, larval pufferfish do contain minute amounts of TTX provided by their mother^34,35^. We have also shown that the larvae of the planocerid flatworm *Planocera multitentaculata* possess highly concentrated TTX in the body, and eggs are laid May through July, coinciding with the spawning season of the pufferfish *T. niphobles* in rocky inshore waters^36^. Therefore, in the present study, we investigated the contribution of the planocerid flatworm to the toxification of the pufferfish *T. niphobles* throughout its life history.

## Results

### Toxicity of the wild pufferfish juveniles

The concentration and total amount of TTX in the juveniles from Oiso (obtained at three different time periods) and Katase are shown in Table 1. These values correspond to 3.16 ± 1.37 mouse unit (MU)/g (2.65 ± 2.16 MU/individual) in August, 2010 (n = 12); 3.32 ± 1.35 MU/g (4.57 ± 2.14 MU/individual) in August, 2011 (n = 9); and 21.68 ± 21.67 MU/g (2.50 ± 4.92 MU/individual) in July, 2015 (n = 21) for the juveniles obtained from Oiso, while for those from Katase (n = 24) in July, 2016 and 2017, the numbers shown translate to 54.75 ± 50.25 MU/g (11.70 ± 15.61 MU/individual) and 64.85 ± 38.21 MU/g (14.47 ± 12.60 MU/individual), respectively.

**Table 1:** Toxicity (concentration) and amount of TTX in wild pufferfish *Takifugu niphobles* juveniles. Data are represented mean ± standard deviation.

Date	Locality	No. of individuals	Total length (mm)	Body weight (g)	Toxicity (ng/g)	TTX amount (ng/ind.)
2010 August	Oiso (35°18′N, 139°19′E)	12	27.6 ± 6.2	0.8 ± 0.5	694 ± 301	583 ± 475
2011 August	Oiso (35°18′N, 139°19′E)	9	34.1 ± 1.8	1.4 ± 0.2	731 ± 298	1006 ± 471
2015 July	Oiso (35°18′N, 139°19′E)	21	19.8 ± 6.0	0.2 ± 0.2	4770 ± 4768	550 ± 1082
2016 July	Katase (35°18′N, 139°28′E)	24	20.0 ± 2.4	0.2 ± 0.1	12044 ± 11054	2573 ± 3435
2017 July	Katase (35°18′N, 139°28′E)	9	17.9 ± 5.8	0.2 ± 0.1	14267 ± 8406	3183 ± 2773

### High-throughput sequencing

Next generation sequencing (NGS) analysis against mitochondrial cytochrome *c* oxidase subunit I (COI) showed that a sequence essentially identical to that of the flatworm *P. multitentaculata* was detected in the intestinal contents from the wild juvenile pufferfish *T. niphobles* captured from waters off Katase, Kanagawa, Japan in July 2016 (all of the three individuals analyzed in this study, Tables 2, S1 and S2) and 2017 (three of nine individuals analyzed in this study, Tables 3, S3 and S4). This sequence constituted 0.04–10.2% (all of three individuals), and 0.5–1.8% (three of nine individuals), of all the orthologous sequences from other TTX-bearing and non-toxic organisms found in the gut contents of *T. niphobles*, in 2016 and 2017, respectively. The nucleotide sequences of the partial COI gene obtained from the intestinal tract of *T. niphobles* are included in supplementary file “NGS_seq(2016).docx” and “NGS_seq(2017).docx”, and OTU ID are represented in Tables S2 and S4.

**Table 2:** DNA sequences from the intestinal contents of the pufferfish *Takifugu niphobles* juveniles collected at Katase in July 2016.

Organism	Acc. No.	Sequence identity (%)	Number of sequences from:		
			Pufferfish S1	Pufferfish S2	Pufferfish S3
TTX-bearing organisms					
Flatworm *Planocera multitentaculata*	LC190986	99	9873	70	35
Newt *Cynops pyrrhogaster*	EU880313	90	54	0	0
Goby *Yongeichthys criniger*	KT894736	99	0	3	6
Pufferfish *Chelonodon patoca*	KU692427	100	3	0	2
Non-toxic organisms^a^					
Annelida, Oligochaeta			408	79	65
Annelida, Polychaeta			171	392	1553
Arthropoda, Arachnida			2875	0	565
Arthropoda, Crustacea			81406	11104	21260
Arthropoda, Crustacea, Amphipoda			6	19416	68362
Arthropoda, Crustacea, Copepoda			853	70016	280
Arthropoda, Diplopoda			0	0	1
Arthropoda, Insecta			359	347	681
Bacillariophyta, Thalassiosirales			251	0	0
Mollusca, Bivalvia			0	5	0
Mollusca, Gastropoda			918	240	6
Platyhelminthes, Polycladida			0	0	47
Unidentified sequence			161	0	1
Total number of sequences			97338	101672	92864
TTX amount (μg/individual)			4.6	7.5	15.5

The pufferfish juveniles were randomly selected from the specimens collected at Katase in July 2016, shown in Table 1. ^a^The list of sequences from non-toxic organisms are shown in the supplementary data (Table S1).

**Table 3:** DNA sequences from the intestinal contents of the pufferfish *Takifugu niphobles* juveniles collected at Katase in July 2017.

Organism	Acc. No.	Sequence identity (%)	Number of sequence from pufferfish individual:								
			No. 1	No. 2	No. 3	No. 4	No. 5	No. 6	No. 7	No. 8	No. 9
TTX-bearing organisms											
Flatworm *Planocera multitentaculata*	LC190986	100	0	0	0	936	0	272	833	0	0
Ribbonworm *Cephalothrix simula*	GU726607	86	0	0	0	0	0	2	0	0	0
Sea snail *Rapana venosa*	KP976378	100	0	0	0	101	0	0	2	0	0
Non-toxic organisms^a^											
Annelida, Oligochaeta			2965	0	3503	5330	6434	16106	27615	14824	7891
Annelida, Polychaeta			0	2922	908	605	447	1992	2422	916	1408
Arthropoda, Chelicerata			0	0	33	0	0	86	0	0	0
Arthropoda, Crustacea			18026	17521	34623	47809	51895	35203	12205	31368	19178
Arthropoda, Crustacea, Copepoda			15506	791	5828	50	3	710	134	1445	21038
Arthropoda, Insecta			2070	0	835	373	132	0	263	178	644
Mollusca, Bivalvia			0	0	783	1536	2115	1418	126	39	268
Mollusca, Gastropoda			3204	0	0	317	69	367	3150	23	0
Nematoda, Ascaridida			0	4359	0	0	0	0	0	0	0
Platyhelminthes, Polycladida			0	0	38	0	0	2	0	0	0
Platyhelminthes, Strigeidida			0	164	0	0	0	0	0	0	0
Platyhelminthes, Tricladida			0	0	0	202	0	9	0	0	0
Vertebrata, Teleostei			0	105	0	67	18	1027	22	0	0
Chlorophyta, Microthamniales			0	0	0	22	0	0	0	0	0
Fungi, Ascomycota			0	0	0	34	0	0	0	0	0
Fungi, Microbotryomycetes			0	479	0	0	0	0	0	0	0
Unidentified sequence			594	1834	251	252	112	2	11	5	648
Total number of sequences			42365	28175	46802	57634	61225	57196	46783	48798	51075
TTX amount (μg/individual)			2.8	2.9	10	2.7	3.7	4.3	5.8	2.8	1.6

The pufferfish juveniles were randomly selected from the specimens collected at Katase in July 2017, shown in Table 1. ^a^The list of sequences from non-toxic organisms are shown in the supplementary data (Table S3).

### Planocerid-specific PCR and detection of planocerids from pufferfish gut contents

**Figure 1:**
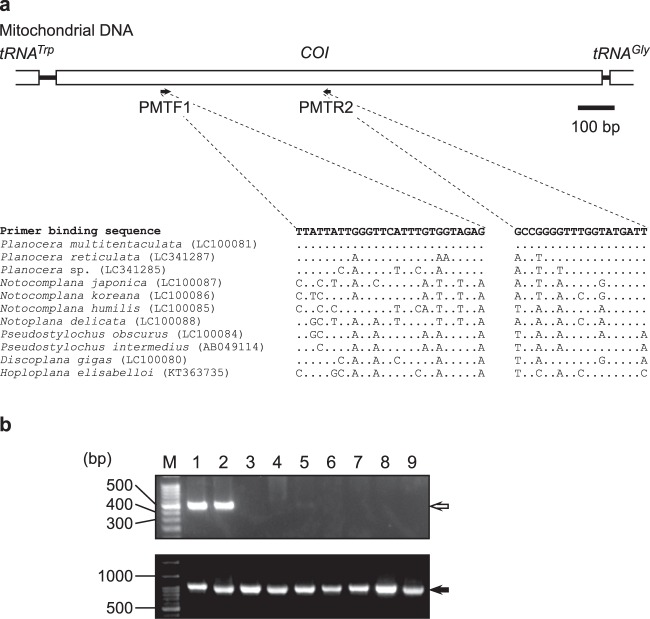
Location of primers in the 28S rRNA gene from *Planocera multitentaculata* and various flatworm species. (**a**) A schematic of the 28S rRNA gene of *P. multitentaculata* and various flatworm species, and locations of oligonucleotides used as primers for PCR and DNA sequencing. Arrows represent the location and direction of primers. (**b**) Electrophoretic pattern of PCR products from *P. multitentaculata* and various flatworm species. White arrow indicates PCR products specific to *P. multitentaculata*, and black arrow indicates those common to various flatworm species. Lane M, molecular weight marker; lanes 1 and 2, *P. multitentaculata*; lane 3, *Planocera reticulata*; lane 4, *Planocera* sp.; lane 5, *Paraplanocera oligoglena*; lane 6, *Callioplana marginata*; lane7, *Discoplana gigas*; lane 8, *Pseudostylochus obscurus*; lane 9, *Notocomplana humilis*. Full-length gels are included in a Supplementary Information file (Fig. S3).

A PCR-based method with *P. multitentaculata*-specific primers was developed for detection of the flatworm from the intestinal contents of pufferfish: 28S ribosomal RNA (rRNA) gene amplified by PCR with universal primers from all flatworm samples, whereas DNA fragments encoding the mitochondrial COI gene were detected only in *P. multitentaculata*, with length 429 bp (Fig. 1). No fragment was observed in the related species, *P. reticulata*.

**Figure 2:**
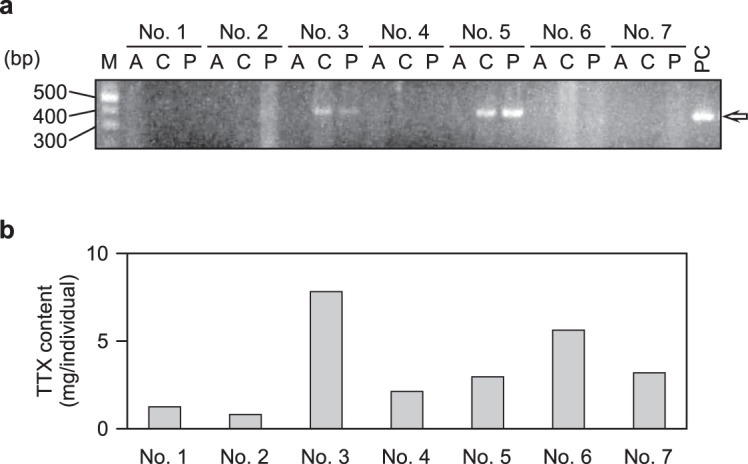
Detection of *Planocera multitentaculata* in the intestinal contents of young wild *Takifugu niphobles* individuals (**a**) and TTX content (mg/individuals) in the pufferfish individuals (**b**). The arrow indicates PCR products specific to *P. multitentaculata*. Lane M: molecular weight marker; lane A: anterior part of the intestine; lane C: middle part of the intestine; lane P, posterior part of the intestine; lane PC, positive control (*P. multitentaculata*). *P. multitentaculata*-specific band is present in No. 3 and 5 individuals. Full-length gel is included in a Supplementary Information file (Fig. S4).

PCR with *P. multitentaculata*-specific primers amplified the 429 bp long mtDNA fragment from the intestinal content of the pufferfish *T. niphobles* young, two days after being fed with *P. multitentaculata* adults in the aquarium. Similarly, a *P. multitentaculata*-specific band was observed from the intestinal contents from the adult pufferfish captured off Hayama, in July, 2016 (Fig. 2).

### Toxicity of the planocerid flatworm

**Figure 3:**
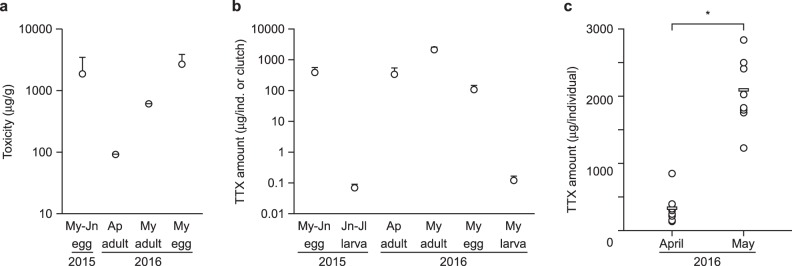
Toxicity and TTX content of the flatworm *Planocera multitentaculata* used in this study. Panels (a and b) show the toxicity and TTX content in the flatworm adult, larva and egg, respectively, while panel (c) shows a significant difference in the TTX amount between the flatworm adult collected in April and May 2016. Larvae hatched from the wild eggs were collected in laboratory aquaria. Data of larva/adult and eggs were obtained from individuals and a clutch, respectively. Bars represent means + standard deviation. Student’s *t*-test was employed for statistical comparison (*P < 0.05 in panel c).

The amount of TTX in the planocerid flatworm *P. multitentaculata* (n = 9; body weight, 3.49 ± 0.51 g) captured in April 2016 was 91.9 ± 0.9 μg/g (334.0 ± 201.9 μg/individual), corresponding to 418 ± 4 MU/g (1518 ± 918 MU/individual), whereas those captured in May, 2016 (n = 9; body weight, 3.47 ± 0.49 g) were 610.8 ± 2.1 μg/g (2091.4 ± 469.3 μg/individual), corresponding to 2776 ± 10 MU/g (9506 ± 2133 MU/individual). A statistically significant difference was observed between these two sample groups (*P* < 0.05, Fig. 3, Table S5).

The amount of TTX in the planocerid eggs obtained in 2015 was 1867 ± 1589 µg/g, corresponding to 8486 ± 7223 MU/g, whereas those obtained in 2016 contained 2673 ± 1214 µg/g, corresponding to 12150 ± 5518 MU/g (Fig. 3, Table S5). The amount of TTX in the planocerid larvae in 2015 and 2016 was 69 ± 21 ng/individual, corresponding to 0.314 ± 0.095 MU/individual, and 120 ± 46 ng/individual, corresponding to 0.545 ± 0.209 MU/individual, respectively (Fig. 3, Table S5).

### Toxification of pufferfish juveniles fed on flatworm larvae

In the predation experiments, the pufferfish juveniles fed heavily on planocerid larvae and lost equilibrium in approximately 20 min, but then recovered. Multiple reaction monitoring (MRM) patterns showed that the peak in the liquid chromatography with tandem mass spectrometry (LC-MS/MS) analysis corresponding to TTX was detected in the planocerid fed-pufferfish juveniles (Fig. S1). The amount of TTX in the whole body of the pufferfish juveniles (18, 39 and 51 dph; days post hatch) was calculated to be 407 ± 102 to 1064 ± 123 ng/individual (19.5 ± 5.4 to 54.8 ± 17.1 μg/g) (Table 4). The peak corresponding to TTX was not detected in the cultured juveniles (18 and 39 dph) of the pufferfish *T. niphobles*, which fed on non-toxic feeds (Fig. S1).

**Table 4:** Toxification of the juvenile pufferfish after feeding on the flatworm larvae.

Pufferfish (year)	No. of individuals	Total length (mm)	Body weight (g)	TTX content in flatworm larvae (μg)^a^	TTX concentration in pufferfish (μg/g)^b^	TTX amount of pufferfish (ng/ind.)^b^	Ingestion rate (%)
Flatworm-fed individual							
18 dph (2016)	28 (3 replicates)	6.1 ± 0.2	0.006 ± 0.002	3.7	37.2 ± 21.7	116 ± 19	2.9 ± 0.5
39 dph (2015)	26 (3 replicates)	7.6 ± 0.8	0.008 ± 0.001	12.1	54.8 ± 17.1	407 ± 102	3.4 ± 0.8
51 dph (2015)	30 (3 replicates)	11.1 ± 1.6	0.022 ± 0.007	15.7	19.5 ± 5.4	474 ± 241	3.0 ± 1.5
Non-toxic feed-fed individual							
18 dph (2016)	9 (1 replicate)	6.0 ± 1.4	0.006	N/A^c^	N/A	N/D^d^	N/A
39 dph (2015)	28 (3 replicates)	8.0 ± 1.2	0.009 ± 0.002	N/A	N/A	N/D	N/A

^a^TTX content in the flatworm larvae fed by a juvenile of the pufferfish was calculated. ^b^Pufferfish TTX values represent means of three independent replicate experiments. ^c^N/A: not applicable. ^d^N/D: not detected.

### Toxification of pufferfish young fed on adult flatworms

**Figure 4:**
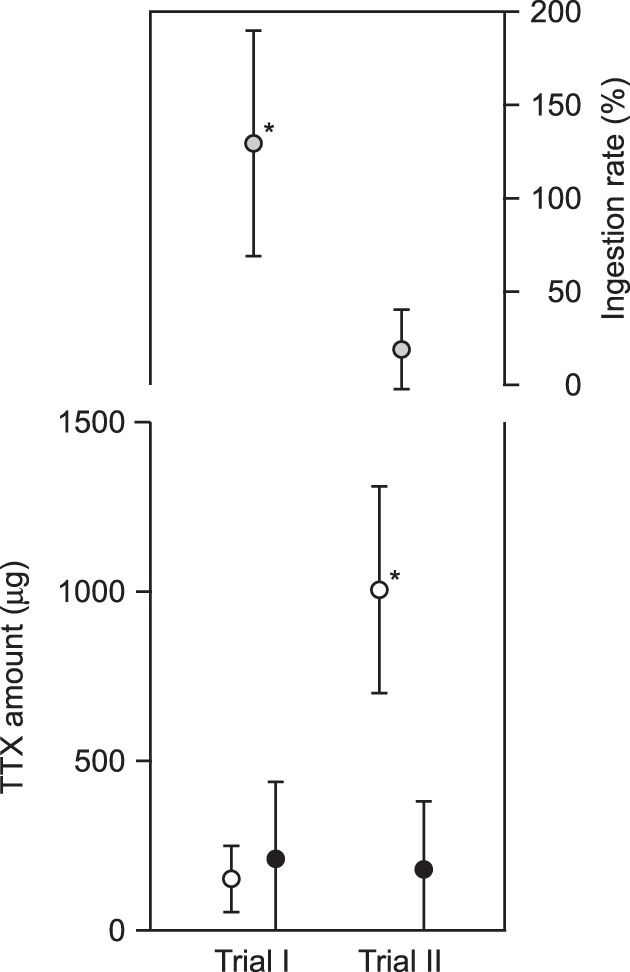
Toxification of the young pufferfish *Takifugu niphobles* after feeding on the adult flatworm *Planocera multitentaculata*. Closed circles refer to TTX accumulation in the pufferfish after feeding on half of one flatworm, while the open circles refer to the accumulation in half of one flatworm. Non-toxic pufferfish were used as a predators, and the toxic flatworms were used as prey. The flatworms collected in April and May were subjected to trial I and trial II of the toxification experiment (see text), respectively. Gray circle refers to the rate of accumulation of TTX in the pufferfish after feeding on the flatworm. Values are the mean of nine independent replicate experiments. Bars represent means ± standard deviation. Student’s *t*-test was employed for statistical comparison (*P < 0.05).

In these predation experiments, the pufferfish young fed on half of an adult flatworm and appeared to become toxified. MRM patterns showed that the peak in the LC-MS/MS analysis corresponding to TTX was detected in several tissues including intestine, liver and skin of the pufferfish (Fig. S1). The amount of TTX in the body of the toxified pufferfish young (12 months old), fed with a weakly toxic planocerid (152 ± 97 μg) was calculated to be 212 ± 227 (52–819) μg/individual, which corresponds to 964 ± 1032 MU/individual, demonstrating that almost all the TTX (129 ± 60%) in the flatworm was ingested by the pufferfish young (Fig. 4, Table S6). Similarly, the amount of TTX in the whole body of the toxified pufferfish young with a strongly toxic planocerid (1005 ± 305 μg) was calculated to be 181 ± 200 (9–543) μg/individual, which corresponds to 823 ± 909 MU/individual, demonstrating that some of the TTX (19 ± 21%) in the flatworm was ingested by the pufferfish young (Fig. 4, Table S6). TTX in the toxified pufferfish was localized in the liver (59.2 ± 12.7 to 62.0 ± 16.4%), intestine (14.7 ± 7.9 to 16.3 ± 13.9%) and skin (14.0 ± 11.2 to 22.1 ± 15.3%) (Table S7). MRM patterns showed that the peak in the LC-MS/MS analysis corresponding to TTX was not detected in any tissues from the cultured young (12 months old) *T. niphobles* that were raised on non-toxic feed (Fig. S1).

## Discussion

**Figure 5:**
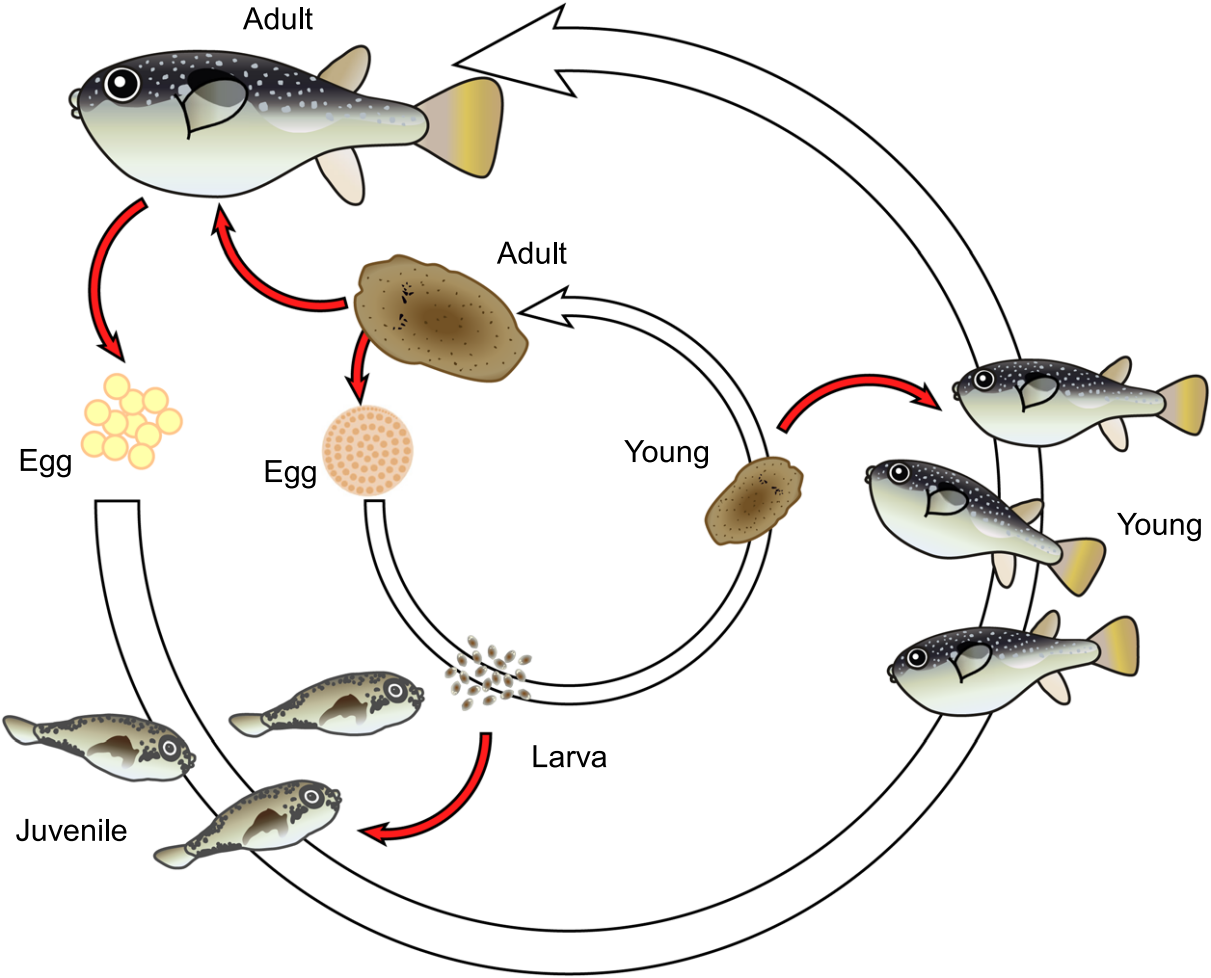
Role of the toxic flatworm *Planocera multitentaculata* in the toxification of the pufferfish *Takifugu niphobles* throughout its life cycle. Red arrows indicate transfer of TTX.

TTX in the offspring of toxic organisms such as pufferfish, octopus, newt and flatworm, appear to be obtained by means of a vertical maternal transfer^34–39^. This maternally provided TTX provides even just-hatched larvae protection from predators^34,35^. However, the protection provided by the maternal TTX, at least in pufferfish, decreases with age in the absence of further influx of TTX^25,34,40^. The larvae and juveniles of pufferfish need to obtain TTX from food in order to continue to protect themselves from predators. Effective toxification of the larval and juvenile pufferfish appears to depend on their feeding on plankton that is laden with highly concentrated TTX. TTX-bearing organisms have been identified from various taxa, including ribbonworms and flatworms, which have microplanktonic stages in their life histories^13,14,17,36^. Our study shows that the planktonic larvae of the flatworm *P. multitentaculata* contain highly concentrated TTX (69–120 ng/larva), suggesting that larval stages of the flatworm could serve not merely as suitable food but also a source for the toxification of the pufferfish larvae/juveniles. Considering that toxic flatworm sequences were detected from intestinal contents of wild juveniles and young of the pufferfish *T. niphobles*, our results suggest that the flatworm contributes to the toxification of the pufferfish *T. niphobles* throughout its life (Fig. 5).

Kono *et al*.^41^ reported that dietary administrated TTX in the pufferfish *T. niphobles* was firstly accumulated into the liver, and then gradually transferred to the skin approximately 200 days after end of TTX administration: TTX content in the skin increased two-fold, while decreasing by 40% in the liver. These results suggest that apart from larval pufferfish, older individuals also use TTX as a chemical defense against predators. This inference is corroborated by the Sakakura *et al*.^42^ study, which found the survival rates of the TTX-bearing pufferfish *T. rubripes* juveniles were higher than those of the non-toxic individuals in a salt-pond mesocosm. In the predation experiments in this study, non-toxic *T. niphobles* juveniles and young were rapidly and effectively toxified after feeding on larvae and adult specimens of the flatworm *P. multitentaculata*, respectively, accumulating large quantities of TTX. Thus, the potential for pufferfish to accumulate large quantities of TTX exist; however, this potential does not always appear to be realized, even in laboratory-based predation experiments. Variation in individual toxicity is known to be high in wild *Takifugu* pufferfish populations^23,43^. In the present study too, large individual differences in toxicity were observed in *T. niphobles* young after the toxification experiments, suggesting that variation in individual toxicity might be one of the risk management in the survival strategies in the *Takifugu* pufferfish because of energy consumption for TTX-bearing in their body. The detection of TTX in young pufferfish at levels higher than the levels contained in the half flatworm they were fed, suggests that the difference might be due to the potential differences in TTX recovery or TTX extraction efficiencies for different tissues.

Flatworms are known to contribute to the toxification of other animals as well; one study reported that dog neurotoxicosis occurred after consuming the side-gilled sea slug *Pleurobranchaea maculata* in coastal New Zealand^8^, and a subsequent study revealed that the sea slugs were toxified by feeding on the flatworm *Stylochoplana* sp.^9,17^. The extent of pufferfish toxicity varies not only among individuals but also by habitat^23^, which might be associated with the habitat-specific population size of the planocerid flatworms^36^. In any cases, although planocerid flatworms contribute to the toxification of organisms at higher trophic levels, further investigation is needed to reveal the source of TTX in the flatworms for a better understanding of the TTX-loop in the marine environments. Our study shows that in the study area, the population size of the flatworm *P. multitentaculata* is much larger than that of the related species, *P. reticulata*. Indeed, *P. multitentaculata* appears to make a greater contribution to the toxification of the larvae and juveniles of the pufferfish, because toxic eggs of *P. multitentaculata*, and not of *P. reticulata*, have been observed in the area during the spawning period of the pufferfish^36,37^. The TTX source of the planktonic flatworms (excluding the maternal TTX) might be key to resolving the missing link to the TTX-loop proposed by our previous study^33^.

The toxification of the TTX-bearing organisms, including pufferfish, has been thought to be achieved through the classical food webs^22,23^, although the evidence thus far has largely only been the presence of organisms with indigestible tissues such as the starfish *Astropecten polyacanthus*, identified from the gut-contents of TTX-bearing organisms, such as a trumpet shell *Charonia sauliae*^44^. There has been little evidence of the toxification of other toxic organisms by organisms without indigestible tissues except for the flatworm *Stylochoplana* sp., which is considered responsible for the toxification of the grey side-gilled sea slug *Pleurobranchaea maculata*^9,17^. In our study, in order to determine if wild *T. niphobles* fed on the flatworm, *P. multitentaculata*, we developed PCR methods specific to the flatworm based on results from NGS analysis against a generic animal COI gene. Our results suggest that some wild pufferfish feed on the flatworm in the inshore waters around Hayama, Japan, and that future large scale metagenomics analyses of the intestinal contents of TTX-bearing organisms might reveal the mechanism of toxification.

In conclusion, we have shown, by means of TTX quantification, predation experiments, and tools of molecular biology, that the pufferfish *T. niphobles* can be toxified simply by feeding on the flatworm *P. multitentaculata*. The TTX content of the flatworm increased in association with increasing body weight^36^, and flatworms carrying TTX were classified into planocerid and the related species^16^. These reports and our results would contribute to the elucidation of the pufferfish toxification mechanism.

## Materials and Methods

### Pufferfish and flatworm

Wild (toxic) pufferfish *Takifugu niphobles* juveniles (15–34 mm total length, 0.10–0.67 g body weight) were captured in August 2010, August 2011 and July 2015 from coastal waters of Oiso, Japan (35°18′N, 139°19′E) and in July 2016 and July 2017 from coastal waters of Katase, Japan (35°18′N, 139°28′E). Non-toxic juveniles and young were raised from artificially fertilized eggs obtained from wild mature females and males captured in the summer (May–July) of 2015 and 2016 at Enoshima Island, Japan (35°18′N, 139°28′E), and subsequently grown with non-toxic feed (rotifer and artemia) and commercial food pellets in the aquarium (Fig. S1). Wild adult and young specimens were captured during June–July of 2016, and June–July of 2017 off the coast of Hayama, Japan (35°15′N, 139°34′E). Adult specimens of the flatworm *Planocera multitentaculata* were captured during April–July of 2015 and 2016 also at the coast of Hayama, Japan, while (toxic) flatworm larvae hatched from eggs that were spawned in the laboratory aquaria, by wild parents derived from Hayama.

### LC-MS/MS analysis

TTX was extracted from samples with 0.1% acetic acid, the extract was filtered through a membrane of pore size 0.45-μm (SupraPure Syringe Filter, PTEE-Hydrophilic, Recenttec, Taipei, Taiwan) and subjected to analysis using a LC-MS/MS, following Itoi *et al*.^43^. Quantification was done using a Quattro Premier XE mass spectrometer (Waters, Milford, MA, USA) equipped with an electrospray ionization (ESI) source coupled to an Acquity UPLC system (Waters), following Itoi *et al*.^33^. Chromatographic separation was done using an Atlantis HILIC Silica column (2.1 mm × 150 mm, 5 μm; Waters), coupled to an Atlantis HILIC Silica pre-column (2.1 mm × 10 mm, 5 μm; Waters), with gradient elution of formic acid/acetonitrile. The mass spectrometer was operated in MRM, detecting in positive mode, analyzing two product ions at *m*/*z* 162 for quantification of TTX and *m*/*z* 302 for confirmation of the compound from the precursor ion at *m*/*z* 320. The calibration curve was generated with 1 to 100 ng/ml of TTX standard (Wako Pure Chemicals, Osaka, Japan), which showed good linearity and precision (*y* = 105.164*x* + 15.610, *r*^2^ = 0.9975). Quantification of TTX was carried out using the data for samples with >1000-fold dilution to remove any matrix effect, since it was recovered from the samples with >1000-fold dilution in accordance with our previous studies^33,36^. The limit of detection (LOD) of the measurement system was determined based on signal to noise ratio (S/N = 3). The LOD value was calculated at 0.059 ng/ml for TTX. One MU is equivalent to 0.22 μg of TTX, based on the specific toxicity of TTX^45^.

### High-throughput sequencing

Genomic DNA in the intestinal contents of the juvenile pufferfish *T. niphobles* was extracted with Fast DNA spin kit for Soil (MO Bio Laboratories, Illkirch, France) based on the manufacturer’s instructions. Fragments of COI gene (approximately 500 bp) were amplified from the extracted genomic DNA by PCR using universal COI primers: 1^st^-IntF (5′-Seq A-GCTCT TCCCA TCTGT GCCAG CMGCC GCGGT AA-3′) and 1^st^-HCOmR (5′-Seq B-CTCTT CCGAT CTTAH ACTTC NGGGT GKCCR AARAA TCA-3′), where Seq A (5′-ACACT CTTTC CCTAC ACGAC-3′) and Seq B (5′-GTGAC TGGAG TTCAG ACGTG TG-3′) represent nucleotide sequences targeted by the second PCR primers. A blocking primer (5′-TTACC CACCC CTAGC AGGAA ATCTT GCCCACGCAG G-Spacer C3-3′) was used in the amplicon PCR, to prevent amplification from the pufferfish *T. niphobles*. A Spacer C3 CpG in the 3′ end of the blocking primer was added to prevent elongation without affecting annealing properties, and minimizing predator DNA amplification. PCR amplification was done under the following conditions: an initial denaturation at 94 °C for 2 min followed by 30 cycles of denaturation at 94 °C for 30 sec, annealing at 67 °C for 15 sec and 52 °C for 30 sec, and extension at 72 °C for 30 sec, with a final extension step at 72 °C for 5 min. PCR products were amplified again using additional forward primer (5′-Adaptor C-Tag sequence-Seq A-3′) and reverse primer (5′-Adaptor D-Seq B-3′), where Adaptors C and D were used for the MiSeq sequencing reaction. The Tag sequence included 8 nucleotides designed for sample identification barcoding. Thermal cycling was done under the following conditions: an initial denaturation at 94 °C for 2 min followed by 12 cycles of denaturation at 94 °C for 30 sec, annealing at 60 °C for 30 sec, and extension at 72 °C for 30 sec, with a final extension step at 72 °C for 5 min. PCR amplicons from each sample were used for high-throughput sequencing on a MiSeq Genome Sequencer (Illumina, CA, USA). The sequences obtained for each sample were grouped based on tag sequences, and average read length of 320 bp was obtained. Negative controls (reactions with no template) were prepared for all steps of the process after DNA extraction to check for contamination. Before analyzing the food organisms, we removed sequences if they met any of the following criteria: <40 bp in length, with a phred-equivalent quality score of <20, containing ambiguous characters, with an uncorrected barcode, or missing the primer sequence. The identities of the phylotypes were analyzed by comparing the sequences against the DDBJ/EMBL/GenBank databases using a BLAST search^46^.

### DNA extraction and PCR amplification

Small tissue samples from adult specimens of the flatworm *P. multitentaculata*, and intestinal contents from wild specimens of the pufferfish *T. niphobles* were collected. Total genomic DNA was extracted from the flatworm tissues and the intestinal contents of the pufferfish using the method of Noguchi *et al*.^47^ with some modification. Briefly, proteinase K-treated samples were subjected to phenol/chloroform extraction with MaXtract High Density (Qiagen, Germantown, MD, USA). Partial fragments of 28S rRNA gene were amplified by PCR using primers HRNT-F2 (5′-AGTTC AAGAG TACGT GAAAC C-3′) and HRNT-R2 (5′-AACAC CTTTT GTGGT ATCTG ATGA-3′), which were designed with universal primers for the 28S rRNA gene (approx. 1,000 bp) of various polyclads^48^, whereas those of COI gene were amplified by PCR using *P. multitentaculata*-specific primers PMTF1 (5′-TTATT ATTGG GTTCA TTTGT GGTAG AG-3′) and PMTR2 (5′-AATCA TACCA AACCC CGGC-3′), which were designed based on the sequences of the COI gene (429 bp) from *P. multitentaculata* and other polyclads (Fig. 1). PCR amplification was done in a 20 μl reaction mixture containing genomic DNA as a template, 1 unit *ExTaq* DNA polymerase (Takara Bio, Shiga, Japan), 1.6 μl of 2.5 mM deoxynucleotide triphosphates (dNTP), 5 μl of 5 μM primers, and 2 μl of 10× *ExTaq* DNA polymerase buffer (Takara Bio). The thermal cycling program for the PCR consisted of an initial denaturation at 95 °C for 1 min followed by 35 cycles of denaturation at 95 °C for 10 s, annealing at 55 °C for 30 s and extension at 72 °C for 45 s.

### Direct sequencing

Prior to sequencing the amplified product, the DNA fragment was purified by chloroform extraction, followed by polyethylene glycol (PEG) 8000 precipitation and ethanol precipitation. Both strands were sequenced using a 3130*xl* genetic analyzer (Applied Biosystems, Foster, CA, USA) and a BigDye Terminator v3.1 Cycle Sequencing Ready Reaction Kit (Applied Biosystems). The nucleotide sequences of the amplified products were aligned using Clustal Omega^49^ with those in the DDBJ/EMBL/GenBank databases obtained using a BLAST search^46^.

### Toxification experiment

#### Pufferfish juveniles vs. planocerid larvae

The toxification experiment was carried out using non-toxic juveniles (within 2 months old) of the pufferfish *T. niphobles* (8–10 individuals) as the predator and toxic planocerid larvae (2000–3000 larvae) as prey, in a 500-ml beaker. The treatment was repeated three times, except for the control sample in 2016. In the toxification experiment, the non-toxic juveniles of the pufferfish *T. niphobles* (standard length: 7.2–12.4 mm; body weight: 0.06–0.30 g) fed on the toxic planocerid larvae. After more than two days of feeding, the pufferfish juveniles (8–10 individuals pooled in a sample) and non-toxic control (8–10 individuals pooled in a sample) were subjected to the TTX extraction process followed by LC-MS/MS analysis.

#### Pufferfish young vs. planocerid adults

The toxification experiment was also carried out in a 50 L glass aquarium using non-toxic young (12 months old) of the pufferfish *T. niphobles* as the predator and adult flatworm *P. multitentaculata* as the prey. Since no significant difference in the TTX distribution was observed in both halves of adult flatworm individual (Fig. S2), half the body of a flatworm adult was subjected to LC-MS/MS analysis, and the remaining half fed to a non-toxic pufferfish young, which were kept in a 50 L glass aquaria with a circulating filtration system. The treatment was repeated nine times. After more than two days of feeding on the adult planocerid, the pufferfish young were subjected to TTX extraction followed by LC-MS/MS analysis.

### Statistical analysis

The statistical significance of differences in the amount of toxin was analyzed by means of a student’s *t*-test. Data are given as mean ± standard deviation.

### Ethical statement

All animal procedures comply with the Japanese Government Animal Protection and Management Law (No. 105) and Japanese Government Notification on Feeding and Safekeeping of Animals (No. 6).

## Electronic supplementary material

Supplemenatry data

NGS_seq(2016).docx

NGS_seq(2017).docx
