# Supplementary material for: Including planocerid flatworms in the diet effectively toxifies the pufferfish, Takifugu niphobles
Source: Sci Rep. 2018 Aug 17;8:12302. doi: 10.1038/s41598-018-30696-z (PMC6098040; doi:10.1038/s41598-018-30696-z)
Supplement: Supplementary file 1 — Supplemenatry data [file 41598_2018_30696_MOESM1_ESM.pdf]

Supplementary Information

Including planocerid flatworms in the diet effectively toxifies the pufferfish, *Takifugu niphobles*

Shiro Itoi[1], Hiroyuki Ueda[1], Riko Yamada[1], Mitsuki Takei[1], Tatsunori Sato[1], Shotaro Oshikiri[1], Yoshiki Wajima[1], Ryuya Ogata[1], Hikaru Oyama[1], Takahiro Shitto[1], Kazuya Okuhara[1], Tadasuke Tsunashima[1], Eitaro Sawayama[2], Haruo Sugita[1]

[1] *Department of Marine Science and Resources, Nihon University, Fujisawa, Kanagawa 252-0880, Japan*

[2] *R&D Division, Marua Suisan Co., Ltd. Kamijima, Ehime 794-2410, Japan*

Correspondence and requests for materials should be addressed to S.I. (e-mail: sitoi@nihon-u.ac.jp)

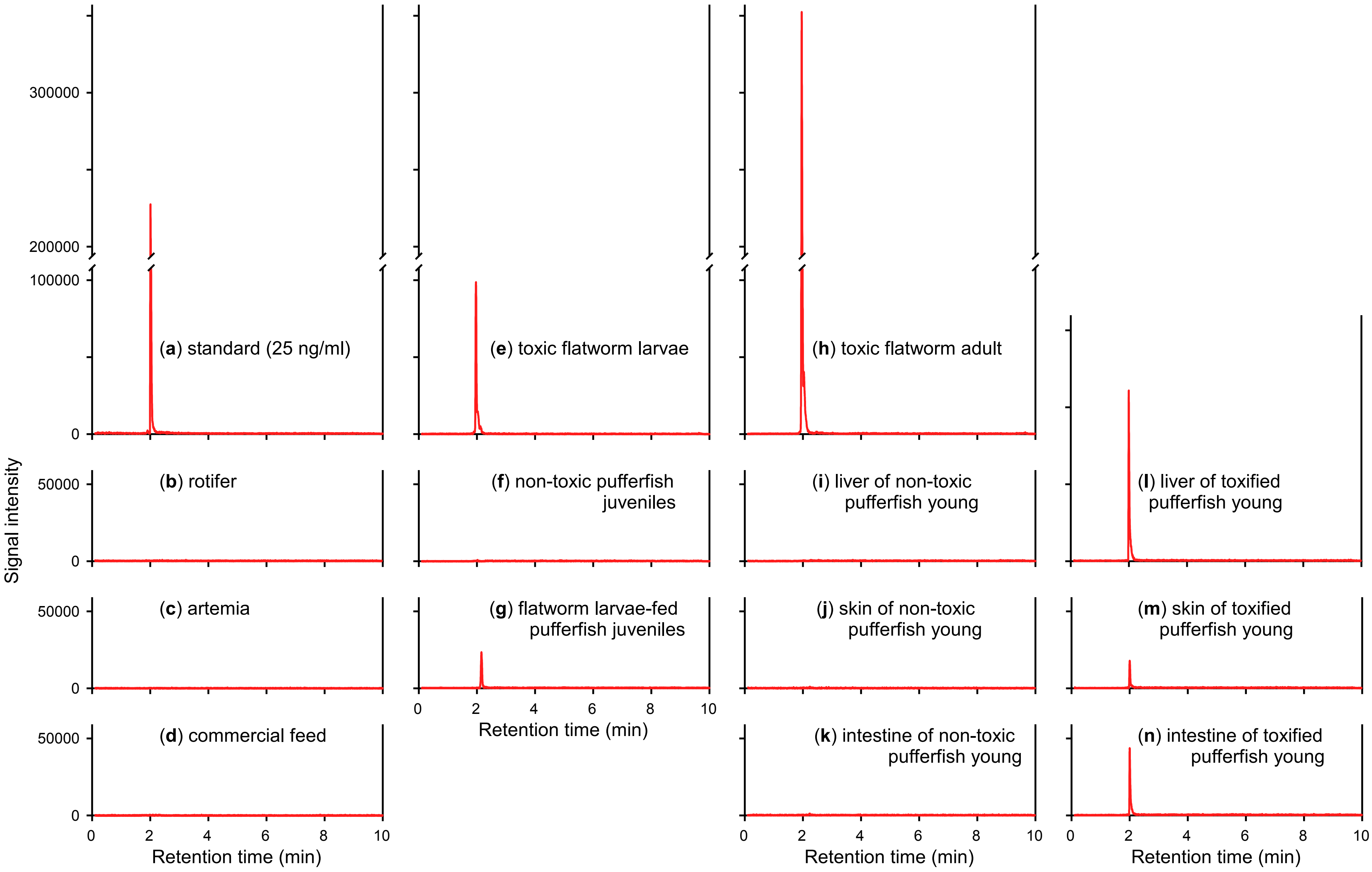

Fig. S1. Typical mass chromatograms of the LC-MS/MS obtained under MRM mode (m/z 320 > 302). MRM patterns of 25 ng/ml TTX standard (**a**), the extract from the rotifer (**b**), the extract from the artemia (**c**), the extract from the commercial feed (**d**), the extract from the toxic flatworm larvae (**e**), the extract from the non-toxic *T. niphobles* juveniles cultured with only commercial feed (**f**), the extract from pufferfish juvenile fed with the toxic flatworm larvae (**g**), the extract from the toxic flatworm adult (**h**), the extract from the non-toxic *T. niphobles* young cultured with only commercial feed (**i**, liver; **j**, skin; **k**, intestine), and the extract from the toxic flatworm adult fed-pufferfish young (**l**, liver; **m**, skin; **n**, intestine).

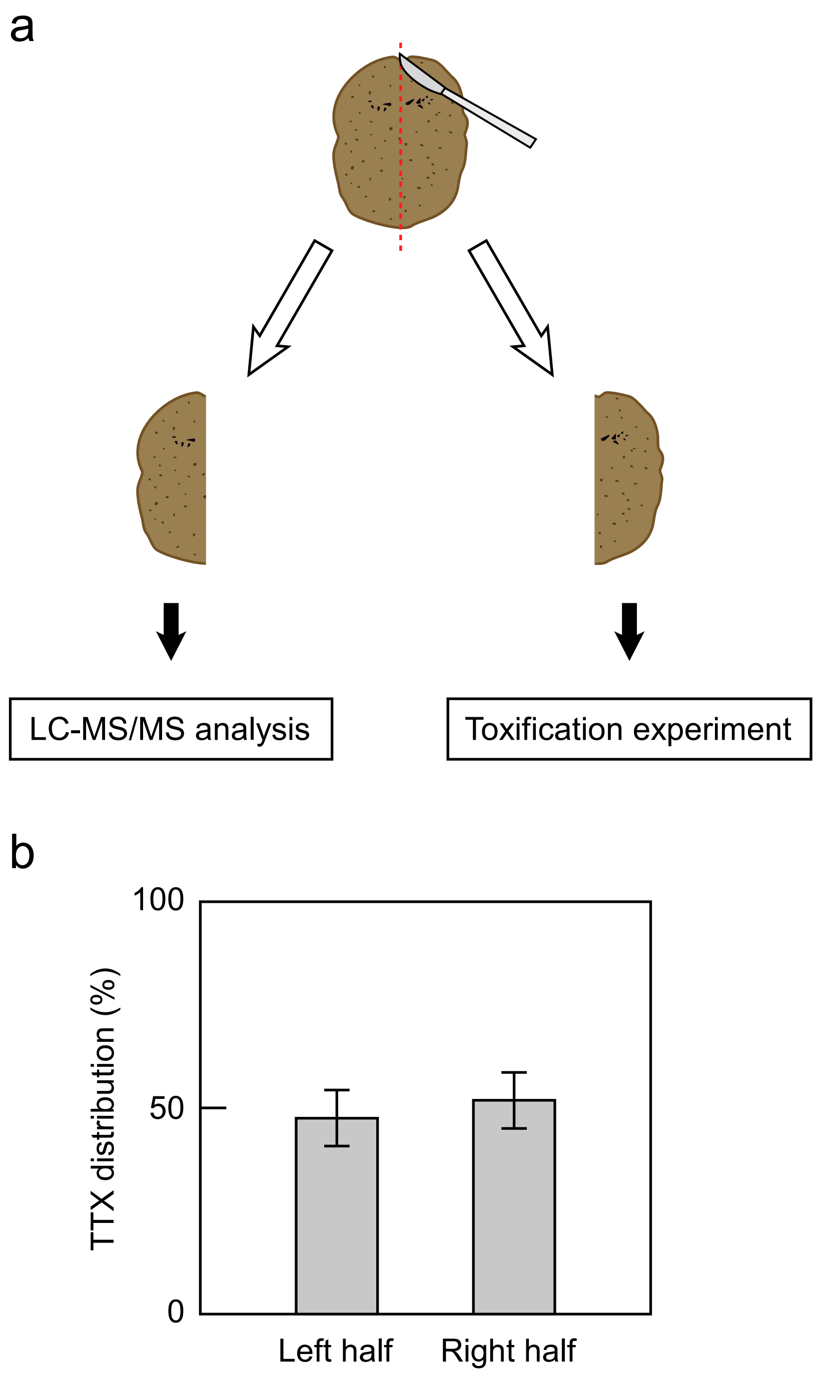

Fig. S2. How to cut the flatworm in half (**a**) and the toxin levels of left and right halves (**b**). In the preliminary experiment, the TTX contents in both halves of individual adults were measured (n = 9). After confirmation that the toxin levels were equivalent in each half, half the body was subjected to LC-MS/MS analysis, and the remaining half was used for the predation experiments. Bars represent means ± standard deviation.

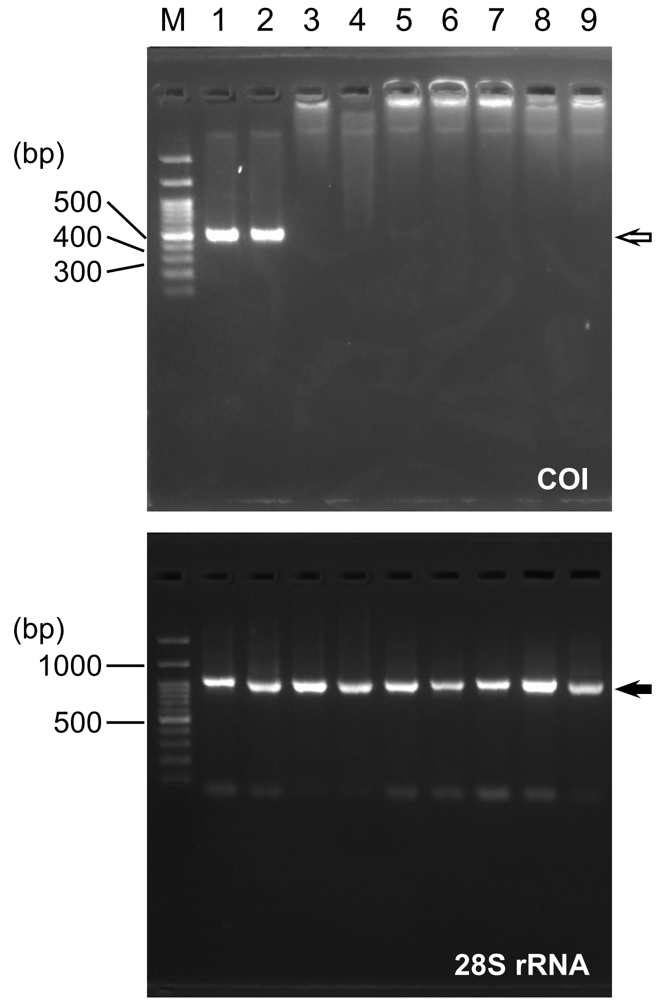

Fig. S3. Full-length gel electrophoretic pattern of PCR products from *Planocera multitentaculata* and various flatworm species shown in Fig. 1. White arrow indicates PCR products specific to *P. multitentaculata*, and black arrow indicates those common to various flatworm species. Lane M, molecular weight marker; lanes 1 and 2, *P. multitentaculata*; lane 3, *Planocera reticulata*; lane 4, *Planocera* sp.; lane 5, *Paraplanocera oligoglena*; lane 6, *Callioplana marginata*; lane7, *Discoplana gigas*; lane 8, *Pseudostylochus obscurus*; lane 9, *Notocomplana humilis*.

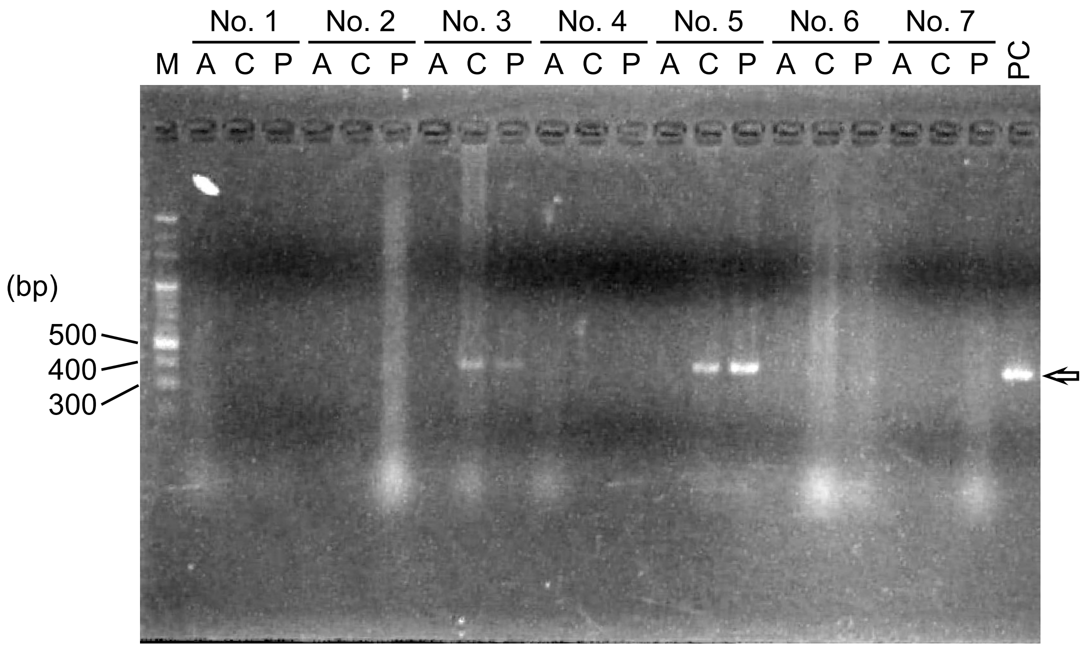

Fig. S4. Full-length gel electrophoretic pattern of PCR products from *Planocera multitentaculata* in the intestinal contents of seven young wild *Takifugu niphobles* individuals shown in Fig. 2. The arrow indicates PCR products specific to *P. multitentaculata*. Lane M: molecular weight marker; lane A: anterior part of the intestine; lane C: middle part of the intestine; lane P, posterior part of the intestine; lane PC, positive control (*P. multitentaculata*).

Table S1. DNA sequences from the intestinal contents of the pufferfish *Takifugu niphobles* juveniles collected at Katase in July 2016 [1]

Organism	Classification	Accession No.	Sequence identity (%)	Number of sequences from pufferfish ind.: No. 1	No. 2	No. 3
TTX-bearing organisms						
Planocera multitentaculata	Platyhelminthes, Polycladida	LC190986	99	9873	70	35
Cynops pyrrhogaster	Vertebrata, Amphibia	EU880313	90	54	0	0
Yongeichthys criniger	Vertebrata, Teleostei	KT894736	99	0	3	6
Chelonodon patoca	Vertebrata, Teleostei	KU692427	100	3	0	2
Non-toxic organisms						
Amynthas tappensis	Annelida, Oligochaeta	AB542551	92	99	4	1
Lumbricus rubellus	Annelida, Oligochaeta	KX790443	79	309	75	64
Boccardiella hamata	Annelida, Polychaeta	KP231329	80	0	0	1005
Polydora cornuta	Annelida, Polychaeta	AB636160	78	171	392	548
Antrodiaetus pacificus	Arthropoda, Arachnida	KP654824	81-83	2875	0	565
Amphibalanus amphitrite	Arthropoda, Crustacea	JQ035516	100	0	0	12
Amphibalanus eburneus	Arthropoda, Crustacea	KU695280	97-99	501	128	172
Balanus trigonus	Arthropoda, Crustacea	JQ035523	99	991	17	0
Chthamalus challengeri	Arthropoda, Crustacea	KM594046	99	64	32	82
Penilia avirostris	Arthropoda, Crustacea	KP136698	100	79850	10927	20994
Ampithoe valida	Arthropoda, Crustacea, Amphipoda	JX545458	100	0	0	183
Polynesoecetes kekeae	Arthropoda, Crustacea, Amphipoda	KC706702	82	0	0	286
Aoroides columbiae	Arthropoda, Crustacea, Amphipoda	JX545451	83	0	1348	5961
Ericthonius punctatus	Arthropoda, Crustacea, Amphipoda	KT209486	83	3	1028	42
Jassa marmorata	Arthropoda, Crustacea, Amphipoda	KT601632	88	0	64	0
Grandidierella japonica	Arthropoda, Crustacea, Amphipoda	JX545464	100	2	9732	60301
Melita nitida	Arthropoda, Crustacea, Amphipoda	KF273565	99	0	0	158
Monocorophium insidiosum	Arthropoda, Crustacea, Amphipoda	KT209034	86-98	1	7244	1431
Caligus curtus	Arthropoda, Crustacea, Copepoda	KT209407	79	0	138	0
Pseudodiaptomus ishigakiensis	Arthropoda, Crustacea, Copepoda	AB576158	98	14	26121	29
Paracalanus parvus	Arthropoda, Crustacea, Copepoda	EU599545	97-100	386	467	12
Pseudevadne tergestina	Arthropoda, Crustacea, Copepoda	EU675911	100	422	0	159
Pseudodiaptomus inopinus	Arthropoda, Crustacea, Copepoda	JQ714055	97	31	19544	0
Pseudodiaptomus koreanus	Arthropoda, Crustacea, Copepoda	JQ714054	96-97	0	23677	55
Sabelliphilidae sp.	Arthropoda, Crustacea, Copepoda	KT030284	74	0	69	25
Oxidus gracilis	Arthropoda, Diplopoda	KU875705	91	0	0	1
Amphinemura borealis	Arthropoda, Insecta	KY262067	84	5	0	0
Chathamia integripennis	Arthropoda, Insecta	KX038240	82	13	70	0
Procladius paludicola	Arthropoda, Insecta	HQ248009	84	0	0	365
Colletes compactus	Arthropoda, Insecta	FJ582200	79	0	0	6
Phyllotreta hemipoda	Arthropoda, Insecta	KX943496	75	42	0	0
Pseudonapomyza lacteipennis	Arthropoda, Insecta	KR678719	84	19	0	0
Asteromyia carbonifera	Arthropoda, Insecta	JX417020	83	280	0	309
Drosophila suzukii	Arthropoda, Insecta	MG605095	78	0	277	0
Chersodromia incana	Arthropoda, Insecta	KJ082677	74	0	0	1
Cyclotella sp.	Bacillariophyta, Thalassiosirales	KM202117	90	72	0	0
Skeletonema costatum	Bacillariophyta, Thalassiosirales	LC222536	89-90	179	0	0
Crassostrea gigas	Mollusca, Bivalvia	KX436133	100	0	5	0
Nassarius festivus	Mollusca, Gastropoda	JQ975446	98	105	0	0
Tritonoharpa angasi	Mollusca, Gastropoda	FR717522	89	221	0	6
Pyrgulopsis micrococcus	Mollusca, Gastropoda	AY367469	79	58	233	0
Reishia clavigera	Mollusca, Gastropoda	KP116315	100	534	7	0
Platyhelminthes sp.	Platyhelminthes, Polycladida	KP254635	81	0	0	47
Unidentified sequences				161	0	1
Total number of sequences				97338	101672	92864

[1] The pufferfish juveniles were randomly selected from the specimens collected at Katase in July 2016, represented in Table 1. DNA sequences are included in supplementary file “NGS_seq(2016).docx”. OTU IDs are represented in Table S2.

Table S2. Number of DNA sequence (OTU ID) from the intestinal contents of the pufferfish *Takifugu niphobles* juveniles collected at Katase in July 2016 [1]

Organism [2]	OTU ID	Number of sequences from:		
		Pufferfish 1	Pufferfish 2	Pufferfish 3
TTX-bearing organisms				
Planocera multitentaculata	OTU_2	9873	70	35
Cynops pyrrhogaster	OTU_12	54	0	0
Yongeichthys criniger	OTU_1	0	3	6
Chelonodon patoca	OTU_3	3	0	2
Non-toxic organisms				
Amynthas tappensis	OTU_4	99	4	1
Lumbricus rubellus	OTU_72	309	75	64
Boccardiella hamata	OTU_42	0	0	1005
Polydora cornuta	OTU_44	171	392	548
Antrodiaetus pacificus	OTU_32	2875	0	565
Amphibalanus amphitrite	OTU_266	0	0	12
Amphibalanus eburneus	OTU_51 and 245	501	128	172
Balanus trigonus	OTU_48	991	17	0
Chthamalus challengeri	OTU_130	64	32	82
Penilia avirostris	OTU_6	79850	10927	20994
Ampithoe valida	OTU_77	0	0	183
Polynesoecetes kekeae	OTU_70	0	0	286
Aoroides columbiae	OTU_25	0	1348	5961
Ericthonius punctatus	OTU_45	3	1028	42
Jassa marmorata	OTU_143	0	64	0
Grandidierella japonica	OTU_9 and 11	2	9732	60301
Melita nitida	OTU_79	0	0	158
Monocorophium insidiosum	OTU_15, 53, 64 and 91	1	7244	1431
Caligus curtus	OTU_86	0	138	0
Pseudodiaptomus ishigakiensis	OTU_24	14	26121	29
Paracalanus parvus	OTU_67 and 348	386	467	12
Pseudevadne tergestina	OTU_83	422	0	159
Pseudodiaptomus inopinus	OTU_18 and 355	31	19544	0
Pseudodiaptomus koreanus	OTU_16 and 35	0	23677	55
Sabelliphilidae sp.	OTU_102	0	69	25
Oxidus gracilis	OTU_13	0	0	1
Amphinemura borealis	OTU_10	5	0	0
Chathamia integripennis	OTU_101 and 111	13	70	0
Procladius paludicola	OTU_59	0	0	365
Colletes compactus	OTU_307	0	0	6
Phyllotreta hemipoda	OTU_149	42	0	0
Pseudonapomyza lacteipennis	OTU_248	19	0	0
Asteromyia carbonifera	OTU_55	280	0	309
Drosophila suzukii	OTU_65	0	277	0
Chersodromia incana	OTU_19	0	0	1
Cyclotella sp.	OTU_116	72	0	0
Skeletonema costatum	OTU_114 and 145	179	0	0
Crassostrea gigas	OTU_420	0	5	0
Nassarius festivus	OTU_106	105	0	0
Tritonoharpa angasi	OTU_76	221	0	6
Pyrgulopsis micrococcus	OTU_80	58	233	0
Reishia clavigera	OTU_8	534	7	0
Platyhelminthes sp.	OTU_125	0	0	47
Unidentified sequences	OTU_20, 144, 168, 197, 211 and 254	161	0	1
Total number of sequences		97338	101672	92864

[1] The pufferfish juveniles were randomly selected from the specimens collected at Katase in July 2016, represented in Table 1.

[2] Organisms named as presented in Table S1.

Table S3. DNA sequences from the intestinal contents of the pufferfish *Takifugu niphobles* juveniles collected at Katase in July 2017 [1]

Organism	Classification	Acc. No.	Sequence identity (%)	Number of sequences from pufferfish individual:								
				No. 1	No. 2	No. 3	No. 4	No. 5	No. 6	No. 7	No. 8	No. 9
TTX-bearing organisms												
Planocera multitentaculata	Platyhelminthes, Polycladida	LC190986	100	0	0	0	936	0	272	833	0	0
Cephalothrix simula	Nemertea, Cephalothrix	GU726607	86	0	0	0	0	0	2	0	0	0
Rapana venosa	Mollusca, Gastropoda	KP976378	100	0	0	0	101	0	0	2	0	0
Non-toxic organisms												
Eophila gestroi	Annelida, Oligochaeta	KT352938	81	0	0	120	2051	767	3964	9672	0	0
Fitzingeria platyura	Annelida, Oligochaeta	KX651141	83	0	0	0	167	15	256	783	0	0
Lumbricidae sp.	Annelida, Oligochaeta	GU014023	80	1	0	80	161	340	967	1581	2285	2773
Lumbricus rubellus	Annelida, Oligochaeta	KX790443	79	2964	0	3303	2951	5312	10777	15506	12539	5118
Metaphire sieboldi	Annelida, Oligochaeta	AB482099	81	0	0	0	0	0	115	0	0	0
Travoscolides chengannures	Annelida, Oligochaeta	KX832079	80	0	0	0	0	0	0	73	0	0
Tubificoides diazi	Annelida, Oligochaeta	KP254080	80	0	0	0	0	0	27	0	0	0
Enipo sp.	Annelida, Polychaeta	HQ023871	82	0	0	0	0	0	0	256	0	0
Eumida alkyone	Annelida, Polychaeta	HM358691	91	0	0	0	0	0	15	0	0	0
Eumida longicornuta	Annelida, Polychaeta	JQ623499	88	0	0	0	0	34	0	25	0	538
Gastrolepidia clavigera	Annelida, Polychaeta	KC706796	81	0	0	0	0	3	0	4	0	0
Harmothoe fuligineum	Annelida, Polychaeta	KX867415	86	0	0	0	0	52	0	0	0	0
Lumbrineris sp.	Annelida, Polychaeta	KR916858	75	0	0	0	0	0	54	0	0	0
Malacoceros fuliginosus	Annelida, Polychaeta	EF432012	85	0	0	0	0	0	0	152	20	0
Marenzelleria arctia	Annelida, Polychaeta	EF137728	86	0	0	0	0	0	34	21	138	0
Mediomastus sp.	Annelida, Polychaeta	EU835672	79	0	0	0	0	20	146	2	2	0
Micropodarke dubia	Annelida, Polychaeta	JN571825	100	0	0	0	71	0	0	0	0	0
Nephtys hombergii	Annelida, Polychaeta	KR916886	82	0	0	0	0	0	28	2	0	0
Notophyllum crypticum	Annelida, Polychaeta	GQ464342	85	0	0	0	0	30	0	0	0	0
Paraprionospio patiens	Annelida, Polychaeta	KU661499	95	0	0	17	0	0	3	0	0	0
Polydora cornuta	Annelida, Polychaeta	AB636160	78-79	0	0	877	314	101	148	1343	543	136
Polydora haswelli	Annelida, Polychaeta	KP231333	96-100	0	0	0	0	0	0	201	105	0
Polydora hoplura	Annelida, Polychaeta	KY677864	85-89	0	0	0	35	19	511	156	105	0
Polydora websteri	Annelida, Polychaeta	KR337462	81-99	0	0	0	185	14	29	184	3	734
Sabellaria sp.	Annelida, Polychaeta	KX525585	88	0	2922	14	0	174	240	28	0	0
Scolelepis daphoinos	Annelida, Polychaeta	GU362687	88	0	0	0	0	0	0	48	0	0
Spiochaetopterus sp.	Annelida, Polychaeta	KX525515	83	0	0	0	0	0	784	0	0	0
Eupodidae sp.	Arthropoda, Chelicerata	JX838561	79	0	0	33	0	0	0	0	0	0
Sericosura curva	Arthropoda, Chelicerata	KP835344	77	0	0	0	0	0	86	0	0	0
Amphibalanus amphitrite	Arthropoda, Crustacea	JQ035516	99	0	0	3	1	1	0	14	6	192
Amphibalanus eburneus	Arthropoda, Crustacea	KU695280	98-99	0	0	110	0	10	50	30	0	97
Amphibalanus improvisus	Arthropoda, Crustacea	KT209079	100	507	0	180	5	2439	189	104	34	300
Amphipoda sp.	Arthropoda, Crustacea	GQ260850	84	0	0	0	0	0	0	0	40	0
Arcotheres sinensis	Arthropoda, Crustacea	JX502950	99	0	0	247	0	0	0	0	1130	0
Austrochiltonia sp.	Arthropoda, Crustacea	JX900273	83	0	0	0	0	0	0	0	11	0
Balanus trigonus	Arthropoda, Crustacea	JQ035523	100	2324	0	1932	15	0	655	343	373	7142
Caridea sp.	Arthropoda, Crustacea	HM466029	76	0	0	88	0	0	0	0	0	0
Chthamalus cf. *challengeri*	Arthropoda, Crustacea	AY823019	100	0	0	0	23926	21498	18083	1955	0	0

Chthamalus proteus	Arthropoda, Crustacea	AY822966	83	0	0	135	0	0	0	0	0	0
Corophium multisetosum	Arthropoda, Crustacea	KX224017	83	0	0	0	0	0	182	0	0	0
Cryptopodia angulata	Arthropoda, Crustacea	KF241718	84	0	0	0	88	0	0	0	0	0
Evadne spinifera	Arthropoda, Crustacea	KT208687	98	0	0	0	0	0	4	5	0	0
Fistulobalanus albicostatus	Arthropoda, Crustacea	KU977407	100	0	0	369	23258	23294	4564	226	17	0
Gaetice depressus	Arthropoda, Crustacea	HM180589	100	0	0	0	0	0	49	0	0	0
Grandidierella japonica	Arthropoda, Crustacea	JX545464	100	0	0	0	0	0	0	227	453	0
Hemigrapsus sanguineus	Arthropoda, Crustacea	KT307116	100	0	0	0	0	0	22	0	0	0
Hemigrapsus takanoi	Arthropoda, Crustacea	KT208924	100	0	0	0	0	0	0	0	2004	0
Hyperacanthomysis longirostris	Arthropoda, Crustacea	HM045290	85	0	0	0	0	0	0	240	0	0
Isopoda sp.	Arthropoda, Crustacea	HM180631	96	0	0	0	43	0	0	0	0	0
Leptodius nigromaculatus	Arthropoda, Crustacea	HM751003	100	0	0	677	0	0	196	1236	116	2544
Metapenaeopsis mogiensis	Arthropoda, Crustacea	KR150459	99	0	0	0	0	0	179	0	0	0
Monocorophium insidiosum	Arthropoda, Crustacea	KT209034	86-100	0	0	3183	0	2	0	0	589	0
Pachygrapsus crassipes	Arthropoda, Crustacea	AY952139	99	0	0	607	47	17	1075	143	23265	0
Penilia avirostris	Arthropoda, Crustacea	HM045341	99	0	0	0	0	0	1	0	11	0
Penilia avirostris	Arthropoda, Crustacea	KP136698	100	2173	17521	675	229	4394	7148	2125	3047	2433
Pleopis polyphemoides	Arthropoda, Crustacea	EU675896	100	0	0	0	0	0	13	0	0	0
Plesionika quasigrandis	Arthropoda, Crustacea	KM096448	79	0	0	0	0	0	0	4	0	0
Pseudevadne tergestina	Arthropoda, Crustacea	EU675911	100	0	0	0	0	102	1537	5070	0	0
Sesarma reticulatum	Arthropoda, Crustacea	EU329170	92	12981	0	26417	197	94	1204	483	272	6470
Tetraclita japonica	Arthropoda, Crustacea	DQ647753	99	0	0	0	0	21	0	0	0	0
Thalestris longimana	Arthropoda, Crustacea	KT208624	77	41	0	0	0	0	0	0	0	0
Trevathana dongshaensis	Arthropoda, Crustacea	KY439840	91	0	0	0	0	23	52	0	0	0
Acartia tsuensis	Arthropoda, Crustacea, Copepoda	KC287425	98	1718	0	48	0	0	0	4	198	230
Clausocalanus furcatus	Arthropoda, Crustacea, Copepoda	KC287559	100	0	0	0	0	0	45	0	0	0
Cyclops kikuchii	Arthropoda, Crustacea, Copepoda	KR048967	100	0	791	0	0	0	0	0	0	98
Labidocera japonica	Arthropoda, Crustacea, Copepoda	JQ714069	97	0	0	0	0	0	17	0	0	0
Longipedia kikuchii	Arthropoda, Crustacea, Copepoda	KR049012	90-99	1887	0	3678	0	0	0	0	58	12611
Mastigodiaptomus albuquerquensis	Arthropoda, Crustacea, Copepoda	KC617098	79	0	0	0	0	0	0	0	0	36
Mospicalanus sp.	Arthropoda, Crustacea, Copepoda	KU247786	82	0	0	63	0	0	0	0	0	0
Oithona dissimilis	Arthropoda, Crustacea, Copepoda	AB604159	77	241	0	0	0	0	0	0	0	0
Paracalanus aculeatus	Arthropoda, Crustacea, Copepoda	KC287803	96	0	0	0	0	3	5	0	0	0
Paracalanus parvus	Arthropoda, Crustacea, Copepoda	EU856803	100	0	0	307	46	0	623	112	0	235
Paracalanus tropicus	Arthropoda, Crustacea, Copepoda	KF715939	97	0	0	0	0	0	0	6	0	0
Paracyclopina nana	Arthropoda, Crustacea, Copepoda	EU877959	77	0	0	0	0	0	0	0	0	136
Pseudodiaptomus inopinus	Arthropoda, Crustacea, Copepoda	JQ714055	97	4734	0	295	0	0	0	0	14	4817
Pseudodiaptomus ishigakiensis	Arthropoda, Crustacea, Copepoda	AB576158	97	0	0	215	4	0	20	12	17	808
Pseudodiaptomus koreanus	Arthropoda, Crustacea, Copepoda	JQ714054	96-98	6926	0	1139	0	0	0	0	991	2067
Pseudodiaptomus marinus	Arthropoda, Crustacea, Copepoda	KT209405	100	0	0	83	0	0	0	0	167	0
Bradysia hilariformis	Arthropoda, Insecta	JQ613827	80	0	0	649	0	0	0	0	0	0
Chaetocladius perennis	Arthropoda, Insecta	KR756465	82	0	0	0	0	0	0	16	0	0
Chazara briseis	Arthropoda, Insecta	FJ663347	79	0	0	0	54	46	0	105	0	0
Corynoneura sp.	Arthropoda, Insecta	JF286782	85	0	0	0	0	0	0	131	0	0
Diptera sp.	Arthropoda, Insecta	KP421238	78	0	0	0	0	65	0	0	0	0
Heliconius sara	Arthropoda, Insecta	KP848964	82	0	0	0	0	0	0	11	0	0

Leuctra grandis	Arthropoda, Insecta	JN200609	74	0	0	0	0	0	0	0	0	482
Lysimelia neleusalis	Arthropoda, Insecta	JN401276	82	0	0	0	0	0	0	0	0	55
Orthocladiinae sp.	Arthropoda, Insecta	KR757067	80	0	0	0	304	0	0	0	0	0
Orthocladiinae sp.	Arthropoda, Insecta	KR591648	77	160	0	138	0	0	0	0	1	107
Pintomyia fischeri	Arthropoda, Insecta	AB984434	78	1910	0	0	0	0	0	0	0	0
Polypedilum cultellatum	Arthropoda, Insecta	AB838682	100	0	0	0	0	20	0	0	0	0
Probergrothius sexpunctatus	Arthropoda, Insecta	KX523454	79	0	0	0	0	0	0	0	153	0
Pseudonapomyza lacteipennis	Arthropoda, Insecta	KR678719	84	0	0	0	15	1	0	0	0	0
Smittia nudipennis	Arthropoda, Insecta	HQ105350	75	0	0	48	0	0	0	0	0	0
Spialia orbifer	Arthropoda, Insecta	KU905500	80	0	0	0	0	0	0	0	24	0
Brachidontes pharaonis	Mollusca, Bivalvia	AJ865698	84	0	0	164	0	0	0	0	0	0
Crassostrea gigas	Mollusca, Bivalvia	KT988318	100	0	0	409	1445	1903	1199	125	30	0
Dendostrea crenulifera	Mollusca, Bivalvia	KC683510	98	0	0	0	66	204	33	0	0	0
Ostrea circumpicta	Mollusca, Bivalvia	KP067904	100	0	0	0	0	6	0	0	0	53
Ostrea stentina	Mollusca, Bivalvia	LC051589	100	0	0	89	0	0	33	0	0	0
Pseudopythina subsinuata	Mollusca, Bivalvia	AB714897	87	0	0	0	0	0	106	0	0	0
Xenostrobus atratus	Mollusca, Bivalvia	AB298598	98	0	0	121	25	2	47	1	9	215
Blasicrura kieneri	Mollusca, Gastropoda	DQ324049	99	0	0	0	0	0	3	1	0	0
Conomurex luhuanus	Mollusca, Gastropoda	JF693431	100	0	0	0	0	18	0	1	0	0
Crepidula onyx	Mollusca, Gastropoda	AF546025	95	0	0	0	0	0	0	7	0	0
Lacuna pallidula	Mollusca, Gastropoda	KT996151	86	0	0	0	1	0	0	19	10	0
Nassarius sp.	Mollusca, Gastropoda	KY451382	90	0	0	0	0	0	5	0	0	0
Peasiella habei	Mollusca, Gastropoda	HE590847	99	0	0	0	30	0	0	0	0	0
Philine babai	Mollusca, Gastropoda	KF877703	85	0	0	0	39	0	0	0	0	0
Pterygia dactylus	Mollusca, Gastropoda	KR087291	82	0	0	0	0	0	349	0	0	0
Pyrgulopsis micrococcus	Mollusca, Gastropoda	AY367470	79	3204	0	0	0	0	0	0	0	0
Reishia bronni	Mollusca, Gastropoda	HQ852758	99	0	0	0	18	0	1	2	0	0
Reishia clavigera	Mollusca, Gastropoda	KP116909	100	0	0	0	229	44	9	12	13	0
Stenomelania crenulata	Mollusca, Gastropoda	AB920321	83	0	0	0	0	0	0	3108	0	0
Tritonoharpa antiquata	Mollusca, Gastropoda	FR717519	90	0	0	0	0	7	0	0	0	0
Baylisascaris schroederi	Nematoda, Ascaridida	KJ587827	88	0	4359	0	0	0	0	0	0	0
Pseudobiceros bedfordi	Platyhelminthes, Polycladida	KY421515	74	0	0	38	0	0	2	0	0	0
Apatemon sp.	Platyhelminthes, Strigeidida	KT334182	84	0	164	0	0	0	0	0	0	0
Obama ladislavii	Platyhelminthes, Tricladida	KC608258	78	0	0	0	202	0	9	0	0	0
Decapterus maruadsi	Vertebrata, Teleostei	KY570761	100	0	0	0	67	18	0	0	0	0
Larimichthys crocea	Vertebrata, Teleostei	LT972191	89	0	105	0	0	0	0	0	0	0
Omobranchus punctatus	Vertebrata, Teleostei	KY315359	100	0	0	0	0	0	970	1	0	0
Prochilodus nigricans	Vertebrata, Teleostei	FJ418758	86	0	0	0	0	0	0	21	0	0
Sphyraena pinguis	Vertebrata, Teleostei	HM180889	99	0	0	0	0	0	57	0	0	0
Trebouxia aggregata	Chlorophyta, Microthamniales	EU123948	87	0	0	0	22	0	0	0	0	0
Tetracladium breve	Fungi, Ascomycota	EU883410	86	0	0	0	34	0	0	0	0	0
Rhodotorula mucilaginosa	Fungi, Microbotryomycetes	MF694646	100	0	479	0	0	0	0	0	0	0
Unidentified sequences				594	1834	251	252	112	2	11	5	648
Total number of sequences				42365	28175	46802	57634	61225	57196	46783	48798	51075

[1] The pufferfish juveniles were randomly selected from the specimens collected at Katase in July 2017, represented in Table 1. DNA sequences are included in supplementary file "NGS_seq(2017).docx". OTU IDs are represented in Table S4.

Table S4. Number of DNA sequence (OTU ID) from the intestinal contents of the pufferfish *Takifugu niphobles* juveniles collected at Katase in July 2017 [1]

Organism	OTU ID	Number of sequences from pufferfish individual:								
		No. 1	No. 2	No. 3	No. 4	No. 5	No. 6	No. 7	No. 8	No. 9
TTX-bearing organisms										
Planocera multitentaculata	OTU_27	0	0	0	936	0	272	833	0	0
Cephalothrix simula	OTU_155	0	0	0	0	0	2	0	0	0
Rapana venosa	OTU_67	0	0	0	101	0	0	2	0	0
Non-toxic organisms [2]										
Eophila gestroi	OTU_12	0	0	120	2051	767	3964	9672	0	0
Fitzingeria platyura	OTU_138	0	0	0	167	15	256	783	0	0
Lumbricidae sp.	OTU_17	1	0	80	161	340	967	1581	2285	2773
Lumbricus rubellus	OTU_2	2964	0	3303	2951	5312	10777	15506	12539	5118
Metaphire sieboldi	OTU_68	0	0	0	0	0	115	0	0	0
Travoscolides chengannures	OTU_70	0	0	0	0	0	0	73	0	0
Tubificoides diazi	OTU_118	0	0	0	0	0	27	0	0	0
Enipo sp.	OTU_41	0	0	0	0	0	0	256	0	0
Eumida alkyone	OTU_139	0	0	0	0	0	15	0	0	0
Eumida longicornuta	OTU_35	0	0	0	0	34	0	25	0	538
Gastrolepidia clavigera	OTU_133	0	0	0	0	3	0	4	0	0
Harmothoe fuligineum	OTU_88	0	0	0	0	52	0	0	0	0
Lumbrineris sp.	OTU_99	0	0	0	0	0	54	0	0	0
Malacoceros fuliginosus	OTU_51	0	0	0	0	0	0	152	20	0
Marenzelleria arctia	OTU_60	0	0	0	0	0	34	21	138	0
Mediomastus sp.	OTU_101	0	0	0	0	20	146	2	2	0
Micropodarke dubia	OTU_116	0	0	0	71	0	0	0	0	0
Nephtys hombergii	OTU_115	0	0	0	0	0	28	2	0	0
Notophyllum crypticum	OTU_117	0	0	0	0	30	0	0	0	0
Paraprionospio patiens	OTU_123	0	0	17	0	0	3	0	0	0
Polydora cornuta	OTU_23, 46 and 141	0	0	877	314	101	148	1343	543	136
Polydora haswelli	OTU_47, 72 and 150	0	0	0	0	0	0	201	105	0
Polydora hoplura	OTU_44, 53 and 110	0	0	0	35	19	511	156	105	0
Polydora websteri	OTU_31, 48 and 158	0	0	0	185	14	29	184	3	734
Sabellaria sp.	OTU_22	0	2922	14	0	174	240	28	0	0
Scolelepis daphoinos	OTU_103	0	0	0	0	0	0	48	0	0
Spiochaetopterus sp.	OTU_39	0	0	0	0	0	784	0	0	0
Eupodidae sp.	OTU_104	0	0	33	0	0	0	0	0	0
Sericosura curva	OTU_98	0	0	0	0	0	86	0	0	0
Amphibalanus amphitrite	OTU_75	0	0	3	1	1	0	14	6	192
Amphibalanus eburneus	OTU_79 and 157	0	0	110	0	10	50	30	0	97
Amphibalanus improvisus	OTU_21	507	0	180	5	2439	189	104	34	300
Amphipoda sp.	OTU_95	0	0	0	0	0	0	0	40	0
Arcotheres sinensis	OTU_30	0	0	247	0	0	0	0	1130	0
Austrochiltonia sp.	OTU_129	0	0	0	0	0	0	0	11	0
Balanus trigonus	OTU_18	2324	0	1932	15	0	655	343	373	7142
Caridea sp.	OTU_81	0	0	88	0	0	0	0	0	0
Chthamalus cf. *challengeri*	OTU_6	0	0	0	23926	21498	18083	1955	0	0

Chthamalus proteus	OTU_69	0	0	135	0	0	0	0	0	0
Corophium multisetosum	OTU_56	0	0	0	0	0	182	0	0	0
Cryptopodia angulata	OTU_80	0	0	0	88	0	0	0	0	0
Evadne spinifera	OTU_140	0	0	0	0	0	4	5	0	0
Fistulobalanus albicostatus	OTU_5	0	0	369	23258	23294	4564	226	17	0
Gaetice depressus	OTU_100	0	0	0	0	0	49	0	0	0
Grandidierella japonica	OTU_29	0	0	0	0	0	0	227	453	0
Hemigrapsus sanguineus	OTU_109	0	0	0	0	0	22	0	0	0
Hemigrapsus takanoi	OTU_25	0	0	0	0	0	0	0	2004	0
Hyperacanthomysis longirostris	OTU_49	0	0	0	0	0	0	240	0	0
Isopoda sp.	OTU_113	0	0	0	43	0	0	0	0	0
Leptodius nigromaculatus	OTU_11	0	0	677	0	0	196	1236	116	2544
Metapenaeopsis mogiensis	OTU_57	0	0	0	0	0	179	0	0	0
Monocorophium insidiosum	OTU_20, 38 and 96	0	0	3183	0	2	0	0	589	0
Pachygrapsus crassipes	OTU_4	0	0	607	47	17	1075	143	23265	0
Penilia avirostris	OTU_154	0	0	0	0	0	1	0	11	0
Penilia avirostris	OTU_3	2173	17521	675	229	4394	7148	2125	3047	2433
Pleopis polyphemoides	OTU_126	0	0	0	0	0	13	0	0	0
Plesionika quasigrandis	OTU_153	0	0	0	0	0	0	4	0	0
Pseudevadne tergestina	OTU_10	0	0	0	0	102	1537	5070	0	0
Sesarma reticulatum	OTU_1	12981	0	26417	197	94	1204	483	272	6470
Tetraclita japonica	OTU_114	0	0	0	0	21	0	0	0	0
Thalestris longimana	OTU_106	41	0	0	0	0	0	0	0	0
Trevathana dongshaensis	OTU_93	0	0	0	0	23	52	0	0	0
Acartia tsuensis	OTU_28	1718	0	48	0	0	0	4	198	230
Clausocalanus furcatus	OTU_86	0	0	0	0	0	45	0	0	0
Cyclops kikuchii	OTU_34	0	791	0	0	0	0	0	0	98
Labidocera japonica	OTU_149	0	0	0	0	0	17	0	0	0
Longipedia kikuchii	OTU_9, 24 and 85	1887	0	3678	0	0	0	0	58	12611
Mastigodiaptomus albuquerquensis	OTU_107	0	0	0	0	0	0	0	0	36
Mospicalanus sp.	OTU_84	0	0	63	0	0	0	0	0	0
Oithona dissimilis	OTU_78	241	0	0	0	0	0	0	0	0
Paracalanus aculeatus	OTU_143	0	0	0	0	3	5	0	0	0
Paracalanus parvus	OTU_33	0	0	307	46	0	623	112	0	235
Paracalanus tropicus	OTU_132	0	0	0	0	0	0	6	0	0
Paracyclopina nana	OTU_62	0	0	0	0	0	0	0	0	136
Pseudodiaptomus inopinus	OTU_13	4734	0	295	0	0	0	0	14	4817
Pseudodiaptomus ishigakiensis	OTU_61	0	0	215	4	0	20	12	17	808
Pseudodiaptomus koreanus	OTU_7 and 147	6926	0	1139	0	0	0	0	991	2067
Pseudodiaptomus marinus	OTU_77	0	0	83	0	0	0	0	167	0
Bradysia hilariformis	OTU_32	0	0	649	0	0	0	0	0	0
Chaetocladius perennis	OTU_119	0	0	0	0	0	0	16	0	0
Chazara briseis	OTU_64	0	0	0	54	46	0	105	0	0
Corynoneura sp.	OTU_55	0	0	0	0	0	0	131	0	0
Diptera sp.	OTU_90	0	0	0	0	65	0	0	0	0
Heliconius sara	OTU_135	0	0	0	0	0	0	11	0	0

Leuctra grandis	OTU_54	0	0	0	0	0	0	0	0	482
Lysimelia neleusalis	OTU_89	0	0	0	0	0	0	0	0	55
Orthocladiinae sp.	OTU_45	0	0	0	304	0	0	0	0	0
Orthocladiinae sp.	OTU_65	160	0	138	0	0	0	0	1	107
Pintomyia fischeri	OTU_26	1910	0	0	0	0	0	0	0	0
Polypedilum cultellatum	OTU_120	0	0	0	0	20	0	0	0	0
Probergrothius sexpunctatus	OTU_58	0	0	0	0	0	0	0	153	0
Pseudonapomyza lacteipennis	OTU_125	0	0	0	15	1	0	0	0	0
Smittia nudipennis	OTU_94	0	0	48	0	0	0	0	0	0
Spialia orbifer	OTU_131	0	0	0	0	0	0	0	24	0
Brachidontes pharaonis	OTU_92	0	0	164	0	0	0	0	0	0
Crassostrea gigas	OTU_15	0	0	409	1445	1903	1199	125	30	0
Dendostrea crenulifera	OTU_50	0	0	0	66	204	33	0	0	0
Ostrea circumpicta	OTU_87	0	0	0	0	6	0	0	0	53
Ostrea stentina	OTU_74	0	0	89	0	0	33	0	0	0
Pseudopythina subsinuata	OTU_71	0	0	0	0	0	106	0	0	0
Xenostrobus atratus	OTU_59	0	0	121	25	2	47	1	9	215
Blasicrura kieneri	OTU_152	0	0	0	0	0	3	1	0	0
Conomurex luhuanus	OTU_122	0	0	0	0	18	0	1	0	0
Crepidula onyx	OTU_127	0	0	0	0	0	0	7	0	0
Lacuna pallidula	OTU_112	0	0	0	1	0	0	19	10	0
Nassarius sp.	OTU_159	0	0	0	0	0	5	0	0	0
Peasiella habei	OTU_111	0	0	0	30	0	0	0	0	0
Philine babai	OTU_108	0	0	0	39	0	0	0	0	0
Pterygia dactylus	OTU_40	0	0	0	0	0	349	0	0	0
Pyrgulopsis micrococcus	OTU_19	3204	0	0	0	0	0	0	0	0
Reishia bronni	OTU_148	0	0	0	18	0	1	2	0	0
Reishia clavigera	OTU_76	0	0	0	229	44	9	12	13	0
Stenomelania crenulata	OTU_14	0	0	0	0	0	0	3108	0	0
Tritonoharpa antiquata	OTU_142	0	0	0	0	7	0	0	0	0
Baylisascaris schroederi	OTU_16	0	4359	0	0	0	0	0	0	0
Pseudobiceros bedfordi	OTU_105	0	0	38	0	0	2	0	0	0
Apatemon sp.	OTU_63	0	164	0	0	0	0	0	0	0
Obama ladislavii	OTU_52	0	0	0	202	0	9	0	0	0
Decapterus maruadsi	OTU_82	0	0	0	67	18	0	0	0	0
Larimichthys crocea	OTU_97	0	105	0	0	0	0	0	0	0
Omobranchus punctatus	OTU_36	0	0	0	0	0	970	1	0	0
Prochilodus nigricans	OTU_121	0	0	0	0	0	0	21	0	0
Sphyraena pinguis	OTU_91	0	0	0	0	0	57	0	0	0
Trebouxia aggregata	OTU_134	0	0	0	22	0	0	0	0	0
Tetracladium breve	OTU_102	0	0	0	34	0	0	0	0	0
Rhodotorula mucilaginosa	OTU_37	0	479	0	0	0	0	0	0	0
Unidentified sequences	OTU_ 42, 43, 66, 83, 124, 130, 145, 146, 151, 156, 160 and 161	594	1834	251	252	112	2	11	5	648
Total number of sequences		42365	28175	46802	57634	61225	57196	46783	48798	51075

[1] The pufferfish juveniles were randomly selected from the specimens collected at Katase in July 2017, represented in Table 1.

[2] Organisms named as presented in Table S3.

Table S5. Toxicity and TTX amount in the flatworm *Planocera multitentaculata* used in this study

Date		Stage[1]	Weight (g)[2]	No. of ind. or clutch[3]	Toxicity (μg/g)	TTX amount (μg/ind. or clutch)
2015	May-June	Egg	0.29 ± 0.10	15	1867 ± 1589	384 ± 169
	June-July	Larva	-	5700	-	0.069 ± 0.021
2016	April	Adult	3.49 ± 0.51	9	91.9 ± 0.9	334.0 ± 201.9
	May	Adult	3.47 ± 0.49	9	610.8 ± 2.1	2091.4 ± 469.3
		Egg	0.04 ± 0.02	19	2673 ± 1214	106 ± 40
		Larva	-	8500	-	0.120 ± 0.046

[1] Larvae hatched from the wild eggs were collected in the laboratory aquaria.

[2] Weight of adult represents body weight.

[3] Data of larva/adult and eggs were obtained from individuals and a clutch, respectively.

Table S6. Toxification of the young pufferfish after feeding on adult flatworms

Pufferfish	Experiment	No. of individuals	Total length (mm)	Body weight (g)	TTX amount of the flatworm (μg)[1]	TTX amount of the pufferfish (μg/ind.)[2]	Ingestion rate (%)
Flatworm-fed individual	Trial I	9	85 ± 5	10.6 ± 2.2	152 ± 97	212 ± 227	129 ± 60*
	Trial II	9	84 ± 6	10.7 ± 2.3	1,005 ± 305*	181 ± 200	19 ± 21
Non-toxic feed-fed individual	Control	6	89 ± 5	13.0 ± 1.6	N/A[3]	N/D[4]	N/A

[1] TTX amount in the toxic tissue of the flatworm was estimated based on the toxin content of half an individual.

[2] TTX amount of the pufferfish is the sum of skin, liver, gonad, intestine and others.

[3] N/A: not applicable.

[4] N/D: not detected.

* Significant difference was observed between trial I and II ($P < 0.05$).

Table S7. Tissue localization (%) of TTX in the pufferfish after feeding on toxic flatworm

Tissue	Trial I (n=9)	Trial II (n=9)
Skin	14.0 ± 11.2	22.1 ± 15.3
Liver	62.0 ± 16.4	59.2 ± 12.7
Gonad	0.9 ± 1.0	0.9 ± 0.9
Intestine	14.7 ± 7.9	16.3 ± 13.9
Others	8.4 ± 7.5	1.4 ± 2.2
