# Supplementary material for: Including planocerid flatworms in the diet effectively toxifies the pufferfish, Takifugu niphobles
Source: Sci Rep. 2018 Aug 17;8:12302. doi: 10.1038/s41598-018-30696-z (PMC6098040; doi:10.1038/s41598-018-30696-z)
Supplement: Supplementary file 2 — NGS_seq(2016).docx [file 41598_2018_30696_MOESM2_ESM.docx]

>OTU_1

ACTAGCGGGCAACCTAGCACATGCAGGAGCCTCAGTAGATCTTACCATTTTTTCCCTCCACTTGGCAGGAATTTCCTCAA

TCCTGGGGGCTATTAATTTTATTACCACAATTCTAAACATGAAGCCGCCAGCCATCTCTCAATACCAAACTCCCTTGTTT

GTATGAGCTGTTTTAATTACTGCTGTACTACTTCTCCTCTCCCTTCCAGTTCTTGCCGCTGGCATCACCATACTATTGAC

AGACCGAAACTTGAACACGACCTTTTTCGACCCCGCAGGAGGGGGGGACCCAATTCTATACCAACACCTCTTC

>OTU_2

TCTATCAGGTAATATTGCTCAGAGGGGTCCGAGAGTAGATATGGCAATATTTTCATTACATTTAGCTGGTGTTAGGTCTA

TATTGGGATCTATAAAATTCATTACTACTATGGTAAAAGCCAAGGTACAAGTTACATGAGGACAATTACCTTTGTTTTTA

TGAGCAGTAATGGTAACAGCCTATATGTTAGTATTATCATTACCTGTCTTGGCAGGAGGTCTAACAATGTTGTTAACAGA

CCGAAAATTTAACACTTCATTTTTCGATCCAGGAGGAGGAGGAGATCCCATCCTATTTCAACATATTTTT

>OTU_3

GCTAGCAGGCAACCTAGCCCACGCAGGCGCATCCGTCGACCTCACCATCTTCTCCCTCCACCTAGCAGGTGTTTCATCAA

TCCTAGGCGCTATTAACTTCATCACCACAATCATTAACATAAAACCCCCTGCTATCTCCCAGTACCAAACCCCTCTCTTC

GTATGGGCCGTTCTAATCACCGCCATCCTTCTTCTTTTATCCCTACCCGTCCTCGCAGCAGGCATTACAATGCTCCTTAC

GGACCGAAACCTAAACACCACCTTCTTCGACCCCGCAGGCGGAGGTGATCCCATCCTCTACCAGCACTTATTC

>OTU_4

TCTAGCAAGAAATATTGCTCATGCGGGCCCTTCAGTAGACCTAGCAATTTTTTCCCTTCATCTAGCTGGAGCCTCTTCAA

TCTTAGGTGCAATCAACTTTATTACCACAGTAATTAATATACGATGATCTGGTTTACGTTTAGAGCGAATTCCTCTATTT

GTGTGGGCAGTTGTAATTACTGTAGTTCTACTACTTTTATCTTTACCAGTTCTAGCTGGGGCTATCACAATACTATTAAC

AGATCGAAATCTAAATACATCATTCTTTGACCCTGCTGGAGGAGGGGATCCAATTTTATATCAACATCTATTC

>OTU_6

TTTATCAAGAACAATCGCCCACGCGGGTGCTTCTGTAGATCTTAGAATCTTCTCCTTGCATTTAGCGGGGATTTCATCAA

TCCTCGGAGCTGTAAACTTTATTACGACAATTGTAAATATACGATCTAAAGGAATAACTTTAGATCGTATTCCCCTCTTT

GTGTGGGCTGTTGGAATTACTGCTTTATTACTCCTACTTAGACTTCCTGTACTTGCAGGAGCTATCACTATGCTTCTGAC

AGACCGAAATTTAAATACTTCTTTCTTTGATCCTGCGGGAGGGGGGGACCCTATTCTTTATCAACACTTGTTC

>OTU_8

ATTGGCTGGAAATCTGGCTCATGCTGGTGGTTCAGTAGACCTTGCAATTTTTTCTTTACACTTAGCTGGGGTTTCTTCTA

TTTTAGGGGCTGTGAACTTTATTACAACCATTATTAACATACGTTGACGAGGTATACAGTTTGAACGACTTCCTCTTTTT

GTGTGATCTGTGAAAATTACAGCAATCCTTCTTCTACTATCTCTTCCAGTGCTAGCAGGGGCTATTACGATACTGTTAAC

AGATCGAAATTTTAATACTGCATTCTTTGATCCTGCAGGAGGAGGGGATCCTATTTTATATCAGCATTTATTT

>OTU_9

ATTAAGAGCAACCATCGCACACTCAGGGGGCTCAGTAGATTTAGCCATTTTTTCCCTTCATTTAGCAGGGGCCTCCTCAA

TTTTAGGAGCCATTAATTTTATCTCAACAGTCCTTAATATACGAGCCCCTGGAATAACCATAGACCGTGTTCCACTTTTT

GTGTGGTCTGTTTTTATCACTGCAATCCTCTTATTATTGTCTTTACCAGTCCTTGCAGGAGCTATTACAATATTGTTAAC

AGACCGCAACTTAAACACCTCTTTCTTTGACCCTTTAGGAGGGGGAGACCCAATTCTTTACCAACACCTATTT

>OTU_10

CCTGTCAGCCGGCATTGCCCACGCGGGTTCATCGGTTGATATGGCTATTTTTTCTCTTCATTTGGCTGGCGTTTCATCTA

TTCTAGGAGCCGTTAATTTCATTACAACCGTGATTAATATACGATCCTCAGGAATAACCCTAGATCGAATACCCCTATTT

GTTTGAGCAGTAGCTATCACAGCTCTACTTCTCTTACTGTCTCTTCCTGTATTAGCAGGTGCAATCACTATATTACTAAC

AGACCGTAATTTAAATACTTCATTCTTTGACCCTGCTGGGGGAGGGGACCCTATTTTATACCAACATTTATTT

>OTU_11

CTTAAGAGCAACCATCGCACATTCAGGAGGATCAGTAGATTTAGCCATTTTTTCTCTTCATTTAGCAGGGGCCTCGTCAA

TTTTAGGAGCTATTAATTTTATTTCAACAGTCCTTAATATACGAGCCCCTGGAATAACCATAGACCGCGTTCCGCTTTTC

GTATGATCTGTTTTTATTACTGCAATCCTCTTGTTATTGTCTTTACCGGTCCTTGCAGGAGCTATTACAATACTGCTAAC

AGACCGCAACTTAAATACATCTTTCTTCGACCCTTTAGGAGGAGGAGATCCAATTCTTTACCAACATCTATTT

>OTU_12

GCTGGCTGGTAATCTTGCCCACGCAGGAGCCTCTGTTGATCTAACAATTTTTTCACTCCATTTAGCAGGGGTTTCCTCAA

TTCTAGGGGCAATTAACTTTATCACAACATCAATTAACATAAAACCCCCTTCCATAACACAGTATCAGACACCCCTGTTT

GTGTGATCAGTATTAATCACGGCTATTCTTCTACTTCTATCCCTTCCGGTACTCGCAGCAGGTATTACAATGCTATTAAC

CGATCGAAACCTCAATACCACCTTCTTTGACCCCGCTGGCGGAGGGGACCCCGTGTTGTATCAGCATTTATTC

>OTU_13

TTTAGCATCTAGTTTATTCCACGGGGGATCAGCAGTTGATTTAGCTATTTTTTCTTTACATTTGGCTGGGGCCTCTTCTA

TTTTAGGTGCAATTAATTTTATTACTACTGTAATTAATATACGAACGTATGGGATAATTTTTGAGCGAATACCTTTGTTT

GTTTGATCCGTAGTGATTACTGCATTACTTTTACTTTTATCATTGCCTGTATTAGCCGGGGCTATTACTATGCTTCTTAC

TGACCGGAATTTTAATACTACTTTTTTTGATCCGGCCGGTGGGGGCGACCCAATTCTATACCAGCATTTATTT

>OTU_15

ATTAAGAAGATCTATCGCCCATAGAGGAGGGGCTGTCGACCTCGCTATTTTTTCACTTCATTTAGCCGGTGCCTCTTCTA

TTTTAGGGGCAATCAACTTTATCTCCACCGTTATTAATATACGATCTACTAATATATACATAAGACGAGTTCCTTTATTT

GTGTGATCTGTCTTTATCACCGCTATTTTACTACTACTATCTCTACCTGTTTTAGCTGGTGCTATCACTATATTATTAAC

AGACCGAAATATTAATACATCATTTTTTGATCCTTTAGGGGGAGGAGACCCTATCTTATACCAACATTTATTT

>OTU_16

TCTCTCGAGTAACATTGCTCACGCAGGGAGCTCTGTAGACTTTGCTATTTTTTCCCTTCATTTAGCCGGTGTGAGGTCAA

TTTTAGGAGCAGTGAATTTTATTAGAACCGTTGGAAACTTGCGAACTTTTGGTATAGTTCTTGACCGAATGCCTCTTTTT

GTGTGGGCTGTCTTAATTACAGCAGTGCTATTGCTACTGTCTTTGCCCGTTTTAGCCGGCGCAATTACAATGTTATTAAC

TGATCGTAATTTAAATTCCTCTTTCTACGACCCCAGAGGGGGCGGAGACCCGATTTTATACCAACACTTATTT

>OTU_17

CTTAGCAGGGAACTACTCCCACCCTGGAGCCTCCGTAGACCTAACCATCTTCTCCTTACACCTAGCAGGTGTCTCCTCTA

TCTTAGGGGCCATCAATTTCATCACAACAATTATCAATATAAAACCCCCTGCCATAACCCAATACCAAACGCCCCTCTTC

GTCTGATCCGTCCTAATCACAGCAGTCCTACTTCTCCTATCTCTCCCAGTCCTAGCTGCTGGCATCACTATACTACTAAC

AGACCGCAACCTCAACACCACCTTCTTCGACCCCGCCGGAGGAGGAGACCCCATTCTATACCAACACCTATTC

>OTU_18

TCTTTCAAGTAATATTGCTCACGCAGGGAGTTCTGTTGACTTTGCTATTTTTTCTCTTCACTTAGCTGGTGTTAGTTCAA

TTTTAGGCGCAGTAAATTTTATTAGAACTGTTGGCAACTTACGAACCTTTGGTATAGTTCTTGACCGCATGCCTCTTTTT

GTCTGGGCAGTTTTGATTACGGCGGTATTACTTTTATTATCATTACCTGTTCTAGCTGGAGCTATTACTATATTATTGAC

AGACCGTAATTTAAATTCATCTTTCTACGATCCCAGAGGGGGCGGGGACCCAATCCTGTATCAGCACTTATTT

>OTU_19

TCTTTCTTCTTGATTAGGACACTCTAATCATAGAGTAGATCTTGTGATTTTTTCTTTACATTTAGCTGGGGTTTCTTCGA

TCGCCAGTAGGATTAATTTTATTTCAACTTGTTTTTTCAATCGAAGTCTTATTTTCACTTTAGATCGCGTATCGATATTT

GTATGAAGAGTAATTATTACCTCTTTCATACTTATTATTTCACTTCCAGTTTTGGCAGGAGGAGTTACTATATTATTGTT

AGATCGAAATTTTGGGGGTTCTTTTTTTGAGGTCTCAGGTGGAGGAGATCCTATTCTTTTTCAACATATTTTT

>OTU_20

TCTGGCAGGAAACATCGCTCACGCCGGCGCTTCTGTAGATCTGGCAATTTTTTCACTACATCTTGCAGGAGTCTCCTCAA

TCTTAAGGGCAATCAACTTTATTACCACATCAATTAATATACAACCCCCATCAATAACACAATAACAAACATCAGTATTA

ATTACTGCCTTTCTTCTTCTCCTTTCTCTGCCCGTGCTTGCAGCAGGAATCACTATACTTCTCACTGACCTAAACCTAAA

CACTACATTTTTTGACCCTGCCGGAGGAGGAAACCCAGTGCTTTACCAACACCTGTT

>OTU_24

CCTATCTAGTAATATTGCTCATGCAGGGAGATCTGTTGATTTCGCAATTTTTTCTTTGCACTTAGCGGGTGTAAGATCTA

TTCTTGGGGCTGTAAATTTTATTAGAACTGTTGGGAATCTACGAGTATTTGGAATAATCTTGGATCGAATACCTTTGTTC

GCATGAGCTGTTTTAATTACAGCTATTTTATTGCTTTTATCTCTGCCTGTTTTAGCAGGGGCTATTACAATACTTCTTAC

AGATCGAAACCTTAACTCTTCATTCTATGATGCAAGAGGGGGTGGTGATCCAATTCTTTACCAACATTTATTT

>OTU_25

GCTTAGAAGATCTGTAGGTCACCCAGGAGGTGCTGTAGATCTAGCTATTTTTTCACTACATTTAGCAGGTGCTTCTTCAA

TTCTAGGAGCCATTAATTTTATCTCTACTATTATTAATATACGACCAAGAGGAATATCTATAGACCGTATACCTTTATTT

GTGTGGTCGGTTTTTATTACTGCCGTGCTTCTATTATTATCTTTACCTGTGTTAGCTGGAGCTATTACTATACTACTAAC

TGACCGAAATCTTAATACCTCATTCTTTGATCCTTTAGGTGGAGGTGATCCTATCTTGTACCAGCACCTGTTC

>OTU_32

TTTATCTGGCAACGTTGCACACGCTGGACCTGCAGTTGATCTCACTATTCTATCCCTTCACCTTGCAGGTGTTTCCTCGC

TCTTAGGGGCTATTAACTTTACAACAACCATTATAAATAGACGGATAGAAGGAATACCTTCTGAAAAAATACCATTATTT

ATTTGGTCCGTATTAGTAACAGTTGGACTACTAATTCTCGCACTACCTGTTCTGGCGGGAGCACTAACAATGCTCATTTT

AGATCGTAATTGCAATACTACATTCTTTGAGCCGACTGGCGGAGGAGATCCAATTTTATTCCAGCATTTATTC

>OTU_35

TCTATCGAGTAACATTGCTCACGCAGGGAGCTCTGTAGACTTTGCTATTTTTTCTCTTCATTTAGCCGGTGTGAGGTCAA

TTTTAGGAGCAGTGAATTTTATTAGGACCGTTGGAAACTTGCGAACTTTTGGTATAGTTCTTGACCGAATGCCTCTTTTT

GTGTGGGCTGTCTTGATTACAGCAGTGTTACTCCTACTGTCTTTGCCCGTTTTAGCCGGCGCAATTACAATGCTATTAAC

TGATCGTAATTTAAATTCCTCTTTCTACGATCCCAGAGGGGGCGGAGACCCGATTTTATATCAACACTTATTT

>OTU_42

ATTATCCGGTAATACAGCCCATGCTGGTCCCGCAGTAGATTTTACTATCTTATCTCTACATCTCGCAGGACTTTCGTCTC

TTATAGGCGCTATTAACTTTACTACTACTATCACAAATAATCGACTCACAGGGATATCTCTAGATAAAATACCCTTATTT

ATTTGATCTGTTCTTATTACAGTCGGCCTCCTCATTTTAGCCCTTCCCGTTCTCGCAGGAGCCCTTACCATGCTAATTAT

AGACCGAAACTGTAACACATCCTTCTTCGAACCTATGGGGGGCGGAGACCCTATTCTCTTTCAACATTTATTC

>OTU_44

TTTATCCGGGAATGTTGCACACGCCGGCCCAGCAGTAGATTTAACTATTTTATCCCTTCATCTAGCTGGGGTATCATCTC

TTCTAGGTGCAATTAACTTTACAACTACAATTATAAATAGACGTATAGAGGGAATACCTTCAGAAAAAATGCCTTTATTT

ATTTGATCTGTGTTAGTGACAGTAGGACTATTAATCTTAGCATTACCTGTTCTAGCAGGAGCATTAACAATATTAATTTT

AGACCGTAATTGTAATACATCCTTTTTCGAGCCCACAGGAGGAGGGGATCCTATTCTATTTCAACACTTATTC

>OTU_45

TTTAAGAAGATCTATTGCCCATAGAGGAGGAGCTGTAGATCTAGCAATTTTCTCATTACATCTAGCAGGGGCATCATCCA

TTTTAGGTGCTATTAATTTTATCTCAACCATCATCAATATACGATCAGCCAATATATTTATAAGACGGGTGCCTTTATTT

GTTTGATCTGTTTTTATTACAGCAATTTTATTACTTTTATCACTCCCTGTTTTAGCCGGGGCTATTACAATATTATTAAC

AGATCGAAATATTAATACATCTTTTTTTGACCCTTTAGGTGGAGGAGACCCTATCTTATATCAACACTTGTTT

>OTU_46

CCTAGCAGGAAATCTTGCCCACGCAGGAGCTTCTGTAGACCTTACCATCTTCTCTCTTCATCTTGCAGGAGTCTCTTCTA

TTCTAGGAGCAATTAACTTCATCACAACTATCATTAACATGAAACCCCCAGCAATCTCACAGTACCAAACACCTCTTTTC

GTATGAGCCGTTTTAATTACTGCTGTACTCCTCCTGCTCTCCCTTCCAGTCCTTGCAGCAGGGATTACAATGCTTCTCAC

TGACCGAAACTTAAATACAACCTTCTTCGACCCAGCAGGAGGAGGAGACCCCATCCTGTACCAACACTTATTC

>OTU_48

TCTCTCTAGAAATATTGCTCACTCAGGAGCTTCCGTAGATTTATCGATTTTTTCTTTACACTTAGCGGGAGCTTCATCAA

TTTTAGGAGCTATTAATTTTATATCAACTGTTATTAATATGCGAGCTGAAACATTAACATTTGATCGTCTTCCATTATTT

GTGTGAAGAGTATTTATTACCGTAATTCTTCTATTACTTTCACTACCAGTATTAGCTGGAGCAATTACAATGTTATTAAC

TGACCGAAACCTAAATACTTCATTTTTTGACCCAACTGGAGGAGGTGACCCTATCTTATACCAACATTTATTT

>OTU_51

TTTGTCAAGAAATATTGCACATTCTGGTGCTTCTGTAGATCTCTCAATTTTCTCGCTTCACCTAGCCGGAGCATCTTCAA

TTTTAGGAGCAATTAATTTTATATCAACAGTTATTAATATACGATCTGAAACTTTAACTTTTGATCGTCTACCTTTATTT

GTTTGAAGAGTCTTTATTACTGTAATTCTACTCCTTTTATCATTACCTGTATTAGCAGGTGCTATTACTATATTATTAAC

CGACCGAAATTTAAATACTTCTTTCTTTGATCCCACAGGAGGTGGAGATCCAATTTTATACCAACATTTATTC

>OTU_53

TTTAAGAAGATCTATAGCCCATAGAGGAGGAGCTGTAGACCTTGCTATTTTTTCACTACACCTGGCAGGTGCTTCCTCTA

TCTTAGGGGCTATTAATTTTATTTCTACTGTAATTAATATACGATCCACGAATATATATATAAGACGAGTGCCTTTATTT

GTTTGATCAGTCTTTACCACTGCTATTTTACTACTTTTATCTCTCCCTGTCTTAGCAGGCGCTATCACCATGCTACTAAC

AGATCGAAATATCAATACATCTTTTTTTGACCCTTTAGGAGGGGGAGACCCTATCCTGTACCAACATTTATTT

>OTU_55

TTTATCTACATCTTTAATGGCCTTATCTCCTATTTCAGTAGATCAAATGGTTGTTGCTTTAGCATTAGCAGGTATATCTA

GTTTACTATCATCATTAAACTTTGTAACAACTACATGGTTTTTAGGATTCTGTATTAATGATAAATCAAATGCTGTATTC

GTATATGCTATTTTATTTACTGCCATTATGTTAATATGTACTTTACCTATTTTAACTGCTGGTTTACTTATGATTGTATT

CGATCTACATTTAAATACTCATTTTTACGATCCTTTCTTTAACGGAGATCCAGTATTATACCAACATTTATTC

>OTU_59

TTTATCCAGTGCTGTAGCCCATAGCGGGGGCTCTGTAGATTTAGCTATTTTCTCGTTACACTTGGCAGGTGCCTCATCAA

TTTTAGGTGCTATTAATTTTATTTCTACCGTTATCAATATGCGAACAAAAGGAATATATATGTCTCGTATGCCTTTATTT

GTTTGGTCAGTATTTATTACTGCCATCTTATTATTATTATCTTTACCTGTTTTAGCAGGGGCGATTACAATACTATTAAC

AGATCGAAATTTAAATACATCTTTTTTCGACCCCAGCGGGGGAGGAGACCCTATTTTATACCAACATTTATTT

>OTU_64

ATTAAGGAGATCTATCGCCCATAGAGGAGGAGCTGTTGACCTCGCTATTTTTTCACTTCATTTAGCTGGTGCATCTTCTA

TTTTAGGGGCAATCAACTTTATCTCCACCGTTATCAATATACGATCTACTAATATATATATAAGACGAGTTCCTTTATTT

GTGTGGTCTGTCTTTATTACCGCTATTTTATTACTTCTATCTCTACCTGTTTTAGCTGGTGCTATCACTATATTATTAAC

AGACCGAAATATTAATACATCATTTTTTGATCCTTTAGGGGGAGGAGACCCTATCTTATACCAACATTTATTT

>OTU_65

ATTAAGGTCAACTTTAGGCCACTCCGGGGCTTCAGTAGACTTTGCAATTTTCTCTTTGCATTTAGCAGGAATTTCTTCAT

TATTAGGAGCAGTAAATTTTATTAGAACTCTTAAAAATACTCGCACATTTGGGCTTGTATTAGACCGGACAAGGCTTTTT

CCTTGATCAGTGTTAGTAACAGCTATTTTGCTTTTATTATCTCTCCCTGTCTTGGCAGGAGCTATTACAATGTTATTAAC

AGATCGAAATTTAAATACCTCATTTTATGACCCGAGAGGGGGAGGAGATCCCGTGTTGTACCAACATTTGTTT

>OTU_67

TCTATCTAGGAATATTGCTCACGCAGGAAGTTCAGTAGATTTTGCTATTTTTTCATTGCATTTAGCAGGAGTAAGTTCAA

TTTTAGGTGCGGTTAATTTTATTAGAACATTAGGAAATTTACGAGTGTTTGGAATATTATTAGACCGAATACCTTTATTT

GCATGGGCGGTATTAATTACAGCAGTTCTACTATTATTATCTCTACCTGTCTTAGCTGGGGCTATTACTATATTATTAAC

AGATCGAAATTTAAATACAACTTTTTATGATGTTGGGGGTGGTGGGGATCCTATTTTATATCAGCATCTATTT

>OTU_70

ATTAAGAAGTTCTATCGCCCATAGGGGAGGAGCTGTTGACCTTGCTATTTTTTCACTTCATTTAGCTGGTGCCTCTTCTA

TTTTAGGGGCAATCAACTTTATCTCTACCGTTATTAATATACGATCTACTAATATATACATGAGACGAGTTCCTTTATTT

GTGTGATCTGTCTTTATTACCGCTATTTTACTACTACTGTCCCTACCTGTTTTAGCTGGTGCTATCACTATATTATTAAC

AGACCGAAATATTAATACATCCTTTTTTGATCCTTTAGGGGGAGGGGACCCTATCCTATACCAACATTTATTT

>OTU_72

ATTGGCCGGAAACGGGGCCCACGGTGGGCCATCTGTCGACTTAGCAATCTTTTCTCTTCACCTCGCAGGAATTTCATCAA

TTCTTGGAGCCCTAAACTTTATCACTACTGTGATTAATATGCGCTGAACCGGATTACGCCTAGAACGCATTCCCTTATTC

GTGTGGGCTGTAGTTATCACAGCTGTACTGCTCCTACTGTCCCTTCCAGTTCTAGCGGGGGCAATTACAATACTTCTCAC

AGACCGCAACCTCAACACCTCATTCTTCGACCCTGCTGGGGGAGGGGACCCTATTCTCTATCAACACCTCTTC

>OTU_76

GTTATCTGCAAATTTAGCTCATGCTGGGGGTTCGGTTGACCTGGCAATTTTCTCATTACATTTAGCTGGTGTTTCTTCTA

TTTTGGGTGCTGTAAATTTCATTACTACTATTATTAATATGCGGTGACGGGGTATACAGTTTGAACGACTTCCTTTATTT

GTTTGATCAGTAAAAATTACAGCTATTTTATTACTACTTTCTCTTCCTGTTTTAGCGGGAGCTATTACAATATTGTTAAC

CGATCGAAATTTTAATACTGCTTTTTTTGACCCAGCAGGAGGTGGTGATCCAATTTTATATCAACATCTATTT

>OTU_77

TTTAAGAGGGGCAATTGCCCATAGAGGGGGTGCTGTAGATTTAGCAATTTTTTCTCTCCATCTAGCGGGTGCCAGTTCAA

TTTTAGGTGCCATTAATTTTATCTCCACCGTAATTAATATACGAGCTGAAAATATATATATAGACCGAATCCCTTTATTC

GTCTGATCTGTATTTATTACAGCTATTTTATTACTACTATCCCTCCCTGTATTAGCGGGTGCAATTACAATACTTCTAAC

AGATCGAAATTTAAATACATCTTTTTTTGACCCATTAGGCGGGGGGGACCCTATTTTATATCAACATTTATTT

>OTU_79

ATTAACTTCTATGCAAGCCCACAGTGGACCTTCAGTAGATTTAGCTATCTTTTCTCTTCATCTAGCTGGTATTTCATCTA

TCTTAGGAGCTGTTAATTTTATTTCAACAATTATGAATGCAAGAAAATATAATAATTCTACTAATCAAATCACCTTGTAT

TGTTTTTCAATCTTTATTACTACTATTTTATTACTTCTTTCCCTACCTGTATTAGCTGGCGCTATTACTATACTTCTAAC

TGACCGAAATCTCAATACATCATTTTTTGATCCTATAGGAGGTGGAGATCCTATCTTATACCAACACTTATTC

>OTU_80

TTTAAGAAGAAATTTAGCTCATGCTGGAGGCTCAGTTGATTTGGTGATTTTCTCTTTACATCTGGCTGGGGTGTCTTCTC

TTTTAGGTGCTGTAAATTTTATTAGAACTTTGAGAAATTTACGAGTATTTGGGATGTACTTTGACCAGGTTCCTTTGTTC

TGTTGATCTGTGCTAGTAACTGCTGTGTTGCTTTTATTATCACTTCCGGTACTAGCGGGGGCTATTACTATGCTATTAAC

GGATCGGAACATTAATTCAAGTTTTTATGATGTTAGGGGAGGAGGAGACCCTATTCTTTATCAGCATCTGTTT

>OTU_83

CCTATCAGCCGGAATCGCTCACGCGGGAGCTTCTGTTGACCTTAGTATTTTTGCTCTTCATTTGGCTGGGATTTCATCAA

TTCTAGGAGCTATTAATTTTATTACAACGATTGTTAATATACGATCTCACGGGATAACACTAGATCGAATTCCCCTATTT

GTTTGATCTGTTGGAATTACCGCTCTCCTTCTCCTATTAAGTTTACCCGTTCTAGCAGGAGCTATTACTATGCTACTTAC

AGACCGAAACTTAAATACTTCATTCTTTGACCCAGCAGGAGGGGGAGACCCAATCCTTTACCAACATTTATTC

>OTU_86

TTTAAGATCAACTTTAGGCCACTCTGGGGCTTCAGTAGACTTTGCAATTTTTTCTTTGCATTTAGCAGGAATCTCTTCAT

TATTAGGAGCAGTTAATTTTATTAGAACTCTTAAAAATACTCGCACATTTGGGCTAGTATTAGACCGGACAAGGCTTTTT

CCTTGGTCAGTGTTAGTAACAGCTATTTTGCTTTTATTGTCTCTTCCGGTCTTAGCAGGAGCTATTACAATGTTATTAAC

AGATCGAAATTTAAATACTTCATTTTATGATCCAAGAGGAGGAGGAGATCCCGTGTTGTACCAGCATTTGTTT

>OTU_91

TTTAAGAAGATCTATCGCTCATAGAGGAGGAGCTGTGGACCTTGCTATTTTTTCGCTACACCTGGCAGGTGCTTCTTCTA

TCCTAGGGGCTATTAATTTTATTTCTACTCTAATTAATATACGATCCACGAATATATATATAAGACGAGTGCCTTTATTT

GTTTGATCAGTCTTTATCACTGCTATTTTACTACTTTTATCTCTCCCTGTTTTAGCAGGTGCTATCACCATGCTACTGAC

AGATCGAAATATCAATACATCTTTTTTTGACCCTTTAGGAGGGGGAGACCCTATCCTGTACCAACATTTATTT

>OTU_96

GTTTTTTTAACTGTGCCCCAAATACTTGCCTCTTCTAAGACTTTGTAATTTATCCGAACAGATATCCTCTTCCTTTGATA

CAATGGATCCCTTTGGTACAGGACAGGTGCTTCCTCCTCTGCAAGTTCCACATCCCACAGTTCAGGACACTATAGTTCCC

CCTCTGAAGAAACCACCTAAATGGATGCGCAGGCCGGTGGGGGCTTCCTTTGGTGTAAGTAAATAGGTCAGGGAAAGATG

ATGATCATTTTCCAAATAAC

>OTU_101

TCTTTCTATTACTAGTAATTCTTTTACCATTGAAGTTTCCTTTTTCAGGATTCATTTAAATAGCTTATCTTCTGTTCTTG

GTTCTTTTAATATTGTATTCTCTGTTTTTTCTATTGGATTTAGAGTATCCACTTCTTTAGTGTATTCTTTCCTATCTTCT

AGTATTCTTGTCCTTATCAGTATTCCTTCTTTCATTCTAGCCACTCTTTTAATTATTTTCTTCCGTTCTTATAATTCTGC

TTACCTTATTCCAGAACTCTACATACACCTATTC

>OTU_102

TTTGAGGTCCACATTGGGCCATTCAGGGGCTTCGGTAGACTTCGCCATTTTCTCGTTACACCTGGCAGGGGTGTCTTCTT

TGCTTGGAGCAGTTAATTTTATTAGGACCTTAAAAAATACTCGGACGTTTGGGCTAGTTTTAGATGCTATGAATTTATTT

CCTTGATCTGTCTTAGTAACTGCAATTCTTCTACTACTATCTTTACCGGTACTTGCAGGAGCCATTACAATGCTTTTAAC

AGACCGAAACCTTAATTCCACTTTTTATGATCCTAGAGGAGGGGGAGACCCAGTGCTTTACCAACATCTATTC

>OTU_106

TTTATCTAGTAATTTAGCTCACGCTGGTGGTTCAGTTGATTTAGCAATTTTTTCACTTCATTTAGCAGGTGTATCTTCGA

TTTTAGGGGCTGTAAATTTTATTACAACTATTATTAATATACGATGACGAGGGATACAGTTTGAACGTTTACCTCTATTT

GTTTGATCAGTAAAAATTACTGCTATTTTATTACTTCTTTCTTTACCTGTATTAGCTGGAGCTATTACAATGCTTCTAAC

GGACCGAAATTTTAATACTGCTTTTTTCGATCCAGCAGGAGGTGGTGATCCAATTTTATACCAACACTTATTT

>OTU_111

TTTAAGGTCGACCTTAGGTCATTCAGGCCCTTCTGTAGATTTCGCAATTTTTTCTTTACACTTAGCAGGAATTTCTTCTT

TGTTAGGAGCAGTAAATTTTATTAGAACTTTAAAAAACACACGGTCTTTTGGTTTGGTTTTAGACCGCATAAGAATATTT

CCATGGTCTGTTCTTATCACTGCAATTTTATTATTACTATCTTTACCTGTTTTAGCAGGAGCTATTACAATACTTTTAAC

TGATCGAAATTTAAATACAACCTTCTACGATCCTAGAGGCGGGGGAGATCCTATTTTATATCAACACTTATTT

>OTU_114

TTATCAAGTGCAACAGCTCACTCTGGGGGTTCAGTAGACTTAGCAATATTTAGCTTACATTTATCAGGTGCATCTTCTAT

TTTAGGTGCTATTAATTTCATCTGTACTATTTTTAACATGCGAGTGAAAAGTTTATCTTTTCATAATCTTCCCCTATTTG

TATGATCTGTTTTAATTACTGCATTTTTATTGCTATTATCTTTACCTGTACTAGCTGGGGCGATCACAATGTTATTAACG

GATAGAAATTTCAATACTACCTTTTTTGACCCAGCTGGTGGAGGAGACCCTGTGTTATTCCAACACCTTTTT

>OTU_115

GTGAACAAGACCCCGAGGTACTTGAACTCCTCCACTTGGGGCAGGATCTCCTCCTTGACCCGGAGAAGGCACTCCACCTT

TTTCCGGTTGAGAACCATGGCCTCGGATTTGGAGGTGCTGATTTTCATCCCAGCCGCTTCACATGCGGCGGCGAACCGAT

CCAGTGATAGTTGGAGGTCACGGGCTGATGACGCCAACAGGACCACATCATCCGCAAAAAGCAGAGACC

>OTU_116

TTTATCGAGCGCTACAGCGCATTCGGGAGGTTCTGTAGACTTAGCTATTTTTAGCCTACATTTATCTGGTGCATCTTCTA

TCTTAGGAGCTATTAACTTCATTTGCACCATTTTTAATATGCGTGTTAAAAGTTTATCTTTTCATAACTTACCATTATTT

GTATGATCTGTTTTAATTACTGCGTTTCTATTGTTATTATCATTGCCTGTATTAGCAGGAGCAATTACAATGTTGTTAAC

AGATAGAAATTTCAACACTACTTTTTTTGACCCTGCTGGTGGTGGCGACCCAGTACTATTCCAACATCTTTTT

>OTU_125

ATTATCTTCAAATATAGCTCAGAGTGGACCAAGAGTAGATTTAGCGATATTTTCGCTTCATTTAGCTGGAGTAAGGTCAA

TATTGGGATCCATAAAATTTATAACTACTATGGTTAACGCTAAGATGCAAGTAAGATGAGGACAAATGCCGTTGTTTTTA

TGAGCTGTTTTAGTGACAGCTTATATGTTAGTATTATCTCTTCCAGTTTTAGCCGGAGGGCTAACTATGTTATTAACAGA

TCGAAATTTTAATACTACTTTTTTTGATCCTGGAGGAGGGGGAGACCCTATTCTATTTCAACATATATTC

>OTU_130

TTTGTCTAGTAATATTGCTCATTCAGGGGCTTCCGTAGATTTGTCCATTTTTTCTCTTCATCTAGCTGGGGCTTCTTCTA

TTCTTGGGGCGATTAATTTTATATCTACTGTGATCAATATGCGTGCTGAAACTTTAACCTTTGATCGAATTCCTCTTTTT

GTTTGAAGAGTTTTTGTGACAGTAATTTTACTACTATTATCTTTACCAGTTTTAGCTGGGGCAATTACTATGCTTTTAAC

TGATCGTAATTTAAATACTTCCTTCTTCGACCCAACAGGAGGAGGAGATCCTATTCTTTATCAGCATCTATTT

>OTU_141

TTTAATGCTAGGAGATTACAGATCAGGGGTTTCTGTTGATTTAATAGTTTTAGGTCTTCATATAGCAGGATTGTCATCAA

TTTTAGGGTCTATTAATATTTTAGTGACATGGGTTATGGGGAGGAGTGCAGCTTACAGGGTAGAGCATGTTAGGTTATTT

GTGTGAGCTTTGGTTGTTACAGCTGCTTTAGTGGTCTTAACTGTTCCCGTGTTAGCTGCTGCTCTAACAATAATTTTAAT

AGATCGTAATTTGAGGACTAGTTTTTTTTGATCCTTGTGGAGGGGGTTCACCTATTTTGTATCAGCATTTGTTT

>OTU_143

TTTAAGAGCAGCTATCGCACATAGAGGGGGTGCAGTAGATTTAGCTATTTTTTCTTTACATTTAGCAGGAGCTTCTTCAA

TTCTAGGTGCAATCAATTTTATCTCCACAGTAATTAATATACGTGCAGCAGGAATAACTATAGACCGTATACCTTTATTT

GTTTGGTCTGTTTTTATTACAGCAATCTTATTATTATTATCTTTACCTGTATTAGCAGGAGCTATTACTATACTGTTAAC

TGACCGTAATTTAAATACATCTTTTTTCGATCCTTTAGGGGGAGGAGACCCAATTCTTTACCAACATTTATTT

>OTU_144

TCTTTCAAGAAATTTAGCTCATGCTAGACCTTCGGTTGACTGTGCTATCTTTTCTCTTCACTTAGCTAGAGTTTCAAGTA

TTCTAGGATCAATTAACTTCCTAGTAACTATGATAAACATAATAAGTAAAGGTCAAACATACGGTAACCTTTCTCTATTT

TGTTGATCTATTGTTGTTACAACAATTCTACTAATTTTATCATTACCAGTACTAGCAGCAGCTATTACTATACTTCTATT

TGACCGAAACTTCAATACTTCGTTCTTTGATCCTGCTAGAAGAAGGGATCCTATCCTTTACCAGCATCTATTC

>OTU_145

ATTATCAAGCGCAACAGCTCACTCTGGGGGTTCTGTAGATTTAGCGATATTCAGCTTGCATTTATCTGGTGCTTCTTCTA

TTTTAGGAGCTATTAATTTTATTTGTACTATTTTTAATATGCGAGTAAAAAGCCTATCTTTTCATAACCTTCCTCTATTT

GTGTGATCTGTTTTAATTACAGCATTTTTACTGTTACTATCTTTGCCCGTATTGGCAGGAGCAATAACAATGTTATTGAC

AGATAGAAATTTCAACACTACCTTTTTTGATCCTGCTGGAGGAGGTGATCCTGTATTATTTCAGCACCTTTTT

>OTU_149

CTTGTCTGGGAATGTTGCACATGCAGGTCCTGCAGTAGATTTAACAATTTTATCTCTCCATCTAGCAGGTGTCTCATCTC

TGTTAGGGGCTATTAACTTTACAACTACTATTCTTAATAGACGAATAGAAGGCATACCTACTGAAAAGATACCACTCTTT

ATTTGATCGGTTCTAATTACTGTAGTTCTTCTAATTCTAGCCCTCCCTGTTCTAGCAGGTGCTCTAACTATACTTATTAT

AGACCGAAACTGTAATACATCCTTCTTTGAGCCTATAGGAGGAGGAGACCCAATTTTATTCCAACACCTCTTT

>OTU_168

TCAAACTGAAAACCGAGGAAGTGAAAATCCGCCAGATGTACTGGCAGTAGCCTAAAGGCAGACTCAATATCAAATTTTGC

CATCAAAGTCCCCTGCCCCAGACATCTCAGCATACACACAGCCTGGTCAAATGATGCATATTTTACACTGCATAGTGTTT

CTGAGATCGCCTCGTTGACAGATTGCCCTCTTGGCGCCGAGAGGTTGTGAATTAAGCGAAATTCCCCTTGCACTTTCTTG

GGCACGATTCCCAACGGTGAACAAATAAAATCCTGCATTGGCGGGTTCTCGAACTGGCCAGCTACCCTGCCTAAGCAAAC

CTCTTTCTCTATTTTCTTTCTGGCAATGGCCACATTTTCTCTGACCGACATC

>OTU_197

GGTTCACGCTTCGCCTGACTTAAAATTGGACAGATTAACTCTTAAAGTAATAGGAAACAGAGAACAAAATTAGATATCCT

GGATCAGAGCAGTGAAACGAAATGAGCGGAACGGAGTAACTCCTCATGACTGGCAGCTCTGAACGAGGCTTTCGTTTGTT

TAATACCGTCACCGTAAAGATGGAACCTGGCTGTGACGGAGGTGCACCTGCAGTTCTCCTCAAAATAACCAACATTAAAG

ACATTTCCATTCTTTTAATGAGCCGTTAATCTTTAAAACTAATAAAAAGTACTAATTCTTCTTACAATATAAGCTGCATG

TCAACAACAGTCGCAATAATAATGTAACTACACGA

>OTU_211

GCTATCATCTGTGGAATTCCACTCAGGTCCTTCAGTGGACCTAGCGATCTTCAGTCTACACCTGGCAGGTGCATCATCTA

TCCTAGGTGCGGTGAACTTCATCACCACAATCCTAAACAGGCGAGCACCGGGAAGGACAAGGCACCGACTGCCCCTATTC

GTATGGGCGGTTCTGATCACAGCGGTACTACTGCTGCTATCACTGCCAGTTCTAGCGGGAGCGATTACTAGGCTACTAAC

CGACCGGAACTTCAACACGGCATTCTTCGACCCAGCGGGTGGAGGTGACCCTGTACTGTACCAGCACCTATTC

>OTU_212

GCCCAGAGGTGAACTTTGACCTTTGAGAAGAATAGAAGGGAGACGGTGGGATGAGCGGCTGGATTGGAACCTCCAGATAT

GTGGACGTATGCTAGTTAGCACGTAGCTGCAGCCATCCAGACGCTCTCCTCCCTTTGTCTCTCTTTCTAGGCCGCAGCGA

AGTGATCAATAAAGGTTTAAGAGGATAAACTCTACCCCTCTGTCACGTGACGCTTGGTCATGTGACCTCGGGAGAGGAAC

AAACAGGGGCGTGTGAGCGCAGGAGCTGTTGCATCAGCGTCAGTGCGTTTCTCATGGGTTCTCCTCAGTCTTTGTGCCGT

TTT

>OTU_245

TTTATCAAGAAATATTGCACATTCTGGTGCTTCTGTAGATCTCTCAATTTTCTCGCTTCATCTAGCCGGGGCATCTTCAA

TTTTAGGGGCAATTAATTTTATATCAACAGTTATTAATATACGATCTGAAACTTTAACTTTTGATCGTCTTCCTTTATTT

GTTTGAAGAGTTTTTATTACTGTAATTTTACTCCTTTTATCATTACCTGTTTTAGCAGGTGCTATTACTATATTATTAAC

CGATCGAAATTTAAATACTTCTTTCTTTGATCCCACAGGAGGAGGAGATCCAATTTTATACCAACATTTATTC

>OTU_248

TTTGTCGGGAGCTATGGGTCATAGTGCACCTTCTGTTGATTTAGTAATTTTTTCTTTACATTTGGCAGGGGTGTCTTCTA

TTTTAGGGGCCATTAACTTTATTACCACAATTATTAATATACGGGCCACGGGAATCTCTTTTGATCGAATAAGTTTATTT

GTATGATCAGTACTAGTAACGGCAGTCTTACTTTTATTATCACTTCCTGTTTTAGCAGGGGCTATTACAATATTATTAAC

AGATCGTAATTTTAATACTAGGTTCTTTGATCCTGCAGGTGGGGGTGACCCTATTCTTTATCAACATTTATTT

>OTU_254

ATTATCTGGTAACGTAGCTCACGCAGGTCCAGCAGTTGATTTAACAATTTTATCTCTTCATCTTGCTGGTGTTTCGTCTC

TAATAGGGGCAATTAATTTCACGACAACTATTCTTAATAGACGGATAGAAGGGATGCCAACGGAAAAAATACCTTTATTT

ATCTGATCTGTCCTCGTTACTGTAGGCCTATTAATTCTAGCTCTTCCAGTTCTTGCCGGAGCTCTTACTATACTTATTAT

AGACCGGAATTGCAATACTTCTTTCTTTGAACCAACAGGAGGAGGAGACCCCATTCTTTTCCAACACTTATTC

>OTU_266

TTTATCAAGTAACATTGCTCATTCTGGTGCTTCAGTTGACTTATCAATTTTCTCTTTACATTTAGCGGGTGCTTCGTCAA

TTTTAGGTGCCATTAATTTTATGTCTACAGTTATTAACATACGAGCTGAAACACTGACATTTGATCGACTTCCATTATTT

GTCTGAAGAGTATTTATTACTGTAATTCTTTTACTTTTATCACTTCCAGTACTAGCAGGAGCTATTACTATGTTGCTAAC

AGATCGAAATCTGAATACCTCATTTTTTGATCCAACAGGAGGTGGAGATCCAATCTTATACCAACATCTATTT

>OTU_307

GTTATCTTCCTATATATATAGGTCTTCTTATTCTGTAGATTTTGCTATTTTATCTTTACATTTAGCGGGTATCTCTAGTA

TTAATAGTTCTATTAATTTTTTTACAACATTTTTTTCTTATAAAAAATCAGGTATACTTTATTCACAAATTCCTTTGTTT

GTGTGAAGTGTAATTATCACAGCATTTTTATTATTATTTACTTTACCTGTTTTGGCAGGAGCAATTACAATATTATTAAC

AGATCGTAATTTTAATACCTCTTTTTTTGAGCCTTCTGGAGGTGGGGATCCTGTATTGTTTCAACACTTATTT

>OTU_344

TTTATCAAGAACAATCGCCCACGCGGGTGCTTCTGTAGACCTTACCATCTTCTCTCTTCATCTTGCAGGAGTCTCTTCTA

TTCTAGGAGCAATTAACTTCATCACAACTATCATTAACATGAAACCCCCAGCAATCTCACAGTACCAAACACCTCTTTTC

GTATGAGCCGTTTTAATTACTGCTGTACTCCTCCTGCTCTCCCTTCCAGTCCTTGCAGCAGGGATTACAATGCTTCTCAC

TGACCGAAACTTAAATACAACCTTCTTCGACCCAGCAGGAGGAGGAGACCCCATCCTGTACCAACACTTATTC

>OTU_348

CCTATCTAGGAATATTACTCACGCAGGGAGTTCAGTAGATTTTGCTATTTTTTCATTGCATTTAGCCGGAGTAAGTTCAA

TTTTAGGTGCGGTTAATTTTATTAGAACATTGGGAAATTTACGGGTGTTGGAATATTATTAGACCGAATACCTTTATTTG

CATGGGCGGTATTAATTACAGCAATTTTACTATTATTATCTCTACCTGTCTTAGCTGGGGCCATTACTATATTATTAACA

GATCGAAATTTAAATACAACTTTTTATGATGTTGGGGGCGGTGGGGATCCTATTTTATATCAGCATCTATTT

>OTU_355

TCTTTCAAGTAATATTGCTCACGCAGGGAGTTCTGTTGACTTTGCTATTTTTTCTCTTCACTTAGCTGGGGTTAGTTCAA

TTTTAGGTGCAGTGAATTTCATTAGAACTGTTGGCAACTTACGAACCTTTGGTATTGTTCTTGACCGTATGCCTCTTTTT

GTCTGGGCAGTTTTGATTACGGCGGTATTACTTTTATTATCATTACCTGTTCTAGCTGGAGCTATTACTATATTATTAAC

AGACCGTAATTTAAATTCATCTTTCTACGACCCTAGAGGGGGCGGAGACCCAATCCTGTATCAGCACTTATTT

>OTU_382

CATGTCTTTGGTTTAAAGACACAGCTTCACTGAGGTTATCGCACCTACTCACAAGGCTCATGGTGCTTCATTTTGCAGTT

ACAGAGAGAGGTGACCACACGGCGAGTTGAGGGCACCGAGGGCAACTCGGACCATAAAGGCGTATTTTCCACTCAGGACG

TCGCTCCCAGGTCACCTCGCCCCTGTACATCCACTCCAATCCCAGGGTCTTCCACTCGCTCCCACTC

>OTU_420

TTTATCAACTTACTCTTATCATGGAGTTTGTATAGACCTTGCAATTCTAAGCCTTCACCTTGCTGGTATTAGCTCTATTT

TCAGGTCAATTAATTTCATAGTAACGATTAGAAATATGCGATCTGTTGGGGGCCATTTACTAGCACTATTCCCTTGATCT

ATTAAGGTTACTTCATTCTTGCTTTTGACTACTCTCCCAGTGTTAGCTGGAGGTCTTACTATACTTTTGACTGATCGTCA

TTTTAATACCTCTTTTTTTGACCCTGTCGGAGGGGGGGACCCTGTCTTATTTCAGCATTTGTTT
