# Supplementary material for: Including planocerid flatworms in the diet effectively toxifies the pufferfish, Takifugu niphobles
Source: Sci Rep. 2018 Aug 17;8:12302. doi: 10.1038/s41598-018-30696-z (PMC6098040; doi:10.1038/s41598-018-30696-z)
Supplement: Supplementary file 3 — NGS_seq(2017).docx [file 41598_2018_30696_MOESM3_ESM.docx]

>OTU_1

TTTAGCTGCTGCTATTGCTCACGCAGGAGCATCTGTTGATTTAGGTATTTTCTCTTTACATTTAGCGGGTGTTTCTTCTATTTTAGGAGCAGTTAATTTTATAACAACAGTAATCAATATACGGTCATATGGTATAACTATAGATCAAATACCTTTATTTGTTTGATCAGTATTTATTACAGCTATTTTACTACTTTTATCACTCCCAGTTTTAGCAGGAGCTATTACAATATTATTAACAGACCGAAATTTAAATACATCATTCTTTGATCCAGCTGGTGGAGGAGATCCTATTTTATATCAACATTTATTC

>OTU_2

ATTGGCCGGAAACGGGGCCCACGGTGGGCCATCTGTCGACTTAGCAATCTTTTCTCTTCACCTCGCAGGAATTTCATCAATTCTTGGAGCCCTAAACTTTATCACTACTGTGATTAATATGCGCTGAACCGGATTACGCCTAGAACGCATTCCCTTATTCGTGTGGGCTGTAGTTATCACAGCTGTACTGCTCCTACTGTCCCTTCCAGTTCTAGCGGGGGCAATTACAATACTTCTCACAGACCGCAACCTCAACACCTCATTCTTCGACCCTGCTGGGGGAGGGGACCCTATTCTCTATCAACACCTCTTC

>OTU_3

TTTATCAAGAACAATCGCCCACGCGGGTGCTTCTGTAGATCTTAGAATCTTCTCCTTGCATTTAGCGGGGATTTCATCAATCCTCGGAGCTGTAAACTTTATTACGACAATTGTAAATATACGATCTAAAGGAATAACTTTAGATCGTATTCCCCTCTTTGTGTGGGCTGTTGGAATTACTGCTTTATTACTCCTACTTAGACTTCCTGTACTTGCAGGAGCTATCACTATGCTTCTGACAGACCGAAATTTAAATACTTCTTTCTTTGATCCTGCGGGAGGGGGGGACCCTATTCTTTATCAACACTTGTTC

>OTU_4

TTTGGCAGCGGCTATCGCTCACGCAGGAGCTTCTGTGGACTTGGGGATTTTTTCTCTTCATCTTGCTGGGGTTTCCTCTATCCTAGGCGCAGTTAACTTTATAACTACTGTTATCAATATACGTTCATATGGTATAACAATAGATCAAATACCTTTATTTGTTTGAGCGGTCTTCATTACTGCTATCCTCCTACTTTTATCCTTACCCGTTTTAGCAGGGGCTATTACGATACTTCTTACAGATCGCAACCTTAATACCTCTTTCTTTGATCCTGCTGGTGGTGGTGACCCAGTACTTTACCAACATTTATTT

>OTU_5

CTTATCAAGAAACATTGCACACTCAGGAGCATCAGTAGATCTTTCAATTTTCTCTCTACACTTAGCCGGAGCTTCCTCAATTCTTGGGGCCATTAATTTTATATCAACTGTTATTAATATACGTGCAGAAACTTTAACATTCGACCGTTTACCTTTATTTGTATGAAGAGTATTTATTACAGTTATTCTATTACTATTATCTCTGCCAGTTCTAGCGGGTGCAATTACTATGTTACTTACAGATCGAAATTTGAATACATCATTTTTCGATCCTACTGGAGGTGGAGACCCAATTCTCTACCAACACCTATTT

>OTU_6

TTTGTCTAGTAATATTGCTCATTCAGGGGCTTCCGTAGATTTGTCCATTTTTTCTCTTCATCTAGCTGGGGCTTCTTCTATTCTTGGGGCGATTAATTTTATATCTACTGTGATCAATATGCGTGCTGAAACTTTAACCTTTGATCGAATTCCTCTTTTTGTTTGAAGAGTTTTTGTGACAGTAATTTTACTACTATTATCTTTACCAGTTTTAGCTGGGGCAATTACTATGCTTTTAACTGATCGTAATTTAAATACTTCCTTCTTCGACCCAACAGGAGGAGGAGATCCTATTCTTTATCAGCATCTATTT

>OTU_7

TCTCTCGAGTAACATTGCTCACGCAGGGAGCTCTGTAGACTTTGCTATTTTTTCTCTTCATTTAGCCGGTGTGAGGTCAATTTTAGGAGCAGTGAATTTTATTAGAACCGTTGGAAACTTGCGAACTTTTGGTATAGTTCTTGACCGAATGCCTCTTTTTGTGTGGGCTGTCTTAATTACAGCAGTGCTACTGCTACTGTCTTTGCCCGTTTTAGCCGGCGCAATTACAATGTTATTAACTGATCGTAATTTAAATTCCTCTTTCTACGATCCCAGAGGGGGCGGAGACCCGATTTTATACCAACACTTATTT

>OTU_8

CCTAGCAGGAAATCTTGCCCACGCAGGAGCTTCTGTAGACCTTACCATCTTCTCTCTTCATCTTGCAGGGGTCTCTTCTATTCTAGGAGCAATTAACTTCATCACAACTATCATTAACATGAAACCCCCAGCAATCTCACAGTACCAAACACCTCTTTTCGTATGAGCCGTTTTAATTACTGCTGTACTCCTCCTGCTCTCCCTTCCAGTCCTTGCAGCAGGGATTACAATGCTTCTCACTGACCGAAACTTAAATACAACCTTCTTTGACCCAGCAGGAGGAGGAGACCCCATCCTGTACCAACACTTATTC

>OTU_9

TCTGTCTTTAAATTTAGCCCACGCGGGTATATCTGTAGATTTTGCCATTTTCTCTCTTCACTTGGCGGGTATCTCATCTCTTCTAGGGGCCGTAAACTTTATCAGTACACTAGGGAACCTGCGTTCTTTGGGGCTAATAATGGACCGTATACCCCTTTTTGCCTGGGCTGTGCTAGTCACAGCTATTTTATTACTCCTGTCCCTTCCCGTACTAGCAGGGGCCATCACCATGCTCCTGACGGATCGAAACCTCAACACGTCTTTTTATGACGTAAGAGGAGGAGGGGACCCAGTTCTCTACCAGCACCTGTTT

>OTU_10

CCTATCAGCCGGAATCGCTCACGCGGGAGCTTCTGTTGACCTTAGTATTTTTGCTCTTCATTTGGCTGGGATTTCATCAATTCTAGGAGCTATTAATTTTATTACAACGATTGTTAATATACGATCTCACGGGATAACACTAGATCGAATTCCCCTATTTGTTTGATCTGTTGGAATTACCGCTCTCCTTCTCCTATTAAGTTTACCCGTTCTAGCAGGAGCTATTACTATGCTACTTACAGACCGAAACTTAAATACTTCATTCTTTGACCCAGCAGGAGGGGGAGACCCAATCCTTTACCAACATTTATTC

>OTU_11

TCTTGCAGCCGCTATCGCTCACGCTGGTGCTTCAGTCGATATAGGTATTTTTTCACTTCACCTGGCCGGCGTTTCTTCAATTTTAGGAGCCGTAAATTTTATAACTACCGTTATTAACATACGATCTTTCGGAATAACTATAGACCAAATACCATTATTTGTATGAGCAGTGTTTATTACAGCTATTCTCTTACTTCTATCCTTACCAGTCCTAGCAGGTGCAATTACTATGCTCCTTACTGATCGTAATCTTAATACATCCTTCTTTGACCCAGCTGGAGGAGGGGACCCTGTTTTATACCAACACCTATTT

>OTU_12

CTTAGCCGGAAACGGGGCACACGGAGGACCTTCCGTAGACCTCGCTATTTTCTCGCTTCATCTTGCCGGTATTTCATCAATCTTAGGGGCCTTAAACTTCATTACAACCGTTATTAATATACGATGAACCGGACTACGCCTCGAACGAATCCCATTATTTGTCTGAGCTGTAGTCATCACAGCGGTTCTTCTTCTCCTCTCCCTCCCTGTTCTTGCCGGGGCTATTACAATGCTCCTAACAGACCGAAACCTTAATACCTCATTCTTCGACCCCGCTGGAGGAGGAGACCCTATTCTATACCAGCACCTCTTC

>OTU_13

TCTTTCAAGTAATATTGCTCACGCAGGGAGTTCTGTTGACTTTGCTATTTTTTCTCTTCACTTAGCTGGTGTTAGTTCAATTTTAGGCGCAGTAAATTTCATTAGAACTGTTGGCAACTTACGAACCTTTGGTATAGTTCTTGACCGCATGCCTCTTTTTGTCTGGGCAGTTTTGATTACGGCGGTATTACTTTTATTATCATTACCTGTTCTAGCTGGAGCTATTACTATATTATTGACAGACCGTAATTTAAATTCATCTTTCTACGATCCCAGAGGGGGCGGGGACCCAATCCTGTATCAGCACTTATTT

>OTU_14

TCTTTCCAGCAACTTAGCCCACGGGGGGGCATCTGTAGATCTCGCTATTTTCTCTCTGCATTTAGCCGGGATTTCTTCAATCTTAGGCGCTATCAATTTTATTACTACAGTTATTAATATGCGCTGACGTGCCATACTCTTTGAGCGTCTCCCTTTATTTGTCTGATCAGTAAAAATTACTGCAATCCTTCTTCTTCTTTCCTTACCTGTGCTAGCAGGTGCTATTACTATGCTTTTAACCGACCGAAACTTCAATACGGCGTTTTTTGACCCTGCAGGGGGAGGCGACCCTATTCTATATCAACATCTTTTC

>OTU_15

TTTATCAACTTACTCTTATCATGGAGTTTGTATAGACCTTGCAATTCTAAGCCTTCACCTTGCTGGTATTAGCTCTATTTTCAGGTCAATTAATTTCATAGTAACGATTAGAAATATGCGATCTGTTGGGGGCCATTTACTAGCACTATTCCCTTGATCTATTAAGGTTACTTCATTCTTGCTTTTGACTACTCTCCCAGTGTTAGCTGGAGGTCTTACTATACTTTTGACTGATCGTCATTTTAATACCTCTTTTTTTGACCCTGTCGGAGGGGGGGACCCTGTCTTATTTCAGCATTTGTTT

>OTU_16

TCTTAGGACTATGGGTCACCCGGGCAGGAGTGTAGACTTGGCTATTTTTAGTTTACATTGTGCTGGTGTGAGGTCTATTTTGGGTGCTGTTAATTTCATGACTACTACAAAAAACCTTCGTAGGAGCTCTATTTCTTTGGAGCACATGAGTCTTTTTGTTTGAACTGTCTTTGTTACTGTGTTTCTCTTGGTCCTTTCTTTGCCTGTTTTGGCGGGAGCTATTACAATGTTGTTGACTGATCGTAATCTTAATACTTCTTTTTTTGATCCTAGAACTGGCGGTAACCCGCTTATTTATCAGCATCTTTTT

>OTU_17

ATTGGCCGGAAACGGGGCCCACGGTGGGCCATCTGTCGACTTAGCAATCTTTTCTCTTCACCTCGCGGGAATTTCATCAATTCTTGGAGCCCTGAACTTTATCACCACTGTGATTAATATACGCTGAACTGGATTACGCCTAGAACGTATTCCCTTATTCGTGTGGGCTGTAGTTATTACAGCTGTGCTGCTCCTACTGTCCCTTCCAGTTCTAGCAGGGGCAATTACAATACTTCTCACAGACCGTAACCTCAACACCTCGTTCTTCGACCCTGCTGGGGGAGGGGACCCTATTCTCTATCAACACCTCTTC

>OTU_18

TCTCTCTAGAAATATTGCTCATTCAGGAGCTTCCGTAGATTTATCGATTTTTTCTTTACACTTAGCGGGAGCTTCATCAATTTTAGGAGCTATTAATTTTATATCAACTGTTATTAATATGCGAGCTGAAACATTAACATTTGATCGTCTTCCATTATTTGTGTGAAGAGTATTTATTACTGTAATTCTTCTATTACTTTCACTACCAGTATTAGCTGGAGCAATTACAATGTTATTAACTGACCGAAACCTAAATACTTCATTTTTTGACCCAACTGGAGGAGGTGACCCTATCTTATACCAACATTTATTT

>OTU_19

TTTAAGAAGAAATTTAGCTCATGCTGGAGGCTCAGTTGATTTGGTGATTTTCTCTTTACATCTGGCTGGGGTGTCTTCTCTTTTAGGTGCTGTAAATTTTATTAGAACTTTGAGAAATTTACGAGTATTTGGGATGTACTTTGACCAGGTTCCTTTGTTCTGTTGATCTGTGCTAGTAACTGCTGTGTTGCTTTTATTATCACTTCCGGTACTAGCGGGGGCTATTACTATGCTATTAACGGATCGGAACATTAATTCAAGTTTTTATGATGTTAGGGGAGGAGGAGACCCTATTCTTTATCAGCATTTGTTT

>OTU_20

ATTAAGAAGATCTATCGCCCATAGAGGAGGGGCTGTCGACCTCGCTATTTTTTCACTTCATTTAGCCGGTGCCTCTTCTATTTTAGGGGCAATCAACTTTATCTCCACCGTTATTAATATACGATCTACTAATATATACATAAGACGAGTTCCTTTATTTGTGTGATCTGTCTTTATCACCGCTATTTTACTACTACTATCTCTACCTGTTTTAGCTGGTGCTATCACTATATTATTAACAGACCGAAATATTAATACATCATTTTTTGATCCTTTAGGGGGAGGAGACCCTATCTTATACCAACATTTATTT

>OTU_21

TCTATCAAGTAATATTGCACATTCTGGTGCTTCTGTAGATTTATCTATTTTTTCTTTACACTTAGCCGGGGCATCTTCAATTTTAGGAGCAATTAACTTTATATCAACAGTAATTAATATACGATCTGAAACTCTTACTTTTGACCGACTTCCTTTATTTGTTTGAAGTGTATTTATTACCGTAATTTTACTTCTTTTATCATTACCTGTTTTAGCTGGTGCTATTACAATATTATTAACTGACCGTAACTTAAACACTTCTTTTTTTGACCCTACTGGTGGGGGAGACCCAATTTTATATCAACATTTATTC

>OTU_22

TCTATCAGGTAACATTGCTCATGCCGGCCCATCTGTAGATCTAGCAATTTTTTCTCTCCATTTAGCGGGAATCTCATCTATTTTAGGAGCACTTAATTTTATTACTACAGTTATTAACATACGATGAACAGCTTTACGTCTAGAGCGTGTTCCATTATTTGTATGAGCAGTACTAATCACAGCAGTTTTACTTCTCCTTTCTCTTCCTGTTCTTGCTGGAGCTATTACTATACTTTTAACTGATCGAAATATTAATACAGCTTTCTTTGATCCCGCTGGAGGTGGAGACCCTATTTTATATCAACATCTATTC

>OTU_23

TTTATCCGGGAATGTTGCACACGCCGGCCCAGCAGTAGATTTAACTATTTTATCCCTTCATCTAGCTGGGGTATCATCTCTTCTAGGTGCAATTAACTTTACAACTACAATTATAAATAGACGTATAGAGGGAATACCTTCAGAAAAAATGCCTTTATTTATTTGATCTGTGTTAGTGACAGTAGGACTATTAATCTTAGCATTACCTGTTCTAGCAGGAGCATTAACAATATTAATTTTAGACCGTAATTGTAATACATCCTTTTTCGAGCCCACAGGAGGAGGGGATCCTATTCTATTTCAACACTTATTC

>OTU_24

TGTCTTTAAACCTGGCCCACGCGGGTATGTCTGTGGATTTTGCAATTTTCTCTCTTCACTTGGCGGGTATTTCGTCTCTTCTAGGGGCCGTAAACTTTATTAGAACGTTAGGGAACCTGCGTTCTCTGGGGTTAATAATGGACCGCATGCCACTTTTTGCCTGGGCTGTGCTAGTCACAGCCATTTTATTACTATTATCCCTCCCCGTGCTAGCGGGGGCCATTACCATGCTTCTGACGGATCGAAACCTTAATACGTCTTTTTATGACGTAAGAGGGGGAGGGGACCCGGTTCTCTACCAGCACCTGTTT

>OTU_25

ACTCGCAGCAGCTATTGCCCATGCTGGGGCTTCCGTAGATCTTGGTATCTTTTCTCTTCACCTTGCAGGAGTTTCTTCGATCTTAGGGGCTGTCAATTTTATAACCACAGTTATTAATATACGGTCTTATGGTATAACAATAGATCAAATACCCCTTTTTGTATGAGCTGTATTTATTACTGCCATTCTTTTACTCTTATCTCTACCTGTTTTAGCAGGAGCTATCACTATACTATTAACAGATCGTAATTTAAATACATCATTCTTCGATCCTGCAGGAGGAGGTGATCCAGTCTTATATCAACACTTATTC

>OTU_26

ATTGTCCAGAGGTGTGGCACACAGAGGGGGAGCTGTAGATTTAGGTATTTTCTCTCTACACTTAGCGGGAATTTCCTCTATTTTAGGCGCAGTAAATTTTATTACAACGGTTTTGAATATGCGAACTGCTGGGCTGAGATATGACTTGGTTCCTTTGTTTGTGTGGTCTGTGCTTATTACTGCTGTTCTCTTATTACTATCTTTGCCTGTACTAGCGGGAGCAATCACAATGCTATTGACGGATCGAAATTTGAATACAACTTTTTTTGATCCTAGCGGGGGAGGAGATCCTATCCTCTATCAGCACTTATTC

>OTU_27

TCTATCAGGTAATATTGCTCAGAGGGGTCCGAGAGTAGATATGGCAATATTTTCATTACATTTAGCTGGTGTTAGGTCTATATTAGGATCTATAAAATTCATTACTACTATGGTAAAAGCCAAGGTACAAGTTACATGAGGACAATTACCTTTGTTTTTATGAGCAGTAATGGTAACAGCCTATATGTTAGTATTATCATTACCTGTCTTGGCAGGAGGTCTAACAATGTTGTTAACAGACCGAAAATTTAACACTTCATTTTTCGATCCAGGAGGAGGAGGAGATCCCATCCTATTTCAACATATTTTT

>OTU_28

TATCAGGAAACATTGCACACAGAGGGGCTTCAGTGGATTTTGCTATTTTCTCGTTACATTTAGCTGGTGTTAGATCAATCTTAGGTGCTGTTAATTTTATTTCTACTATCGCTAATTTACGCTCATTTGGTATAATTCTAGATCGAATACCTCTTTTTTCTTGAGCAGTACTAATTACTGCAGTATTACTTCTATTATCACTTCCTGTACTAGCAGGGGCTATTACTATATTATTAACAGATCGAAACCTTAATTCATCTTTTTATGATGCTGGAGGTGGGGGAGACCCCGTTTTATATCAACATCTTTTT

>OTU_29

ATTAAGAGCAACCATCGCACACTCAGGGGGCTCAGTAGATTTAGCCATTTTTTCCCTTCATTTAGCAGGGGCCTCCTCAATTTTAGGAGCCATTAATTTTATCTCAACAGTCCTTAATATACGAGCCCCTGGAATAACCATAGACCGTGTTCCACTTTTTGTGTGGTCTGTTTTTATCACTGCAATCCTCTTATTATTGTCTTTACCAGTCCTTGCAGGAGCTATTACAATATTGTTAACAGACCGCAACTTAAACACCTCTTTCTTTGACCCTTTAGGAGGGGGAGACCCAATTCTTTACCAACACCTATTT

>OTU_30

CCTAGCTTCAGCTATTGCTCACGCCGGTGCTTCTGTTGATTTAGGGATTTTCTCTCTTCACTTAGCAGGTGTTTCATCAATCTTAGGGGCCGTTAATTTTATTACTACCGTTATCAACATACGATCCTATGGTATAATATTAGACCAAATACCACTATTTGTATGATCTGTATTTATTACGGCCATTCTCTTACTATTATCCCTACCAGTCCTAGCAGGAGCTATTACTATGCTACTCACTGATCGTAATTTAAATACTTCATTTTTTGATCCAGCTGGTGGTGGTGACCCTGTTTTATATCAACACTTATTT

>OTU_31

TTTATCAGGCAATGTCGCCCATGCAGGACCAGCAGTAGATCTCACGATCTTATCTCTTCATCTTGCAGGGGTATCCTCTCTTCTTGGTGCTATTAATTTTACAACTACAATTATTAATAGACGACTAGAGGGGATGCCAACAGAAAAAATACCTTTATTCATTTGATCTGTTCTAATTACCGTAGGACTCTTAATCCTTGCTCTTCCCGTCCTCGCAGGAGCTTTAACTATGTTAATTATAGACCGAAACTGCAATACATCCTTTTTCGAACCTATAGGAGGGGGAGATCCCATTCTCTTCCAGCATCTTTTC

>OTU_32

ATTAAGAAGAAACACTGGGCATTCAGGAATATCGGTAGATTTAACTATTTTTTCTTTGCATTTGGCGGGGATTTCATCTTTATTAGGAGCAGTAAATTTTATTAGAACCTTAGCTAATTTACGATGTTTAAGAATAAATTTAGATCGAATACCGTTGTTTCCCTGATCTGTTTTAATTACAGCAATTCTTTTATTGCTTTCTTTACCTGTATTAGCTGGGGCAATTACAATATTGTTAACGGATCGTAATTTAAACACTTCATTTTATGATGTTGGGGGAGGAGGGGACCCAGTGTTATACCAGCATTTGTTT

>OTU_33

TCTATCTAGGAATATTGCTCACGCAGGAAGTTCAGTAGATTTTGCTATTTTTTCATTGCATTTAGCAGGGGTAAGTTCAATTTTAGGTGCGGTTAATTTTATTAGAACATTAGGAAATTTACGAGTGTTTGGAATATTATTAGACCGAATACCTTTATTTGCATGGGCGGTATTAATTACAGCAGTTTTACTATTATTATCTCTACCTGTCTTAGCTGGGGCTATTACTATATTATTAACAGATCGAAATTTAAATACAACTTTTTATGATGTTGGGGGTGGTGGGGATCCTATTTTATATCAGCATCTATTT

>OTU_34

ACTCAGCAACTCTACCTTCCATAGGGGGCCATCGGTAGATTTTGCAATTTTTAGTCTTCACCTCGCAGGGATTTCTTCTTTACTGGGAGCAGTAAACTTTATCACTACAATTATAAACCTGCGGACTATCGGAATGCTACTTGACTTGATGCCCATATTCCCTTGGGCAGTGCTAGTGACGGCGATCTTGTTACTTCTATCTCTACCGGTGTTGGCGGGCGCGATTACAATGCTTCTCACAGATCGAAACATTAATAGAGTTTTTTATGACTCGATAGGAGGGGGGGACCCTATCCTGTACCAGCATCTATTC

>OTU_35

TCTATCTAGTAATATCGCCCATTCAGGTCCTTCTGTTGATTTAGCTATTTTTTCATTACACTTGGCTGGTGTATCTTCTATTTTAGCTTCTATTAATTTCATTACAACTGTAATAAATATACGTTCTTCTGGATTACGTTTAGAGCGGGTTCCTTTATTTGTTTGATCTGTAGCTATTACTGCTTTACTACTATTATTGTCTTTACCTGTGTTAGCAGGGGCGATTACTATACTTCTGACCGATCGTAATTTAAATACTTCTTTTTTTGATCCAGCTGGGGGAGGAGATCCTATTTTATATCAGCATTTGTTC

>OTU_36

CTTATCAGGCAACTTAGCACACGCTGGGGCCTCCGTAGACCTAACAATCTTCTCCCTTCATCTAGCCGGGATTTCCTCAATTCTTGGTGCAATTAACTTCATTACAACTATCATTAATATGAAACCCCCTGCCATCTCTCAGTACCAAACCCCCCTCTTTGTTTGAGCTGTTCTTATTACAGCCGTACTACTACTTTTATCTCTACCAGTGCTTGCTGCGGGGATTACAATGCTACTGACAGATCGAAATCTAAACACCACCTTTTTTGACCCCGCCGGAGGTGGAGACCCCATCCTGTATCAACACCTCTTC

>OTU_37

ACTATCAGGAATCCAAAGCCACTCAGGTGGATCAGTTGACCTGGCTATCTTCAGCCTACACCTATCAGGTATCAGTTCAATGTTAGGAGCGATGAACTTCATCACTACAATCCTAAACATGCGACACCCTGGTATGTCAATGCACAAACTTCCTCTATTCTGTTGGGCTATCTTCATTACAGCTATTCTACTTCTACTATCACTACCAGTGCTAGCAGGTGGAATTACAATGCTGCTAACAGATCGAAACTTCAACACATCATTCTACGACCCAGCTGGAGGAGGTGACCCAATCCTATTCCAACACCTATTC

>OTU_38

TTTAAGAAGATCTATCGCCCATAGAGGAGGAGCTGTGGACCTTGCTATTTTTTCACTACACCTGGCAGGTGCTTCTTCTATCTTAGGGGCTATTAATTTTATTTCTACTGTAATTAATATACGATCCACGAATATATATATAAGACGAGTGCCTTTATTTGTTTGATCAGTCTTTATCACTGCTATTTTACTACTTTTATCTCTCCCTGTCTTAGCAGGCGCTATCACCATGCTACTAACAGATCGAAATATCAATACATCTTTTTTTGACCCTTTAGGAGGGGGAGACCCTATCCTATACCAACATTTATTT

>OTU_39

TTTATCTGGAAATTTAGCTCATGCAGGTCCATCAGTTGACCTCGGGATTTTCTCTTTACATCTTGCCGGGATTTCTTCTATTCTTGGAGCCATTAATTTTATTTCTACTGTTCGAAACATACGAGTTCAAGGAATTCAAACTGGTCGAATGCCTTTATTTGTTTGAGCCACTTTAATTACCGTAATTCTCCTATTGCTTTCTCTCCCCGTTTTAGCTGCTGCTATTACTATACTTTTAACTGATCGTAATTTTAACACTTCATTTTTTGACCCTGCTGGAGGCGGAGATCCAATTTTATACCAGCATTTATTT

>OTU_40

TCTTTCTTCGAATATTGCTCACGCTGGCCCCTCTGTAGACCTTGCTATTTTTTCACTTCACTTAGCCGGTGTCTCTTCTATTTTAGGCGCGTTAAATTTTATTACAACTATTATTAATATGCGCATAAGAGGGATAAAATTTGAAAATATCTCTTTATTTTTATGAGCCGTATTTATTACTGCAATTTTACTTTTACTTTCGTTACCTGTCTTAGCGGGCGCAATTACAATACTTCTAACCGATCGCAATTTAAATACTTCTTTCTTCGACCCCGCAGGGGGAGGAGACCCTATTTTATACCAGCACCTCTTT

>OTU_41

TCTTGCGGGTAATATCGCTCATGCAGGTCCCTCCGTTGATCTAGCTATCTTTTCCCTCCATATTGCAGGTGTTTCATCCATCCTTGGCGCCTTAAATTTCATCACCACGGTTATTAATATACGCTATAAAGGGCTACGCCTTGAACGTGTTCCCTTATTTGTCTGAGCCGCAAAAATTACAGCCATCCTTCTTCTTTTATCCCTTCCTGTCTTAGCCGGAGCTATTACTATATTACTAACTGATCGTAATCTAAACACCGCTTTCTTTGACCCAGCAGGCGGCGGTGACCCCGTTCTCTACCAACACCTATTC

>OTU_42

TCTATCTTCTAATGATAGAACACATACTATAGAATATTCTATAGCTTCTATACATATTCTATCATTATCTTCTTTATTATCTGGATTAAATACTTTATCTAGTATAATAAGATATATTAATAAATTATATAAAGATATAGATTTATTTTCTATATCTCAGATAGTTACAGCTTTTTTATTAGTATTAACTACTCCAAGTCTTTCTCTATCTGTTACAGGAATATTATTAGACTCTTCTATAGGATTTAACTCTTATGAGTCCTCTGGATATGGAGATCCAGAATATTATCAAATATTATTC

>OTU_43

TCACAGGAGGGCCCGCGCTGGTCTGGTGCCTTGCTAATGGGTAGAGGAGGCTTCCCTGAGATTCACGAAACAGTATCTAGCACAGAATCACGGGATGGTCTTGTCATGATGTTTTCCACAGGTGCTCATGTTCCCCAGTGCACGTGTGAATGGTGTGCACGTTCTGCGCAGATCTTAATTTAAGGGAAGATTTTTCATCCTCATCGGAAAACAGTTAAAACTGC

>OTU_44

ACTTTCCGGGAACGTTGCCCATGCCGGCCCAGCAGTAGATTTAACAATTCTATCTCTACATCTCGCTGGTGTGTCATCTTTAATAGGAGCAATTAACTTTACAACAACTATTCTTAATAGACGAATAGAAGGTATACCTACAGAAAAAATGCCTTTATTTATTTGATCTGTCTTAGTTACTGTGGGTCTTCTCATTTTAGCCCTCCCAGTTCTTGCTGGAGCCCTTACTATACTTATTATAGACCGTAATTGTAACACCTCTTTCTTCGAACCTACAGGAGGAGGAGACCCAATTCTTTTTCAACATCTATTT

>OTU_45

ATTAAGAAGAAATATTGCTCATGCTGGAGCGTCAGTAGATTTTGCAATTTTTTCCTTACACTTAGCTGGGGTTTCCTCTTTACTGGGAGCAGTTAATTTTATTAGAACTTTAGGAAATTTGCGATCGTTAGGGATAAGATTAGACACTGTGCCTTTATTTGCTTGGGCTGTTTTTATTACAGCTGTTCTTCTTTTATTATCATTACCAGTTTTAGCTGGAGCTATTACTATACTTTTAACTGATCGTAATTTAAATACAGCTTTTTACGATGTTGCGGGAGGAGGAGACCCAGTGCTCTATCAACATTTGTTT

>OTU_46

ATTATCTGGTAACGTAGCTCACGCAGGTCCAGCAGTTGATTTAACAATTTTATCTCTTCATCTTGCTGGTGTTTCGTCTCTAATAGGGGCAATTAATTTCACGACAACTATTCTTAATAGACGAATAGAGGGGATGCCAACAGAAAAAATACCTTTATTTATCTGGTCTGTCCTCGTTACTGTAGGCCTATTAATTCTAGCTCTTCCAGTTCTTGCCGGAGCTCTTACTATACTTATTATAGACCGAAATTGCAATACTTCTTTCTTTGAGCCAACAGGAGGAGGGGACCCCATTCTTTTCCAACACTTATTC

>OTU_47

TTTATCTAATAATGTCGCACATGCCGGACCTGCAGTAGATTTAACTATTCTATCTCTCCATCTTGCAGGTGTATCCTCCCTAATAGGAGCAATTAATTTTACAACAACCATCGCAAATGCCCGACTTGAAGGCATACCAACAGAAAAAATACCCCTTTTCATTTGATCAGTTCTTATTACAGTCATTCTCTTAATTCTTGCCCTACCTGTCTTAGCAGGTGCTCTAACTATATTAATTATAGACCGTAATTGCAACACTTCCTTCTTTGAGCCAACAGGAGGAGGAGACCCTATTCTATTCCAACACCTGTTT

>OTU_48

TCTATCAGGTAACGTAGCCCATGCAGGTCCCGCAGTAGATCTAACCATTCTCTCCCTTCACATTGCAGGTGTTTCGTCTTTAATAGGAGCAATTAACTTTACAACAACTATTGCTAATAGACGATTAGAAGGTATACCAACAGAAAAAATACCATTATTCATTTGATCTGTTCTAGTAACAGTGGGTTTATTAATTTTAGCTCTTCCCGTCTTAGCTGGAGCTTTAACTATATTAATTATAGACCGAAACTGTAATACATCATTCTTTGAGCCTATAGGAGGAGGAGATCCTATTTTATTCCAACACCTATTT

>OTU_49

CTTATCTTCAAATATTTCACATTTGGGTGGTGCCGTAGATTTAAGAATTTTTTCACTTCATTTAGCTGGAGCTTCATCTATTTTAGGAGCTATTAATTTTATTACAACTATTATTAATATAAAAGCACCTTATATAGAATATGACTCTTTACCATTGTTCGTATGATCAGTATTTATTACAGCAGTACTACTACTTTTATCATTACCAGTATTAGCAGGAGCTATTACAATACTATTAACGGACCGAAATTTAAATACTTCATTTTTTGATCCTTTGGGTGGTGGAGATCCTATTCTTTATCAACACCTATTT

>OTU_50

TTTATCTACTTTCTCATATCATGGAATGTGTATGGACTTGGCTATTTTGAGGCTTCACTTAGCTGGTATTAGATCAATTTTTAGGTCAATTAACTTTATGGTGACAATTACTAATATACGATCAGTAGATGGCCATTTATTGGCTTTGTTTCCTTGATCTATTAAAGTGACTTCATTTTTATTACTTACCACTCTTCCAGTATTGGCCGGCGGGTTGACTATGTTACTGACGGATCGGCATTTTAATACTTCTTTCTTCGATCCAGTAGGAGGTGGTGATCCGGTCTTATTTCAGCATTTATTC

>OTU_51

TCTTTCAGGAAATCTTGCTCATGCCGGACCATCTGTAGATCTTGCCATCTTTTCTCTTCACCTAGCAGGAGTTTCTTCCATTTTAGGTGCCCTAAACTTTATTACTACTGTAATTAATATACGATCAAAAGGCCTACGTCTAGAACGTATCCCACTTTTTGTATGAGCTGTTGTAATTACCGCTGTTCTTCTTCTTCTCTCTCTTCCTGTCTTAGCAGGAGCTATTACAATACTCCTAACTGACCGAAATCTCAATACCTCATTCTTCGATCCAGCAGGAGGAGGAGACCCAATTCTCTATCAACACCTTTTC

>OTU_52

TCTTTCTTCTAATATAGCTCAAAGAGGGCCTAGTGTGGATTTAGCTATTTTTTCTCTACACTTAGCAGGAGTAAGATCAATATTAGGATCAATAAAATTTATAACTACTATGGTTAATGCAAAGCTACAGGTAACCTGGGGCCAACTCCCTTTATTCTTGTGAGCGGTGATGGTGACAGCATATATGCTTGTATTATCCCTACCTGTTTTGGCGGGAGGTTTAACTATGTTATTAACGGACCGCAATTTCAAAACTACATTTTTTGACCCAGGTGGTGGAGGGGATCCTATTTTATTTCAACATATATTT

>OTU_53

CCTATCTGGAAATGTCGCACATGCAGGCCCTGCAGTAGATCTAACAATTTTATCTCTCCATCTAGCAGGTGTGTCATCCCTCTTAGGGGCTATTAACTTTACAACCACTATTCTTAATAGACGAATAGAAGGTATACCTACTGAAAAAATACCACTCTTTATTTGATCAGTTCTAATTACTGTAATTCTTCTAATTCTAGCCCTCCCCGTTCTAGCAGGTGCTCTAACTATACTTATTATAGACCGAAACTGTAATACATCCTTCTTTGAGCCTATAGGAGGAGGAGACCCAATTCTATTCCAGCACCTCTTT

>OTU_54

CTTGAGAGGAAATGTATCCCACAGCGGTGCTGCTGTTGACTTTGCGATTTTTTCTTTACACCTAGCCGGAGTATCCTCACTGCTAGGAGCTATCAATTTTATCACCACTATTGTAAACCTGCGAGCACTCGGGATGTTCCTAGACCGGATACCAATATTCCCCTGGGCAGTGCTTATCACTGCTATCCTTTTACTGCTCTCACTACCTGTGCTTGCTGGAGCCATTACTATGCTATTAACAGACCGTAATTTTAACTCTTCCTTCTATGACCCCAGGGGAGGGGGTGACCCGGTCCTATACCAGCACCTATTT

>OTU_55

TTTATCTGGAAATCTTGCTCATGCTGGTCCTTCTGTCGACCTTGCAATTTTTTCCTTACATTTAGCAGGAGTTTCCTCAATTTTAGGAGCTTTAAATTTTATTACTACTGTGGTTAATATACGTTCTAAAGGACTCCGACTCGAACGGATTCCTTTATTTGTTTGAGCCGTAGTAATTACTGCTGTTCTTCTTCTTCTTTCTCTCCCTGTTTTAGCTGGAGCTATCACAATGCTTCTAACAGATCGAAATTTAAACACATCATTCTTTGATCCTGCCGGAGGAGGAGATCCTATTCTTTACCAACACTTATTT

>OTU_56

ATTAAGAAGTTCTATCGCCCATAGGGGAGGAGCTGTTGACCTTGCTATTTTTTCACTTCATTTAGCTGGTGCCTCTTCTATTTTAGGGGCAATCAACTTTATCTCTACCGTTATTAATATACGATCTACTAATATATACATGAGACGAGTTCCTTTATTTGTGTGATCTGTCTTTATTACCGCTATTTTACTACTACTGTCTCTACCTGTTTTAGCTGGTGCTATCACTATATTATTAACAGACCGAAATATTAATACATCCTTTTTTGATCCTTTAGGAGGAGGGGACCCTATCCTATACCAACATTTATTT

>OTU_57

TCTTGCAGCCGGAATTGCTCATGCCGGAGCTTCAGTTGATATAGGAATCTTCTCTCTACATTTAGCAGGAGTATCCTCTATTTTAGGGGCCGTAAATTTTATAACAACAGTGATTAACATACGGGCATCTGGAATAACATTAGACCGAATACCTTTATTCGTATGATCTGTATTTATTACTGCACTATTATTACTTTTATCACTACCAGTTCTAGCTGGAGCTATTACAATGTTACTAACAGATCGAAATCTTAATACATCTTTCTTCGACCCTGCTGGGGGTGGAGATCCCATTTTATATCAACATTTATTT

>OTU_58

TCTAACATCTATACAAGCTCACAGAGGCCCCTCTGTTGACCTAGCTATCTTCTCATTACACCTAGCCGGTATCTCTTCTATCTTAGGTTCTATCAACTTTATTTCAACTATTATAAACATAGCCTCTCACAATAACTCCACCAATCAAATAACACTATATTGTTTCTCAATCTTTATTACTACTATTCTACTACTTCTATCCTTACCAGTTCTAGCCGGAGCTATTACTATACTACTAACAGACCGAAATATTAACACATCATTCTTTGACCCAACAGGAGGAGGAGACCCTGTTCTTTACCAACACTTATTT

>OTU_59

TTTATCTTCATATACTGGTCATAGCGGTCCGGCAGTTGATATGTCTTTATTTGCTTTACATTTAGCCGGTGCTTCATCCATTGGAGGTTCTATTAACTTTTTAACTAGAATTAAAAATCTTCCTATAAAAGAGATGCGTGGAGAGCGTATAGTATTATTTATTTGGTCTATGGTAGTAACTGCTGTTCTTTTGTTAGTATCGCTTCCTGTACTAGCTGGTGGAATTACTATATTAATTTTTGATCGGCACTTTAATACCTCTTTTTATGATCCGTCTGGTGGAGGGGATCCTGTTCTTTATCAGCATTTATTT

>OTU_60

TCTAGCAAGAAACCTGGCCCATGCGGGCCCTTCTGTTGACCTTGCAATCTTTTCCCTTCATTTAGCAGGAGTATCTTCAATTTTAGGGGCCTTAAACTTTATTACTACTATCATTAACATACGATGAAAAGGACTCCGCCTAGAGCGAATTCCGTTATTTGTATGAGCGGTAGTTATCACAGCAGTCCTACTTCTTCTATCTCTTCCTGTTTTAGCAGGAGCAATTACAATGCTTCTAACCGACCGTAATCTAAACACATCTTTCTTTGACCCTGCAGGAGGTGGAGACCCTATTCTCTACCAACACCTTTTC

>OTU_61

CCTATCTAGTAATATTGCTCATGCAGGTAGATCTGTTGATTTCGCAATTTTTTCTTTGCACTTAGCGGGTGTAAGATCTATTCTTGGAGCTGTAAATTTTATTAGAACTGTTGGGAATCTACGAGTATTTGGAATAATCTTGGATCGAATACCTTTGTTCGCATGAGCTGTTTTAATTACAGCTATTTTATTGCTTTTATCTCTGCCTGTTTTAGCAGGGGCTATTACAATACTTCTTACAGATCGAAACCTTAACTCTTCATTCTATGATGCAAGAGGGGGTGGTGATCCAATTCTTTACCAACATTTATTT

>OTU_62

TTTGTCTAGAAATATTGCCCACGCTGGTGCGTCAGTTGATTTTGCTATTTTTTCTCTTCATTTAGCAGGGATCTCCTCACTGTTAGGGGCTGTGAATTTTATCAGAACCCTGGGGAATCTTCGTGTCTTTGGGCTTCATTCTGACCGAATGCCGCTGTTTGCCTGGTCAGTTCTTGTAACGGCTATTCTTTTGCTGTTAAGACTTCCGGTATTAGCAGGTGCTATTACGATGCTCTTAACTGACCGAAACTTAAATACTTCATTCTATGATGCGAGAGGAGGGGGAGACCCGGTTCTCTACCAGCATTTGTTC

>OTU_63

TTTGTCAGGTGGGGATTATTCTGGGTGGGGCACTGATTTTTTGATGTTTTCTTTGCATTTGGCAGGTGTTTCTAGGGTTTTTGGTTCTTTGAAATTTATTTGTACCATTGTGAGTGCTTTGGGGGATAAGGCGGCAACGCGTTGTTCTATTATTGTGTGGGCTTATTTGTTTACCTCAATATTGCTTATTTTGTCTCTCCCTGTGTTGGCAGCGGCAATTACTATGTTGTTGTTTGATCGTAATTTTAGTTCTTCCTTTTTTGATCCGTTGGGAGGGGGGGACCCTGTTCTTTTTCAACACATGTTT

>OTU_64

TCTTTCTTCAAATATTGCCCATGCAGGACCCTCTGTAGATCTTGCCATTTTTTCGCTCCACCTTGCCGGTGTTTCTTCTATCCTGGGCGCATTAAACTTTATTACAACCATTATTAATATACGGATAGCAGGCATGAAATTTGAAAATATTTCATTATTTTTATGGGCTGTTTTTATTACTGCAATTTTACTTTTACTTTCACTTCCTGTATTAGCCGGCGCAATTACGATACTTTTAACTGATCGCAACTTGAATACTTCCTTCTTTGATCCTGCGGGCGGGGGAGATCCTATTTTATATCAACATCTCTTC

>OTU_65

TTTAAGTTCTAATTTAGGACATAGAGGCTGTTCTGTAGACTTGGCTATTTTCTCTTTGCACTTGGCAGGAGTTTCTTCTTTAATAGGGGCGGTAAACTTTATCACTACAATTACTAATCTTCGAGCTTTTGGAATACAAACTGAGTCTACTCCTCTTTTTGTCTGATCAATTTATATTACTGCAATTCTTCTATTATTATCGCTTCCGGTCTTAGCAGGGGCTATCACTATGTTATTAACAGACCGAAACTTAAATAGTTCATTTTATGACGCAGCTGGAGGGGGAGATCCCATTCTTTACCAGCATTTATTC

>OTU_66

TTTATCCGGGAACGTTGCCCATGCAGGGCCTGCGGTTGATTTAACCATTCTCTCACTACATATTGCCGGTGTTTCCTCATTGCTTGGCGCCATTAATTTCACAACAACTATTATGAACAGACGTATGGAAGGCATGCCTACCGAAAAAATACCCCTATTTATTTGATCTGTTCTTGTAACAGTGGGCCTTCTTATTCTAGCTCTCCCAGTACTTGCAGGAGCCCTAACTATATTAATTCTAGACCGTAATTGTAATACCTCTTTCTTTGAACCGACAGGAGGGGGCGACCCTATCTTATTCCAGCATTTATTC

>OTU_67

TCTAGCAGGGAATTTAGCTCATGCTGGTGGTTCTGTAGATCTTGCAATTTTTTCTTTACATTTAGCAGGTGTTTCTTCTATTTTAGGAGCTGTTAATTTTATTACTACAATTATTAATATACGTTGACGTGGTATACAATTTGAACGGCTTCCATTGTTTGTATGATCAGTTAAAATTACAGCAATTCTTCTCTTACTATCTTTACCTGTTTTAGCAGGTGCTATTACAATATTATTAACGGACCGGAACTTTAATACGGCATTTTTTGACCCAGCAGGAGGTGGGGATCCTATTTTGTACCAACACTTGTTT

>OTU_68

ACTTTCGAGAAATATCGCCCATGCGGGCCCATCTGTCGACTTAGCAATCTTCTCACTTCATTTAGCAGGAGTATCATCAATCCTAGGGGCAGTTAATTTTATTACTACTGTAATCAATATACGATGAAGTGGACTTCGACTAGAGCGAGTCCCACTATTTGTATGGGGTGTTAAAATTACTGCAATTCTGCTTTTATTATCACTCCCTGTACTAGCTGGTGCTATCACCATACTATTAACAGACCGCAACCTAAATACATCCTTCTTTGATCCGGCTGGGGGCGGCGATCCAGTACTATATCAACATTTATTC

>OTU_69

TTTAGCAGGTAATATTGCTCATTCTGGAGCTTCTGTTGATTTTACTATTTTTTCTTTACATTTAGCGGGGGCGTCTTCAATTTTAGGGGCTATTAATTTTTTATCAACAGTTATTAACATACGGCCTTCTTCTATAACTTTTGATCGTATTCCTTTATTTGTTTGAAGTGTTTTTATTACAGTTATTCTTCTATTGCTTTCTCTTCCAGTACTAGCAGGAGCAATCACAATACTTTTAACTGATCGAAACTTAAATACGTCATTTTTTGACCCTATTGGAGGGGGTGACCCTATTTTATACCAACATCTTTTT

>OTU_70

TCTATCAAGAAATCTTGCTCATGCTGGCCCATCAGTAGACTTAGCCATTTTTTCTCTTCATTTAGCCGGAGTTTCCTCTATTCTCGGAGCACTAAACTTTATTACAACAATTATCAACATACGATCAAAAGGCCTTCGAGCTGAACGAATCCCTTTATTCGTATGAGCAGTTCTAACAACTGCTATCCTCCTTCTTCTTTCCCTTCCTGTTCTGGCTGGAGCAATTACTATGCTTCTCACAGACCGTAATCTAAATACCTCATTCTTTGATCCAGCTGGCGGTGGAGATCCTATTCTATACCAACACCTATTT

>OTU_71

TTTATCGGGAAATGTAACTCATGGAGGAGGTTCAGTTGATTATGGGATCTTCTCTCTCCATTTGGCGGGTGTTTCCTCTATTTTGGGGGCTATTAATTTTTTGGCTACTATGGTTAATATACGTCCTGAAATTATGGAGTTGAAACGGGTTACTTTATTTGTTTGGTCTATTGGAATTACGGCCTTTTTACTGGTTGTGGCTATACCTGTTTTGGCTGGTGCTATTACAATATTGTTAACTGATCGAAATTTTAATACTTCTTTTTTTGATCCATCGGGTGGAGGTGACCCTATTTTGTTTGTGCATTTGTTT

>OTU_72

TTTATCTAATAATGTAGCACATGCCGGACCTGCAGTAGATTTAACTATTTTATCTCTTCATCTTGCAGGTGTATCCTCCCTAATAGGAGCAATTAATTTTACGACAACCATCGCAAATGCCCGGCTTGAAGGCATGCCAACAGAAAAAATACCCCTTTTCATTTGGTCAGTTCTTATTACAGTTATTCTCTTAATTCTTGCTCTACCTGTTCTGGCAGGTGCTCTAACTATATTAATTATAGACCGTAATTGCAACACTTCCTTCTTTGAGCCAACAGGAGGAGGGGACCCTATTCTATTTCAACACCTATTT

>OTU_73

GGAACCGCACCGACGAAGAGGGAAATGTTTACTCGAGTGATCACGAGGGGGAGGAGGGGGACAAGAGGGAGGACGAGGAGGAGATCGACTTGGAGAACATCGACACGGAAAATATTGAGAGCAAGGACGACTTGGACGACCAGGACGACCTGCATTCAGACATTAAACTAGACGGCAGGAGTGACTCTGAGATTTCTGACGGCTATGAGGATTTACAAGGGCCCGATCAGAGGTTTCTAAAAGCGGTGGGGAAAGAGGGCAAAGACGTGGAAAGAGGAGCGGAGCACTTCCACAGCCACCACCACCACCACCATCATCA

>OTU_74

TTTGTCAACTTTTTCGTACCATGGAATGTGCATAGACTTGGCAATTCTAAGGCTTCATTTAGCCGGGATTAGTTCAATTTTTAGATCAATTAATTTTATGGTCACAATTACAAATATGCGATCAGTAGACGGACATCTACTGGCTTTATTCCCGTGATCAATTAAAGTAACCTCATTTTTATTATTGACCACTCTGCCTGTACTAGCCGGAGGCTTAACTATACTACTTACAGATCGTCATTTTAATACATCTTTCTTTGATCCTGTAGGCGGTGGAGATCCTGTATTATTTCAGCATTTGTTC

>OTU_75

TTTATCAAGTAACATTGCTCATTCTGGTGCTTCAGTTGACTTATCAATTTTCTCTTTACATTTAGCGGGTGCTTCGTCAATTTTAGGTGCCATTAATTTTATGTCTACAGTTATTAACATACGAGCTGAAACACTGACATTTGATCGACTTCCATTATTTGTCTGAAGAGTATTTATTACTGTAATTCTTTTACTTTTATCACTTCCAGTACTAGCAGGAGCTATTACTATGTTACTAACAGATCGAAATCTGAATACCTCATTTTTTGATCCAACAGGAGGTGGAGATCCAATCTTATACCAACATCTATTT

>OTU_76

ATTGGCTGGAAATCTGGCTCATGCTGGTGGTTCAGTAGACCTTGCAATTTTTTCTTTACACTTAGCTGGGGTTTCTTCTATTTTAGGGGCTGTGAACTTTATTACAACCATTATTAACATACGTTGACGAGGTATACAGTTTGAACGACTTCCTCTTTTTGTGTGATCTGTGAAAATTACAGCAATCCTTCTTCTACTATCTCTTCCAGTGCTAGCAGGGGCTATTACGATACTGTTAACAGATCGAAATTTTAATACTGCATTCTTTGATCCTGCAGGAGGAGGGGATCCTATTTTATATCAGCATTTATTT

>OTU_77

CCTTTCTAGAAATATCGCGCACGCGGGCAGATCAGTGGATTTTGCAATTTTCTCCTTGCATTTAGCTGGTGTCAGCTCTATCTTAGGGGCGGTAAATTTTATTAGAACTGTAGGAAACCTGCGGGTGTTTGGGATAATTTTAGACCGGATGCCTCTATTCGCGTGGGCTGTTTTAATTACTGCTATTCTATTATTACTATCTCTACCGGTGCTGGCGGGGGCCATTACTATACTCCTCACGGACCGAAACTTAAACTCTTCTTTCTATGACGCTAGAGGCGGCGGGGATCCCATTTTGTACCAGCACCTGTTT

>OTU_78

TCTCAGAAGAAATTTAGGACATTCTGGAATAAGGGTAGATTTAACCATCTTTTCCCTGCATTTAGCCGGAATTTCCTCTTTATTGGGGGCTGTTAATTTTATTAGAACTTTAGCAAACTTGCGCTCACTAAGAATATCTTTAGATCGTATGCCCTTATTCCCCTGAGCTGTTTTAATTACGGCAATTCTCCTTCTTCTTTCTCTGCCCGTGCTAGCCGGTGCAATTACTATGCTTCTAACCGATCGTAATCTTAACACTACTTTTTATGACCCTAGAGGGGGGGGGGATCCAGTTTTATACCAACACTTATTC

>OTU_79

TTTGTCAAGAAATATTGCACATTCTGGTGCTTCTGTAGATCTCTCAATTTTCTCGCTTCACCTAGCCGGAGCATCTTCAATTTTAGGAGCAATTAATTTTATATCAACAGTTATTAATATACGATCTGAAACTTTAACTTTTGATCGTCTACCTTTATTTGTTTGAAGAGTCTTTATTACTGTAATTCTACTCCTTTTATCATTACCTGTATTAGCAGGTGCTATTACTATATTATTAACCGACCGAAATTTAAATACTTCTTTCTTTGATCCCACAGGAGGTGGAGATCCAATTTTATACCAACATTTATTC

>OTU_80

TCTGGCCAGGGCTATCGCCCATTCAGGAGCCTCAGTTGATATAGGTATTTTTTCCCTTCATTTAGCAGGGGTGTCCTCAATTTTAGGTGCTATTAACTTTATAACTACTGTTATTAATATACGGTCTTTAGGAATATCCTTAGATCAAATACCTTTATTTGTTTGAGCAGTATTTATTACCGCAGTGCTTCTTCTTTTATCCTTACCGGTCCTAGCAGGTGCAATTACAATACTTCTTACTGACCGTAATTTAAATACCTCATTCTTCGATCCAGCAGGAGGGGGGGATCCAATCCTTTACCAACACTTATTT

>OTU_81

TTTGAGCAGTAATATTGCCCATTCTGGGCCCTCTGTAGACTTTGCCATTTTTTCCCTTCACCTGGCCGGAGTTTCCTCTCTTCTCGGGGCTGTTAACTTTATTAGAACTCTCAGAAACTTGCGAACCTTGGGACTACTCTTAGATCGTCTTCCTCTTTTTGCCTGGGCCGTACTTGTTACCGCCATCTTACTTTTACTATCTCTACCTGTCTTGGCAGGAGCTATTACCATGCTGCTAACAGATCGTAACTTAAACACCTCCTTCTACGACCCTAGAGGAGGGGGGGACCCTGTCTTATACCAGCACCTGTTC

>OTU_82

GCTGGCTGGAAATCTTGCCCACGCTGGAGCATCCGTAGACTTAACCATCTTCTCTCTTCATCTAGCAGGTGTCTCATCAATTCTAGGGGCTATTAATTTTATTACTACTATTATTAATATGAAACCTCCTGCGGTTTCAATGTATCAAATCCCGCTATTCGTCTGAGCTGTTTTAATTACGGCCGTACTTCTTCTTCTCTCTCTCCCCGTCTTAGCTGCTGGTATTACAATGCTTCTAACAGACCGAAACCTAAACACTGCCTTCTTCGACCCTGCAGGGGGAGGAGACCCAATTCTTTACCAACACTTATTC

>OTU_83

TCTCCTCCTCTTTTTTGGAAGGGTGAGTGGAGGGGCACATGCAAAAATGGATTTCTTCCTCCACAGCTGAGGAATTAAAAAAAGAAAGGATACAGGTCTTGTTGACAGAGTAAGAAGTTTTTTCCCTCCAAAATACGGTTGCAGCGTTTTGTATAATTTTGTAGAATTACTTCCTGACCGCCCTGGCAGTGGGTGCAGATGCTTCGCCATTAAGATCCCGGAATGGTTTTGAGGACTTGAAATGCATTTG

>OTU_84

TTTAAGGTCGACCTTAGGTCATTCAGGCCCTTCTGTAGATTTCGCAATTTTTTCTTTACACTTAGCAGGAATTTCTTCTTTGTTAGGAGCAGTAAATTTTATTAGAACTTTAAAAAACACACGGTCTTTTGGTTTGGTTTTAGACCGCATAAGAATATTTCCATGGTCTGTTCTTATCACTGCAATTTTATTATTACTATCTTTACCTGTTTTAGCAGGAGCTATTACAATACTTTTAACTGATCGAAATTTAAATACAACCTTCTACGATCCTAGAGGCGGGGGAGATCCTATTTTATATCAACACTTATTT

>OTU_85

TGTCTTTAAATCTGGCCCACGCGGGCATGTCTGTGGATTTTGCAATTTTCTCTCTTCATTTGGCGGGTATTTCGTCTCTTCTAGGAGCCGTAAACTTTATTAGAACGTTAGGGAACCTGCGTTCTCTGGGGTTAATAATGGACCGCATGCCACTTTTTGCCTGGGCTGTGCTAGTCACAGCCATTTTATTACTCTTATCCCTCCCCGTGCTAGCGGGGGCTATTACCATGCTTCTGACGGATCGAAACCTTAACACGTCTTTCTATGACGTAAGAGGGGGAGGGGACCCAGTTCTCTACCAGCACCTATTT

>OTU_90

TCTTAGTGCTAACCTGGGACACTCAGGACCCTCTGTAGACTTAGCCATTTTTTCTATTCACCTAGCAGGTATTTCTTCTCTACTAGGTGCCGTCAACTTCATTAGAACAATTAAAAATACACGAACATCTGGATTTTCAATAGACCGTATGTGTTTATTCCCTTGATCCATCCTAATTACTGCGGTATTACTTCTATTAGCTCTTCCTGTATTAGCCGGAGCTATTACAATACTTCTAACTGACCGTAACCTTAATACTTCCTTTTACAACCCGGCAGGGGGAGGAGATCCAATTCTTTTTCAACATTTATTT

>OTU_92

TATCTACTGTAGAATTTCACAGAAGGCCTGCAATAGACTTAGCTATTTTGTCCCTGCATCTAGCAGGTTCTGGTTCTTTAATGGGTGCTATTAATTTTTTAACTTCGAACAAAAACCTTCCAGTTGATAAAATAAAAGGTGAGCGTTCAGTTTTGTATGTGTGAAGGATTACTGTTACTGCTTTTTTATTATTATTGTCTCTGCCAGTTTTAGCCGGGGGCATCACTATGTTGTTATTTGATCGAAACTTCAATACCACATTTTTTGACCCTATTGGAGGAGGGGATCCTGTACTTTTTATGCATTTGTTT

>OTU_96

ATTAAGGAGATCTATCGCCCATAGAGGAGGAGCTGTTGACCTCGCTATTTTTTCACTTCATTTAGCTGGTGCATCTTCTATTTTAGGGGCAATCAACTTTATCTCCACCGTTATCAATATACGATCTACTAATATATATATAAGACGAGTTCCTTTATTTGTGTGGTCTGTCTTTATTACCGCTATTTTATTACTTCTATCTCTACCTGTTTTAGCTGGTGCTATCACTATATTATTAACAGACCGAAATATTAATACATCATTTTTTGATCCTTTAGGGGGAGGAGACCCTATCTTATACCAACATTTATTT

>OTU_97

ATTCTCTCCACCTCTCTCCATCTCCCTCTCTCGTGACAGGTTGCTTTGAGACAGAAGGATAAGCTCTTTTTCAGAGAGAGGGAATCGGATTTCCTGCTTTTTGATGAGATAATAAAATATGACTGAGCACAATGGGAGCCTGTGGATGGCTTGCTGGGAGCCATTCACTCCAGCAGTGGGCCCTGTCTTAAGACTGCTTAGACAAACGTTTAGAAAGGTAGCCAGAGGCCTGAGCCCTCAAAGAGCAGCTACTGTTACAAACACGTTGCGTATATGTGTCTTTGCCCGTGTTTTGCTAAACCCTGAGAAATCTCTTGTCTCTCGGTGACTCAGGGTTGGC

>OTU_98

CCTTAGAAGGAACTTGGGACATTCGGGAGCATCAGTAGACCTTACAATTTTTTCTTTACACTTAGCCGGCATCTCATCTCTTTTAGGTGCTATCAACTTCATTAGCACATTAGCTAATCTTCGGTGTTTGGGGATAAAACTTGACCGCATACCTCTTTTCCCTTGATCTGTTTTAATTACTGCAATTCTTTTACTTTTATCGCTTCCTGTTTTAGCTGGTGCAATTACTATGCTACTGACTGATCGTAATCTAAACACTTCTTTTTATGATGCTAGAGGTGGAGGGGACCCAGTTCTTTACCAACACCTATTT

>OTU_101

TTTAGCGAGCAATATTGCCCATGCCAGACCTGCAGTAGATATAGCAATCTTCTCATTACACCTTGCTGGAGCATCTTCCATTGCTGCATCTATTAATTTCATAACCACAATTTATAATATACGAAATGAGGGGTACACGATGGAACGTGTTCCACTGTTTGTGTGGTCTATCTTAATTACGGCTGGACTTCTAGTTCTCGCTCTTCCTGTTCTTGCTGCAGGGATTACAATACTCCTAACTGATCGGAATCTGAACACCACCTTTTTCGATCCAGTAGGGGGAGGAGACCCTGTTCTTTATCAACACCTATTC

>OTU_110

CTTGTCTGGGAATGTTGCACATGCAGGTCCTGCAGTAGATTTAACAATTTTATCTCTCCATCTAGCAGGTGTCTCATCTCTGTTAGGAGCTATTAACTTTACAACTACTATTCTTAATAGACGAATAGAAGGCATACCTACTGAAAAGATACCACTCTTTATTTGATCGGTTCTAATTACTGTAGTTCTTCTAATTCTAGCCCTCCCTGTTCTAGCAGGTGCTCTAACTATACTTATTATAGACCGAAACTGTAATACATCCTTCTTTGAGCCTATAGGAGGAGGAGACCCAATTTTATTCCAACATCTCTTT

>OTU_116

CTTAGCTAGAAATATTGCACACGCAGGACCCTCAGTAGACTTAGCTATTTTCAGGCTCCACCTCGCAGGGGCCTCATCTATTTTAGGAGCTGCAAATTTTATCTCTACGGTAATAAATATGCGGAGAAGAGGATACCGGCTTGAACGAATTCCTCTATTTGTATGAGCAGTAAAAATCACTGCTATTTTACTTCTCCTCTCACTTCCCGTTTTGGCTGCAGGAATTACAATGTTGCTAACAGATCGAAATCTTAACACTACATTCTTTGACCCCTCTGGTGGTGGAGACCCAGTTTTATATCAACACTTATTC

>OTU_138

CTTGGCCGGGAACGGAGCACACGGAGGACCTTCCGTAGACCTTGCTATTTTCTCGCTTCATCTTGCCGGTATTTCATCAATCTTAGGAGCCTTAAACTTCATTACAACCGTTATTAATATACGATGAACCGGGCTACGCCTCGAACGAATCCCATTATTTGTCTGAGCTGTAGTCATCACAGCGGTCCTTCTTCTCCTCTCCCTCCCTGTTCTTGCCGGGGCTATTACAATGCTCCTAACAGACCGAAACCTTAATACCTCATTCTTCGACCCCGCTGGAGGAGGAGACCCTATTCTCTATCAACACCTCTTC

>OTU_147

TCTATCGAGTAACATTGCTCACGCAGGGAGCTCTGTAGACTTTGCTATTTTTTCTCTTCATTTAGCCGGTGTGAGGTCAATTTTAGGAGCAGTGAATTTTATTAGGACCGTTGGAAACTTGCGAACTTTTGGTATAGTTCTTGACCGAATGCCTCTTTTTGTGTGGGCTGTCTTGATTACAGCAGTGTTACTCCTACTGTCTTTGCCCGTTTTAGCCGGCGCAATTACAATGTTATTAACTGATCGTAATTTAAATTCCTCTTTCTACGATCCCAGAGGGGGCGGAGATCCTATTTTATATCAACATTTATTC
